# Synthesis of spiropyridazine-benzosultams by the [4 + 2] annulation reaction of 3-substituted benzoisothiazole 1,1-dioxides with 1,2-diaza-1,3-dienes

**DOI:** 10.3762/bjoc.20.29

**Published:** 2024-02-14

**Authors:** Wenqing Hao, Long Wang, Jinlei Zhang, Dawei Teng, Guorui Cao

**Affiliations:** 1 College of Chemical Engineering, Qingdao University of Science and Technology, 53 Zhengzhou Lu, Qingdao 266042, China

**Keywords:** [4 + 2] annulation reaction, 1,2-diaza-1,3-dienes, spiro-benzosultams, 3-substituted benzoisothiazole 1,1-dioxides

## Abstract

A simple and efficient method for the synthesis of spiropyridazine-benzosultams has been developed by means of [4 + 2] annulation reaction of 3-substituted benzoisothiazole 1,1-dioxides with 1,2-diaza-1,3-dienes. This approach displays advantages such as mild reaction conditions, wide substrate range tolerance, simple operation, compatibility with gram-scale preparation.

## Introduction

Spirobenzosultams have various biological activities [1–3] such as antiviral, anticancer, antimicrobial, antimalarial, and antileukemia, and are widely used in the pharmaceutical field [4–6]. Pyridazine drugs have also shown high pharmaceutical activity. Many types of pyridazine drugs have been listed for antibacterial, anti-inflammatory, and other purposes [7–10]. Nowadays, a range of transformations to spirobenzosultams have been established using *N*-sulfonyl ketimines as suitable three-carbon synthons in [3 + 2] and [3 + 3] annulations [11–15]. However, methods using *N*-sulfonyl ketimines as dienophiles in [4 + 2] annulation reactions to afford biologically important spiro compounds are still limited to date [16]. 1,2-Diaza-1,3-dienes [17–27], which can be readily generated in situ from α-halogeno hydrazones, have been extensively applied in recent years as versatile building blocks in inverse-electron-demand Diels–Alder (IEDDA) reactions [28–30] to construct diverse nitrogen-containing heterocycles.

**Scheme 1:**
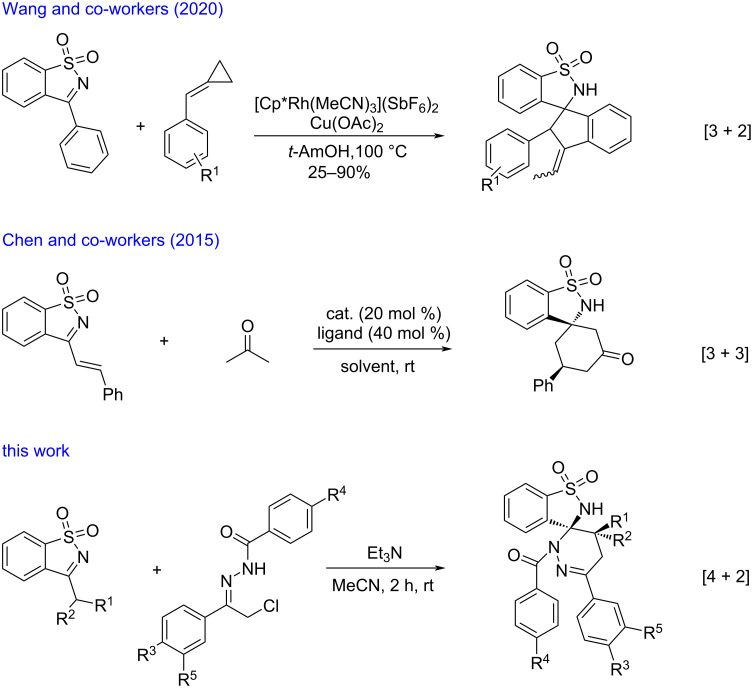
Comparision of previous work with this work.

Inspired by the potential biological activity of pyridazines and in continuation of our work on the synthesis of spirobenzosultams [31–33], we herein report a highly diastereoselective route for the synthesis of spiropyridazine-benzosultams through [4 + 2] annulation reactions of 3-substituted benzoisothiazole 1,1-dioxides with 1,2-diaza-1,3-dienes (Scheme 1).

## Results and Discussion

To initiate our studies, 3-ethylbenzo[*d*]isothiazole 1,1-dioxide (**1a**) and α-halogeno hydrazone **2a** were selected as the model substrates. Our aim was to explore the possibility of enamine–iminium tautomerism of *N*-sulfonyl ketimine and its subsequent [4 + 2] annulation reaction with 1,2-diaza-1,3-diene in the presence of Et_3_N (2.0 equiv) in diethyl ether at room temperature (Table 1, entry 1). However, no product was detected under these conditions. We then replaced diethyl ether with toluene, which resulted in the desired spiropyridazine-benzosultam **3aa** with 10% yield and high diastereoselectivity (Table 1, entry 2). Encouraged by this result, we tested several solvents to determine their effect on the [4 + 2] annulation reaction. Acetonitrile showed the best result, providing the highest yield (Table 1, entries 3–7). We also investigated the performance of other organic and inorganic bases, but they did not improve the yield (Table 1, entries 8–12). The structure of spiropyridazine-benzosultam **3aa** was determined by ^1^H NMR, ^13^C NMR, HRMS analysis and single-crystal X-ray crystallography [33]. Further experiments conducted with different reaction times revealed that the reaction was complete within 2 hours (Table 1, entry 14). We then explored the effect of the temperature on the reaction and found that 25 °C was the most suitable temperature, resulting in a 91% yield (Table 1, entry 14). Lower temperatures of 0 °C and 10 °C led to decreased yields of 24% and 49%, respectively (Table 1, entries 16 and 17). Increasing the temperature beyond 25 °C resulted in the formation of impurities and a decrease in the yield of the target compound (Table 1, entries 18 and 19). Finally, the optimal reaction conditions were determined as follows: **1a** (1.0 mmol), **2a** (1.5 mmol), Et_3_N (2.0 mmol), in acetonitrile at 25 °C with stirring for 2 hours (Table 1, entry 14).

**Table 1:** The effects of solvents, bases, reaction time and temperature on the [4 + 2] annulation reaction^a^.

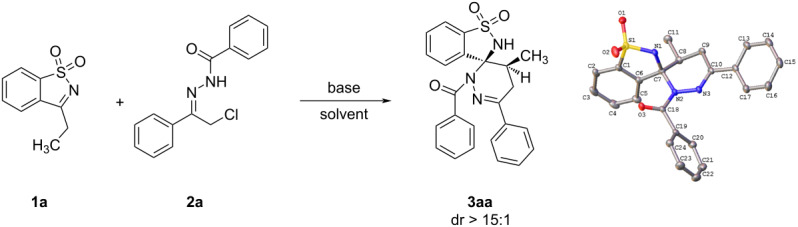					

Entry	Solvent	Base	Time (h)	Temperature (°C)	Yield (%)

1	Et_2_O	Et_3_N	1.5	25	0
2	toluene	Et_3_N	1.5	25	10
3	DCM	Et_3_N	1.5	25	58
4	THF	Et_3_N	1.5	25	62
5	MeCN	Et_3_N	1.5	25	70
6	DMF	Et_3_N	1.5	25	68
7	MeOH	Et_3_N	1.5	25	63
8	MeCN	DMAP	1.5	25	51
9	MeCN	DIPEA	1.5	25	59
10	MeCN	DBU	1.5	25	66
11	MeCN	NaH	1.5	25	59
12	MeCN	Cs_2_CO_3_	1.5	25	60
13	MeCN	Et_3_N	1.0	25	43
14	MeCN	Et_3_N	2.0	25	91
15	MeCN	Et_3_N	2.5	25	85
16	MeCN	Et_3_N	2.0	0	24
17	MeCN	Et_3_N	2.0	10	49
18	MeCN	Et_3_N	2.0	35	85
19	MeCN	Et_3_N	2.0	50	81

^a^Reaction conditions: **1a** (1.0 mmol), **2a** (1.5 mmol), base (2.0 mmol), solvent (10.0 mL).

**Scheme 2:**
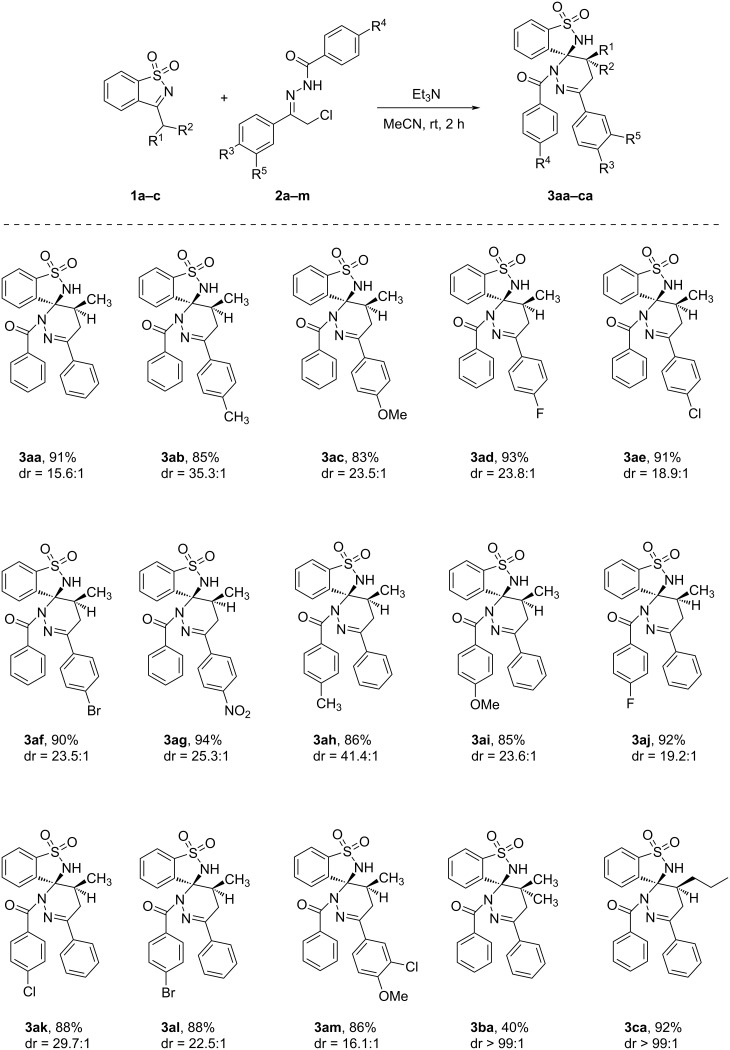
The effects of substituent groups on the [4 + 2] annulation reaction. Reaction conditions: **1** (1.0 mmol), **2** (1.5 mmol), Et_3_N (2.0 mmol), MeCN (10.0 mL), 25 °C, 2.0 h.

With the optimized reaction conditions in hand, we next investigated the scope of the current reaction (Scheme 2). As shown in Scheme 2, a series of α-halogeno hydrazones **2b–l** was tested, resulting in the expected spiropyridazine-benzosultams **3ab–al** in good to excellent yields with high diastereoselectivity. For *N*-benzoyl hydrazones **2b–g**, the electronic effect of R^3^ group on the [4 + 2] annulation reaction was significant. Electron-withdrawing groups gave relatively higher yields than electron-donating groups (Scheme 2, **3aa**–**af**). Remarkably, the nitro group gave the corresponding product **3ag** in 94% yield. It was found that electron-donating groups afforded relatively higher dr values than electron-withdrawing groups (Scheme 2, **3aa**–**af**). The effect of the R^4^ group was also detected and a similar result was observed as for the R^3^ group (Scheme 2, **3ah**,**ai** vs **3aj**,**ak**,**al**). To further expand the substrate scope, we next tested other 3-substituted benzoisothiazole 1,1-dioxides **1a–c**. As seen from Scheme 2, dienophiles with a bulky and branched isopropyl group (**1b**) could also be employed in the reaction. However, the yield of the corresponding product **3ba** was obviously lower than **3aa** and **3ca** generated from dienophiles bearing linear alkyl groups (Scheme 2, **3ba** vs **3aa** and **3ca**).

**Scheme 3:**
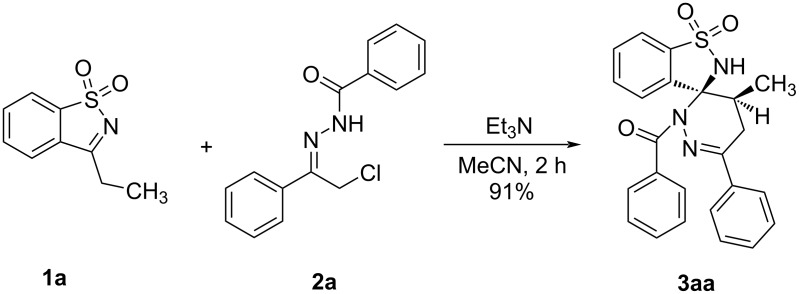
Gram-scale synthesis of **3aa**.

**Scheme 4:**
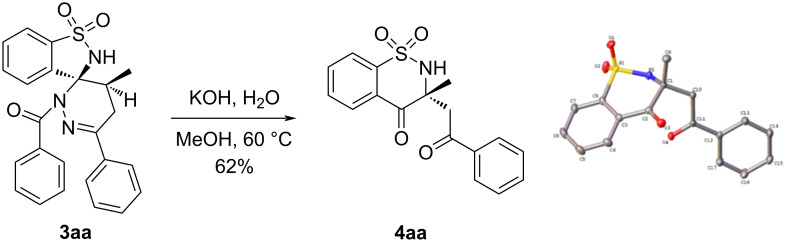
The transformation of **3aa**.

A gram-scale reaction was subsequently conducted to investigate the scalability of the experiment. The reaction of 3-ethylbenzo[*d*]isothiazole 1,1-dioxide (**1a**, 1.0 g) and α-halogeno hydrazone **2a** (2.1 g) afforded **3aa** (2.0 g) in 91% yield (Scheme 3) [34]. Finally, we focused on the transformation of **3aa**. When **3aa** was treated with KOH and H_2_O in methanol at 60 °C, the 3,3-disubsitituted-1,2-benzothiazin-4-one **4aa** [35] was isolated in 62% yield (Scheme 4).

**Scheme 5:**
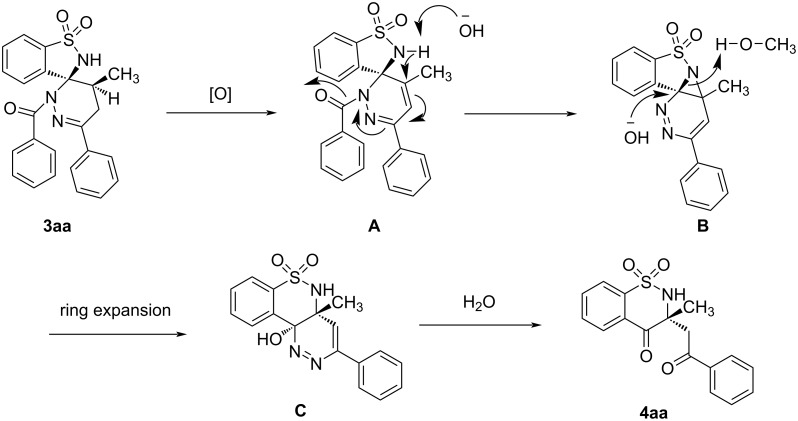
The reaction mechanism of the reaction from **3aa** to **4aa**.

On the basis of the transformation of **3aa** to **4aa**, a tentative reaction mechanism is proposed. As shown in Scheme 5, the spiropyridazine-benzosultam **3aa** was firstly oxidized to intermediate **A**. Next, an aziridine was formed with the hydrolysis of the amide bond under basic conditions. Finally, the ring expansion led to intermediate **C** which was then hydrolyzed to **4aa**.

## Conclusion

In conclusion, we have developed a [4 + 2] annulation reaction of 3-substituted benzo[*d*]isothiazole 1,1-dioxides with 1,2-diaza-1,3-dienes for the efficient preparation of spiropyridazine-benzosultams. The electronic effects of substituents and the influence of steric hindrance on the reaction were explored. The configuration of the product was determined by X-ray single crystal diffraction. This method has the advantages of mild reaction conditions, wide substrate scope, and high regioselectivity.

## Supporting Information

File 1 Experimental part, NMR and HRMS spectra.

## Data Availability

All data that supports the findings of this study is available in the published article and/or the supporting information to this article. The data generated and analyzed during this study is openly available in Cambridge Crystallographic Data Centre at https://doi.org/10.5517/ccdc.csd.cc2hf5kp (**3aa**) and https://doi.org/10.5517/ccdc.csd.cc2hf5lq (**4aa**).
