# Supplementary material for: Synthesis of spiropyridazine-benzosultams by the [4 + 2] annulation reaction of 3-substituted benzoisothiazole 1,1-dioxides with 1,2-diaza-1,3-dienes
Source: Beilstein J Org Chem. 2024 Feb 14;20:280–6. doi: 10.3762/bjoc.20.29 (PMC10877075; doi:10.3762/bjoc.20.29)
Supplement: File 1 — Experimental part, NMR and HRMS spectra. [file Beilstein_J_Org_Chem-20-280-s001.pdf]

BEILSTEIN JOURNAL OF ORGANIC CHEMISTRY

Supporting Information

for

Synthesis of spiropyridazine-benzosultams by the [4 + 2] annulation reaction of 3-substituted benzoisothiazole 1,1-dioxides with 1,2-diaza-1,3-dienes

Wenqing Hao, Long Wang, Jinlei Zhang, Dawei Teng and Guorui Cao

Beilstein J. Org. Chem. **2024,** *20,* 280–286. doi:10.3762/bjoc.20.29

Experimental part, NMR and HRMS spectra

License and Terms: This is a supporting information file under the terms of the Creative Commons Attribution License (https://creativecommons.org/licenses/by/4.0). Please note that the reuse, redistribution and reproduction in particular requires that the author(s) and source are credited and that individual graphics may be subject to special legal provisions.
The license is subject to the *Beilstein Journal of Organic Chemistry* terms and conditions: (https://www.beilstein-journals.org/bjoc/terms)

Table of Contents

1. General information..S2

2. General procedure for the synthesis of compound **3** and **4**................................S2–S3

3. Characterization data of compound **3** and **4**...S3–S11

4. Crystallographic information of **3aa** and **4aa** S12–S23

5. The ^{1}H NMR, ^{13}C NMR ^{19}F NMR and HRMS spectra of compound **3** and **4** S24–S48

6. ^{1}H NMR chromatograms of compound **3** and its diastereoisomer..................S49–S55

General information

All chemicals and reagents were purchased from commercial sources and used as received unless otherwise specified. Compounds **1a–c**, **2a–l** were synthesized according to the previous literature [1, 2]. All reactions were monitored by TLC. Melting points were recorded on a RY-1 microscopic melting apparatus and were uncorrected. Chromatography refers to open column chromatography on silica gel (100–200 mesh). ^{1}H NMR spectra were recorded on 500 MHz and ^{13}C NMR spectra were recorded on 125 MHz by using a Bruker Avance 500M spectrometer. Chemical shifts were reported in parts per million (δ) relative to tetramethylsilane (TMS). Mass spectra were performed on an Ultima Global spectrometer with an ESI source. The X-ray single-crystal diffraction was performed on Saturn 724+ instrument.

General procedure for the synthesis of spiropyridazine-benzosultams (3aa was taked as the example)

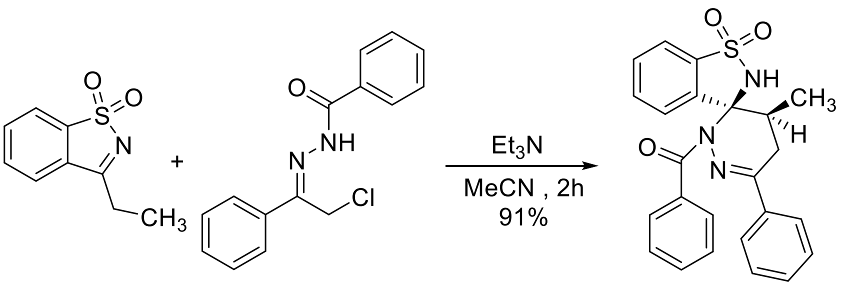

1a **2a** **3aa**

Triethylamine (0.15 g, 1.54 mmol, 2.00 equiv) was added dropwise into the 3-ethylbenzo[*d*]isothiazole 1,1-dioxide (**1a**, 0.15 g, 0.77 mmol, 1.00 equiv) dissolved in MeCN (7.7 mL). The resulting mixture was stirred for 5 min at room temperature and then α-halogenohydrazone **2a** (0.31 g, 1.15 mmol, 1.50 equiv) was added slowly into the reaction system. The reaction mixture was stirred at room temperature for 2 h and the progress of reaction was monitored continuously by TLC with ethyl acetate/petroleum ether (3:1) eluent system. Concentrated under reduced pressure and purified by column chromatography to obtain anticipated product **3aa** (0.30 g, 91%).

General procedure for the synthesis of 1,2-benzothiazin-3,3-disubsitituted-4-one 4aa

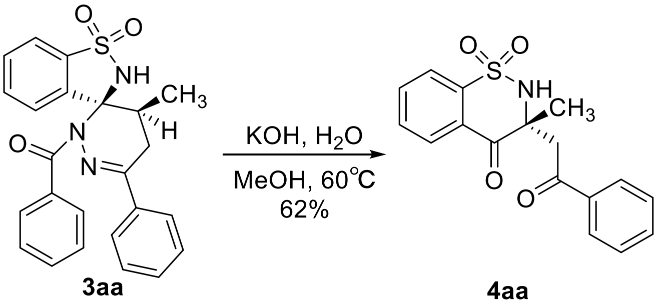

3aa **4aa**

A mixture of compound **3aa** (150 mg, 0.35 mmol, 1.00 equiv), KOH (39 mg, 0.70 mmol, 2.50 equiv), H_2O (12.6 mg, 0.70 mmol, 2.00 equiv) were dissolved in MeOH (11.0 mL), The reaction mixture was stirred at 60 ℃ for 12 h and the progress of reaction was monitored continuously by TLC. Concentrated under reduced pressure and purified by column chromatography with petroleum ether/ethyl acetate (8:1) to obtain 1,2-benzothiazin-3,3-disubsitituted-4-one **4aa** (71.7 mg, 62%) as white solid.

References

1. Liu, T.; Feng, X.; Du, H. *Tetrahedron. Lett.*, **2022**, 111, 154202. doi: 10.1016/j.tetlet.2022.154202
2. Chen, B.; Chu, W. D.; Liu, Q. Z. *RSC. Adv.*, **2019**, 9, 1487-1490. doi: 10.1039/C8RA08909D

Characterization data of compound 3 and 4

1) ((3*R**,4'*S**)-4'-Methyl-1,1-dioxido-6'-phenyl-4',5'-dihydro-2*H*,2'*H*-spiro[benzo[*d*]isothiazole-3,3'-pyridazin]-2'-yl)(phenyl)methanone (**3aa**).

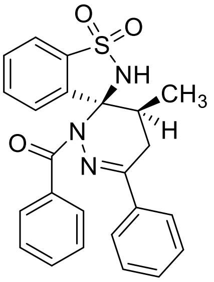

Yield: 91%. White solid; m.p.:179-180°C; ^{1}H NMR (500 MHz, $CDCl_3$) δ 7.88 (d, J = 7.5 Hz, 1H), 7.79-7.77 (m, 2H), 7.65-7.56 (m, 4H), 7.51-7.48 (m, 1H), 7.42-7.34 (m, 6H), 5.06 (s, 1H), 2.94 (dd, J = 17.0, 4.0 Hz, 1H), 2.66-2.60 (m, 1H), 2.58-2.52 (m, 1H), 0.95 (d, J = 6.5 Hz, 3H); ^{13}C NMR (125 MHz, $CDCl_3$) δ 170.7, 147.0, 140.2, 136.3, 136.0, 134.6, 134.1, 131.5, 130.4, 130.2, 130.0, 128.7, 127.7, 125.6, 121.8, 121.1, 77.0, 36.3, 27.9, 14.7; HRMS (ESI-TOF+): m/z calculated for $C_{24}H_{21}N_3O_3S$, $[M+Na]^+$: 454.1201, found: 454.1201.

2) ((3*R**,4'*S**)-4'-Methyl-1,1-dioxido-6'-(*p*-tolyl)-4',5'-dihydro-2*H*,2'*H*-spiro[benzo[*d*]isothiazole-3,3'-pyridazin]-2'-yl)(phenyl)methanone (**3ab**)

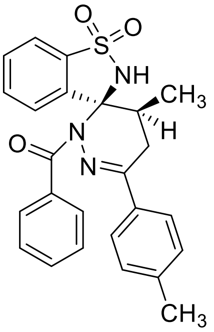

Yield: 85%. White solid; m.p.:136-138°C; ^{1}H NMR (500 MHz, DMSO) δ 8.80 (s, 1H), 7.83 (d, J = 8.0 Hz, 1H), 7.69-7.66 (m, 2H), 7.63-7.60 (m, 3H), 7.51-7.48 (m, 1H), 7.45-7.42 (m, 4H), 7.16 (d, J = 8.0 Hz, 2H), 2.98 (dd, J = 18.0, 4.5 Hz, 1H), 2.72-2.67 (m, 1H), 2.46-2.43 (m, 1H), 2.28 (s, 3H), 0.69 (d, J = 6.5 Hz, 3H); ^{13}C NMR (125 MHz, DMSO) δ 169.3, 146.0, 139.6, 138.9, 136.1, 136.0, 133.7, 133.4, 130.4, 129.6, 129.2, 129.0, 127.4, 125.2, 121.8, 120.6, 75.5, 34.9, 27.0, 20.8, 13.9; HRMS (ESI-TOF+): m/z calculated for $C_{25}H_{23}N_3O_3S$, $[M+Na]^+$: 468.1358, found: 468.1358.

3) ((3*R**,4'*S**)-6'-(4-Methoxyphenyl)-4'-methyl-1,1-dioxido-4',5'-dihydro-2*H*,2'*H*-spiro[benzo[*d*]isothiazole-3,3'-pyridazin]-2'-yl)(phenyl)methanone (**3ac**)

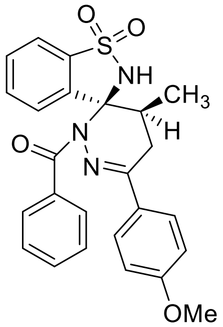

Yield: 83%. White solid; m.p.:136-138°C; ^{1}H NMR (500 MHz, DMSO) δ 8.78 (s, 1H), 7.83 (d, J = 7.5 Hz, 1H), 7.69-7.61 (m, 5H), 7.51-7.42 (m, 5H), 6.91 (d, J = 9.0 Hz, 2H), 3.75 (s, 3H), 2.97 (dd, J = 17.5, 4.5 Hz, 1H), 2.71-2.66 (m, 1H), 2.48-2.41 (m, 1H), 0.69 (d, J = 6.5 Hz, 3H); ^{13}C NMR (125 MHz, DMSO) δ 169.2, 160.2, 145.7, 139.7, 136.2, 136.0, 133.4, 130.4, 129.6, 129.2, 128.9, 127.4, 126.8, 121.7, 120.6, 113.8, 75.4, 55.2, 34.9, 27.0, 13.9; HRMS (ESI-TOF+): m/z calculated for $C_{25}H_{23}N_3O_4S$, $[M+Na]^+$: 484.1307, found: 484.1301.

4) ((3*R**,4'*S**)-6'-(4-Fluorophenyl)-4'-methyl-1,1-dioxido-4',5'-dihydro-2*H*,2'*H*-spiro[benzo[*d*]isothiazole-3,3'-pyridazin]-2'-yl)(phenyl)methanone (**3ad**)

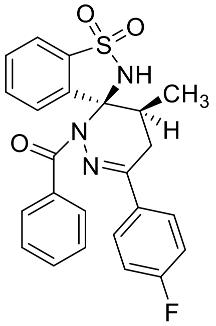

Yield: 93%. White solid; m.p.:200-202°C; ^{1}H NMR (500 MHz, DMSO) δ 8.79 (s, 1H), 7.84 (d, J = 7.6 Hz, 1H), 7.69-7.68 (m, 2H), 7.63-7.56 (m, 5H), 7.52-7.43 (m, 3H), 7.20 (t, J = 8.7 Hz, 2H), 3.00 (dd, J = 17.8, 4.2 Hz, 1H), 2.72-2.70 (m, 1H), 2.48-2.45 (m, 1H), 0.69 (d, J = 6.4 Hz, 3H); ^{13}C NMR (125 MHz, $CDCl_3$) δ 169.3, 162.7（d, $^1J_{C\text{-}F}$=246.3 HZ）, 145.2, 139.4, 136.0, 133.4, 132.9, 130.6, 129.7, 129.2, 127.5, 121.8, 120.6, 115.5, 115.3, 75.4, 34.8, 27.1, 13.9; ^{19}F NMR (376 MHz, DMSO) δ -111.93; HRMS (ESI-TOF+): m/z calculated for $C_{24}H_{20}FN_3O_3S$, $[M+Na]^+$: 472.1107, found: 472.1109.

5) ((3*R**,4'*S**)-6'-(4-Chlorophenyl)-4'-methyl-1,1-dioxido-4',5'-dihydro-2*H*,2'*H*-spiro[benzo[*d*]isothiazole-3,3'-pyridazin]-2'-yl)(phenyl)methanone (**3ae**)

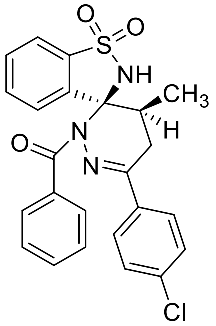

Yield: 91%. White solid; m.p.:144-146°C; ^{1}H NMR (500 MHz, $CDCl_3$) δ 7.86 (d, J = 7.5 Hz, 1H), 7.75 (d, J = 7.5 Hz, 2H), 7.65-7.57 (m, 2H), 7.51-7.48 (m, 3H), 7.42 – 7.36 (m, 3H), 7.30 (d, J = 8.5 Hz, 2H), 5.16 (s, 1H), 2.89 (dd, J = 16.5, 3.0 Hz, 1H), 2.63-2.57 (m, 1H), 2.56-2.52 (m, 1H), 0.94 (d, J = 6.0 Hz, 3H); ^{13}C NMR (125 MHz, $CDCl_3$) δ 170.6, 145.8, 140.0, 136.2, 135.8, 134.6, 134.1, 131.5, 130.4, 130.3, 128.9, 127.7, 126.9, 121.8, 121.1, 36.3, 27.8, 14.6; HRMS (ESI-TOF+): m/z calculated for $C_{24}H_{20}ClN_3O_3S$, $[M+Na]^+$: 488.0812, found: 488.0808.

6) ((3*R**,4'*S**)-6'-(4-Bromophenyl)-4'-methyl-1,1-dioxido-4',5'-dihydro-2*H*,2'*H*-spiro[benzo[*d*]isothiazole-3,3'-pyridazin]-2'-yl)(phenyl)methanone (**3af**)

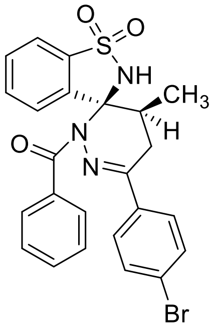

Yield: 90%. White solid; m.p.:152-154°C; ^{1}H NMR (500 MHz, $CDCl_3$) δ 7.86 (d, J = 7.5 Hz, 1H), 7.75 (d, J = 7.5 Hz, 2H), 7.65-7.57 (m, 2H), 7.51-7.45 (m, 3H), 7.42-7.36 (m, 5H), 5.14 (s, 1H), 2.88 (dd, J = 17.0, 3.5 Hz, 1H), 2.63-2.57 (m, 1H), 2.56-2.51 (m, 1H), 0.94 (d, J = 6.5 Hz, 3H); ^{13}C NMR (125 MHz, $CDCl_3$) δ 170.6, 145.9, 140.0, 136.3, 135.0, 134.6, 134.1, 131.9, 131.5, 130.3, 127.7, 127.1, 124.2, 121.8, 121.1, 36.3, 27.8, 14.6; HRMS (ESI-TOF+): m/z calculated for $C_{24}H_{20}BrN_3O_3S$, $[M+Na]^+$: 532.0306, found: 532.0300.

7) ((3*R**,4'*S**)-4'-Methyl-6'-(4-nitrophenyl)-1,1-dioxido-4',5'-dihydro-2*H*,2'*H*-spiro[benzo[*d*]isothiazole-3,3'-pyridazin]-2'-yl)(phenyl)methanone (**3ag**)

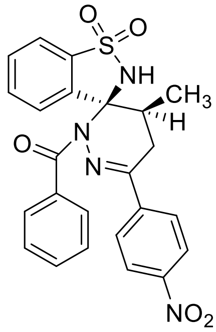

Yield: 94%. White solid; m.p.:210-212℃; ^{1}H NMR (500 MHz, DMSO) δ 8.86 (s, 1H), 8.22 (d, J = 9.0 Hz, 2H), 7.86 (d, J = 7.5 Hz, 1H), 7.76 (d, J = 9.0 Hz, 2H), 7.71-7.63 (m, 5H), 7.55-7.52 (m, 1H), 7.48-7.45 (m, 2H), 3.08 (dd, J = 18.0, 4.5 Hz, 1H), 2.80-2.75 (m, 1H), 2.57-2.54 (m, 1H), 0.71 (d, J = 6.5 Hz, 3H); ^{13}C NMR (125 MHz, DMSO) δ 169.3, 147.5, 144.4, 142.4, 139.1, 136.0, 135.5, 133.5, 130.9, 129.8, 129.3, 127.6, 126.3, 123.8, 121.8, 120.7, 75.5, 34.7, 27.1, 13.8; HRMS (ESI-TOF+): m/z calculated for $C_{24}H_{20}N_4O_5S$, $[M+Na]^+$:499.1052, found: 499.1056.

8) ((3*R**,4'*S**)-4'-Methyl-1,1-dioxido-6'-phenyl-4',5'-dihydro-2*H*,2'*H*-spiro[benzo[*d*]isothiazole-3,3'-pyridazin]-2'-yl)(*p*-tolyl)methanone (**3ah**)

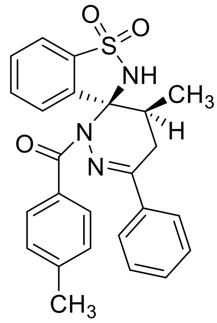

Yield: 86%. White solid; m.p.:194-196℃; ^{1}H NMR (500 MHz, DMSO) δ 8.78 (s, 1H), 7.83 (d, J = 7.0 Hz, 1H), 7.66 (s, 2H), 7.60-7.56 (m, 5H), 7.37 (s, 3H), 7.25 (d, J = 7.0 Hz, 2H), 2.99 (d, J = 17.5 Hz, 1H), 2.71-2.70 (m, 1H), 2.37 (s, 3H), 0.69 (d, J = 5.5 Hz, 3H); ^{13}C NMR (125 MHz, DMSO) δ 169.1, 145.8, 140.5, 139.6, 136.5, 136.0, 133.4, 133.0, 129.6, 129.3, 128.5, 128.0, 125.3, 121.7, 120.6, 75.5, 34.9, 27.2, 21.1, 13.9; HRMS (ESI-TOF+): m/z calculated for $C_{25}H_{23}N_3O_3S$, $[M+Na]^+$:468.1358, found: 468.1355.

9) (4-Methoxyphenyl)((3*R**,4'*S**)-4'-methyl-1,1-dioxido-6'-phenyl-4',5'-dihydro-2*H*,2'*H*-spiro[benzo[*d*]isothiazole-3,3'-pyridazin]-2'-yl)methanone (**3ai**)

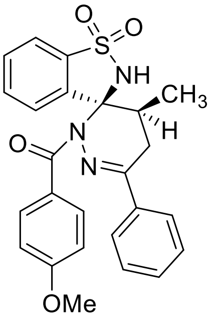

Yield: 85%. White solid; m.p.:192-194°C；^{1}H NMR (500 MHz, DMSO) δ 8.75 (s, 1H), 7.82 (d, J = 7.5 Hz, 1H), 7.67-7.60 (m, 7H), 7.39-7.37 (m, 3H), 6.98 (d, J = 8.5 Hz, 2H), 3.82 (s, 3H), 2.99 (dd, J = 17.8, 4.3 Hz, 1H), 2.74-.69 (m, 1H), 0.69 (d, J = 6.4 Hz, 3H); ^{13}C NMR (125 MHz, DMSO) δ 168.6, 161.2, 145.5, 139.6, 136.5, 136.0, 133.4, 131.9, 129.5, 129.2, 128.5, 127.6, 125.3, 121.7, 120.6, 112.7, 75.5, 55.3, 34.9, 27.2, 14.0; HRMS (ESI-TOF+): m/z calculated for $C_{25}H_{23}N_3O_4S$, $[M+Na]^+$:484.1307, found: 484.1306.

10) (4-Fluorophenyl)((3*R**,4'*S**)-4'-methyl-1,1-dioxido-6'-phenyl-4',5'-dihydro-2*H*,2'*H*-spiro[benzo[*d*]isothiazole-3,3'-pyridazin]-2'-yl)methanone (**3aj**)

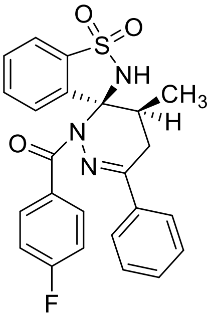

Yield: 92%. White solid; m.p.:176-178°C；^{1}H NMR (500 MHz, DMSO) δ 8.79 (s, 1H), 7.84 (d, J = 7.7 Hz, 1H), 7.74-7.67 (m, 4H), 7.63-7.60 (m, 1H), 7.57-7.55 (m, 2H), 7.38-7.37 (m, 3H), 7.29 (t, J = 8.8 Hz, 2H), 3.01 (dd, J = 17.8, 4.5 Hz, 1H), 2.74-2.70 (m, 1H), 0.69 (d, J = 6.5 Hz, 3H); ^{13}C NMR (125 MHz, DMSO) δ 168.2, 163.2（d, $^{1}J_{C\text{-}F}$=293.3 HZ）, 146.3, 139.4, 136.3, 136.0, 133.4, 132.3, 132.1, 132.0, 129.7, 129.4, 128.5, 125.3, 121.8, 120.6, 114.5, 114.4, 75.5, 34.8, 27.1, 13.9; ^{19}F NMR (376 MHz, DMSO) δ -109.44; HRMS (ESI-TOF+): m/z calculated for $C_{24}H_{20}FN_3O_3S$, $[M+Na]^+$: 472.1107, found: 472.1104.

11) (4-Chlorophenyl)((3*R**,4'*S**)-4'-methyl-1,1-dioxido-6'-phenyl-4',5'-dihydro-2*H*,2'*H*-spiro[benzo[*d*]isothiazole-3,3'-pyridazin]-2'-yl)methanone (**3ak**)

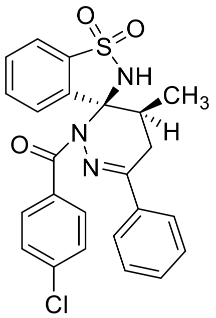

Yield: 88%. White solid; m.p.:188-190°C; ^{1}H NMR (500 MHz, DMSO) δ 8.80 (s, 1H), 7.84 (d, J = 7.5 Hz, 1H), 7.70-7.62 (m, 5H), 7.55-7.52 (m, 4H), 7.38 (s, 3H), 3.01 (d, J = 17.5 Hz, 1H), 2.71-2.70 (m, 1H), 0.69 (d, J = 6.0 Hz, 3H); ^{13}C NMR (125 MHz, DMSO) δ 168.2, 146.6, 139.4, 136.2, 135.9, 135.2, 134.8, 133.5, 131.2, 129.7, 129.4, 128.5, 127.6, 125.3, 121.8, 120.6, 75.5, 34.8, 27.1, 13.9; HRMS (ESI-TOF+): m/z calculated for $C_{24}H_{20}ClN_3O_3S$, $[M+Na]^+$: 488.0812, found: 488.0805.

12) (4-Bromophenyl)((3*R**,4'*S**)-4'-methyl-1,1-dioxido-6'-phenyl-4',5'-dihydro-2*H*,2'*H*-spiro[benzo[*d*]isothiazole-3,3'-pyridazin]-2'-yl)methanone (**3al**)

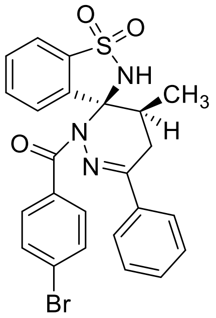

Yield: 88%. White solid; m.p.:184-186°C; ^{1}H NMR (500 MHz, DMSO) δ 8.80 (s, 1H), 7.84 (d, J = 7.7 Hz, 1H), 7.70-7.66 (m, 4H), 7.63-7.55 (m, 5H), 7.39-7.38 (m, 3H), 3.01 (dd, J = 17.8, 4.2 Hz, 1H), 2.73-2.69 (m, 1H), 0.69 (d, J = 6.4 Hz, 3H); ^{13}C NMR (125 MHz, DMSO) δ 168.3, 146.6, 139.4, 136.2, 135.9, 135.2, 133.5, 131.4, 130.5, 129.7, 129.4, 128.5, 125.3, 124.1, 121.8, 120.6, 75.5, 34.8, 27.1, 13.9; HRMS (ESI-TOF+): m/z calculated for $C_{24}H_{20}BrN_3O_3S$, $[M+Na]^+$: 532.0306, found: 532.0313.

13) ((3*R**,4'*S**)-6'-(3-Chloro-4-methoxyphenyl)-4'-methyl-1,1-dioxido-4',5'-dihydro-2*H*,2'*H*-spiro[benzo[*d*]isothiazole-3,3'-pyridazin]-2'-yl)(phenyl)methanone (**3am**)

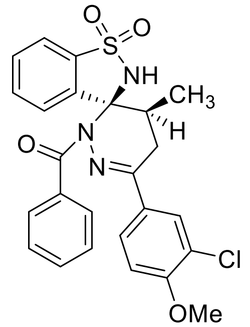

Yield: 86%. White solid; m.p.:190-192°C; ^{1}H NMR (500 MHz, $CDCl_3$) δ 7.87 (d, J = 7.5 Hz, 1H), 7.76 (d, J = 7.6 Hz, 2H), 7.64-7.57 (m, 3H), 7.51-7.48 (m, 1H), 7.43-7.36 (m, 4H), 6.87 (d, J = 8.7 Hz, 1H), 3.90 (s, 3H), 2.86 (d, J = 13.5 Hz, 1H), 2.60-2.51 (m, 2H), 0.94 (d, J = 6.0 Hz, 3H); ^{13}C NMR (125 MHz, DMSO) δ 169.2, 144.6, 139.5, 136.1, 135.9, 133.4, 130.5, 129.8, 129.7, 129.2, 127.4, 126.7, 125.5, 121.8, 121.2, 120.7, 112.6, 75.4, 56.3, 34.9, 26.8, 13.9; HRMS (ESI-TOF+): m/z calculated for $C_{25}H_{22}ClN_3O_4S$, $[M+Na]^+$:518.0917, found: 518.0914.

14) (*R**)-(4',4'-Dimethyl-1,1-dioxido-6'-phenyl-4',5'-dihydro-2*H*,2'*H*-spiro[benzo[*d*]isothiazole-3,3'-pyridazin]-2'-yl)(phenyl)methanone (**3ba**)

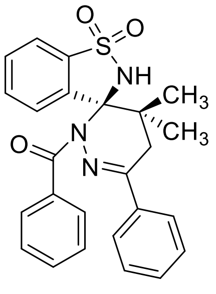

Yield: 40%. White solid; m.p.:242-244°C; ^{1}H NMR (500 MHz, DMSO) δ 8.85 (s, 1H), 7.87-7.86 (m, 1H), 7.64-7.63 (m, 5H), 7.53-7.45 (m, 5H), 7.36-7.35(m, 3H), 2.82 (s, 2H), 1.24 (s, 3H), 0.86 (s, 3H); ^{13}C NMR (125 MHz, DMSO) δ 170.0, 145.7, 137.3, 136.7, 136.3, 135.9, 132.2, 130.8, 129.7, 129.4, 129.3, 128.4, 127.6, 125.4, 124.2, 120.9, 78.2, 36.9, 34.9, 23.8, 23.0; HRMS (ESI-TOF+): m/z calculated for $C_{25}H_{23}N_3O_3S$, $[M+Na]^+$:468.1358, found: 468.1351.

15) ((3*R**,4'*S**)-1,1-Dioxido-6'-phenyl-4'-propyl-4',5'-dihydro-2*H*,2'*H*-spiro[benzo[*d*]isothiazole-3,3'-pyridazin]-2'-yl)(phenyl)methanone (**3ca**)

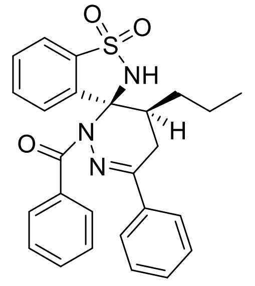

Yield: 92%. White solid; m.p.:174-176°C； ^{1}H NMR (500 MHz, DMSO) δ 8.82 (s, 1H), 7.84 (d, J = 7.4 Hz, 1H), 7.72-7.57 (m, 7H), 7.50-7.43 (m, 3H), 7.35 (s, 3H), 3.07 (d, J = 14.3 Hz, 1H), 2.61 (s, 1H), 2.46-2.43 (m, 1H), 1.36-1.28 (m, 2H), 1.04-1.03 (m, 1H), 0.69-0.67 (m, 4H); ^{13}C NMR (125 MHz, DMSO) δ 169.4, 145.7, 139.5, 136.5, 136.3, 136.1, 133.4, 130.5, 129.7, 129.2, 128.4, 127.4, 125.3, 121.9, 120.6, 75.4, 30.1, 24.8, 18.8, 13.9; HRMS (ESI-TOF+): m/z calculated for $C_{26}H_{25}N_3O_3S$, $[M+Na]^+$:482.1514, found: 482.1511.

16) (*R**)-3-Methyl-3-(2-oxo-2-phenylethyl)-2,3-dihydro-4*H*-benzo[*e*][1,2]thiazin-4-one 1,1-dioxide (**4aa**)

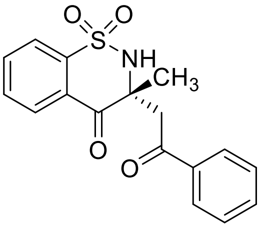

Yield: 62%. White solid; m.p.:156-158°C； ^{1}H NMR (500 MHz, DMSO) δ 8.69 (s, 1H), 8.05 (d, J = 7.9 Hz, 1H), 7.93-7.86 (m, 5H), 7.64 (t, J = 7.3 Hz, 1H), 7.50 (t, J = 7.6 Hz, 2H),3.87 (dd, J = 85.0, 18.5 Hz, 2H), 1.70 (s, 3H);^{13}C NMR (125 MHz, DMSO) δ 196.0, 195.1, 141.5, 135.6, 134.2, 133.6, 132.8, 129.7, 128.6, 128.2, 128.2, 122.1, 63.9, 51.4, 26.1; HRMS (ESI-TOF+): m/z calculated for $C_{17}H_{15}NO_4S$, $[M+Na]^+$:352.0619, found: 352.0611.

Crystallographic information of 3aa and 4aa

Table S1. The crystal date and structure refinement for **3aa**

Identification code	3aa
Empirical formula	$C_{24}H_{21}N_3O_3S$
Formula weight	431.50
Temperature/K	170.00(10)
Crystal system	triclinic
Space group	P-1
a/Å	7.6579(2)
b/Å	8.0465(2)
c/Å	17.9509(4)
α/°	77.190(2)
β/°	80.016(2)
γ/°	88.400(2)
Volume/Å^3	1062.19(5)
Z	2
ρcalcg/cm^3	1.349
μ/mm^{-1}	0.184
F(000)	452.0
Crystal size/mm^3	0.25 × 0.23 × 0.2
Radiation	MoKα (λ = 0.71073)
2θ range for data collection/°	4.724 to 61.346
Index ranges	-8 ≤ h ≤ 10, -9 ≤ k ≤ 11, -23 ≤ l ≤ 23
Reflections collected	15701
Independent reflections	5582 [R_{int} = 0.0231, R_{sigma} = 0.0284]
Data/restraints/parameters	5582/0/285
Goodness-of-fit on F^2	1.070
Final R indexes [I>=2σ (I)]	R_1 = 0.0384, wR_2 = 0.0976
Final R indexes [all data]	R_1 = 0.0479, wR_2 = 0.1027

Largest diff. peak/hole / e Å^{-3}	0.27/-0.45

Table S2. Fractional atomic coordinates ($\times10^4$) and equivalent isotropic displacement parameters ($Å^2\times10^3$) for **3aa**. U_{eq} is defined as 1/3 of of the trace of the orthogonalised U_{IJ} tensor.

Atom	X	y	z	U(eq)
S8	3035.4(4)	6742.5(4)	5508.6(2)	29.99(9)
O22	2353.3(12)	7201.5(11)	7386.6(6)	31.2(2)
O24	3243.7(14)	6262.6(14)	4770.4(6)	43.1(3)
O25	3742.7(14)	8369.5(13)	5493.5(7)	47.4(3)
N10	2836.3(14)	4352.9(12)	7535.0(6)	25.5(2)
N11	4098.9(14)	3256.5(13)	7848.6(6)	26.8(2)
N9	3780.2(14)	5224.9(13)	6145.0(6)	26.2(2)
C21	2684.1(15)	5861.1(15)	7802.3(7)	25.3(2)
C26	2843.3(16)	5740.3(16)	8627.1(7)	27.2(3)
C2	699.9(16)	5245.5(16)	6650.3(8)	27.6(3)
C12	4674.0(17)	2004.6(15)	7539.4(7)	28.4(3)
C1	2411.5(16)	4315.3(15)	6769.3(7)	25.1(2)
C7	842.1(16)	6509.4(16)	5988.6(8)	27.7(3)
C15	6159.3(17)	1056.8(15)	7864.3(8)	30.8(3)
C14	2254.3(18)	2413.6(16)	6755.7(8)	31.0(3)
C6	-590.1(18)	7478.2(18)	5766.3(9)	35.3(3)
C27	2183(2)	4364.5(19)	9216.4(9)	37.8(3)
C13	3994.2(19)	1528.2(16)	6882.7(8)	32.9(3)
C31	3538.8(18)	7141.1(18)	8814.7(8)	34.2(3)
C16	7283.3(19)	92.4(18)	7429.8(9)	38.0(3)
C3	-947.4(17)	4892.0(19)	7123.3(9)	36.3(3)
C20	6517(2)	1175.7(19)	8582.0(9)	39.6(3)
C17	8747(2)	-700.0(19)	7703.0(10)	44.0(4)

C5	-2226.9(19)	7104(2)	6234.4(10)	43.2(4)
C23	1683(2)	2141.3(19)	6022.1(9)	39.9(3)
C19	7979(2)	378(2)	8853.2(10)	48.0(4)
C18	9106(2)	-556.3(19)	8407.0(10)	46.0(4)
C4	-2391.4(18)	5838(2)	6903.2(11)	44.8(4)
C30	3584(2)	7153(2)	9579.4(9)	45.4(4)
C28	2204(2)	4410(2)	9980.4(9)	48.3(4)
C29	2906(2)	5795(2)	10158.9(9)	49.3(4)

Table S3. Anisotropic Displacement Parameters ($Å^2 \times 10^3$) for **3aa**. The Anisotropic displacement factor exponent takes the form: $-2\pi^2[h^2a^{*2}U_{11}+2hka^*b^*U_{12}+\cdots]$

Atom	U11	U22	U33	U23	U13	U12
S8	26.20(17)	27.31(17)	32.99(18)	-3.06(12)	-0.99(12)	5.20(12)
O22	35.1(5)	23.9(4)	37.7(5)	-10.0(4)	-11.4(4)	6.8(4)
O24	43.4(6)	53.1(6)	28.4(5)	-5.3(5)	-1.5(4)	18.6(5)
O25	37.8(6)	26.4(5)	69.9(8)	1.3(5)	-1.8(5)	-2.2(4)
N10	28.3(5)	21.3(5)	28.0(5)	-7.8(4)	-5.0(4)	3.9(4)
N11	27.5(5)	21.5(5)	30.1(6)	-5.0(4)	-3.2(4)	3.4(4)
N9	21.7(5)	25.3(5)	30.1(6)	-6.1(4)	-0.8(4)	4.1(4)
C21	20.7(6)	23.9(6)	32.4(7)	-10.2(5)	-2.4(5)	2.4(4)
C26	23.0(6)	28.3(6)	30.5(6)	-10.1(5)	-1.7(5)	5.2(5)
C2	22.4(6)	29.4(6)	34.5(7)	-14.5(5)	-4.4(5)	-0.8(5)
C12	32.9(7)	20.1(6)	29.6(6)	-4.7(5)	0.4(5)	1.3(5)
C1	24.8(6)	23.4(6)	27.1(6)	-7.7(5)	-1.9(4)	-1.2(4)
C7	23.4(6)	29.9(6)	33.6(7)	-14.0(5)	-6.1(5)	3.2(5)
C15	33.3(7)	18.7(6)	37.4(7)	-4.2(5)	-0.7(5)	2.3(5)
C14	36.8(7)	23.5(6)	33.3(7)	-8.9(5)	-3.7(5)	-3.4(5)
C6	33.2(7)	37.9(7)	43.1(8)	-20.0(6)	-16.5(6)	10.1(6)
C27	39.1(8)	34.7(7)	37.0(8)	-9.7(6)	3.2(6)	-1.1(6)
C13	45.9(8)	20.3(6)	33.5(7)	-9.4(5)	-5.7(6)	4.2(5)

C31	37.2(7)	32.0(7)	35.1(7)	-10.6(6)	-6.9(6)	0.6(5)
C16	38.9(8)	28.9(7)	46.1(9)	-12.7(6)	-1.8(6)	6.4(6)
C3	25.8(7)	39.4(8)	44.6(8)	-16.1(6)	1.6(5)	-7.3(5)
C20	44.8(8)	34.0(7)	39.2(8)	-8.2(6)	-6.7(6)	12.2(6)
C17	38.1(8)	31.4(7)	59.1(10)	-10.8(7)	0.6(7)	9.0(6)
C5	25.7(7)	51.0(9)	65.5(11)	-32.8(8)	-18.1(7)	9.5(6)
C23	48.2(9)	34.7(7)	42.5(8)	-17.4(6)	-11.1(6)	-2.5(6)
C19	52.2(10)	45.6(9)	46.0(9)	-6.1(7)	-15.6(7)	14.4(7)
C18	37.7(8)	33.7(8)	61.0(11)	-0.2(7)	-8.1(7)	10.3(6)
C4	19.5(6)	54.7(10)	66.7(11)	-31.7(8)	-0.7(6)	-4.5(6)
C30	52.6(9)	48.6(9)	42.1(9)	-20.7(7)	-13.3(7)	0.3(7)
C28	56.5(10)	48.6(9)	32.8(8)	-3.0(7)	4.2(7)	1.1(7)
C29	58.8(10)	60.5(11)	31.2(8)	-16.4(7)	-7.6(7)	6.6(8）

Table S4. Bond lengths for **3aa**.

Atom	Atom	Length/Å	Atom	Atom	Length/Å
S8	O24	1.4424(11)	C1	C14	1.5441(17)
S8	O25	1.4237(11)	C7	C6	1.3914(18)
S8	N9	1.6401(11)	C15	C16	1.3963(18)
S8	C7	1.7444(13)	C15	C20	1.386(2)
O22	C21	1.2156(15)	C14	C13	1.5215(19)
N10	N11	1.3968(14)	C14	C23	1.5188(19)
N10	C21	1.3954(15)	C6	C5	1.382(2)
N10	C1	1.4733(16)	C27	C28	1.383(2)
N11	C12	1.2866(16)	C31	C30	1.381(2)
N9	C1	1.4703(16)	C16	C17	1.384(2)
C21	C26	1.4881(17)	C3	C4	1.389(2)
C26	C27	1.3881(19)	C20	C19	1.383(2)
C26	C31	1.3899(18)	C17	C18	1.369(2)
C2	C1	1.5150(17)	C5	C4	1.381(3)

C2	C7	1.3721(19)	C19	C18	1.394(2)
C2	C3	1.3909(18)	C30	C29	1.373(2)
C12	C15	1.4832(18)	C28	C29	1.373(2)
C12	C13	1.4938(19)			

Table S5. Bond angles for **3aa**.

Atom	Atom	Atom	Angle/°	Atom	Atom	Atom	Angle/°
O24	S8	N9	109.80(6)	N9	C1	C14	112.56(10)
O24	S8	C7	111.67(6)	C2	C1	C14	111.32(10)
O25	S8	O24	115.71(7)	C2	C7	S8	110.54(9)
O25	S8	N9	112.00(6)	C2	C7	C6	123.06(13)
O25	S8	C7	111.31(6)	C6	C7	S8	126.37(11)
N9	S8	C7	94.38(6)	C16	C15	C12	120.07(13)
N11	N10	C1	120.73(9)	C20	C15	C12	121.13(12)
C21	N10	N11	112.67(9)	C20	C15	C16	118.72(13)
C21	N10	C1	120.44(10)	C13	C14	C1	109.54(10)
C12	N11	N10	119.94(11)	C23	C14	C1	113.01(11)
C1	N9	S8	114.88(8)	C23	C14	C13	111.60(11)
O22	C21	N10	121.11(11)	C5	C6	C7	117.42(14)
O22	C21	C26	121.56(11)	C28	C27	C26	119.86(14)
N10	C21	C26	117.25(10)	C12	C13	C14	111.12(11)
C27	C26	C21	122.70(12)	C30	C31	C26	120.19(14)
C27	C26	C31	119.28(13)	C17	C16	C15	120.64(15)
C31	C26	C21	117.75(12)	C4	C3	C2	118.33(15)
C7	C2	C1	114.37(11)	C19	C20	C15	120.40(14)
C7	C2	C3	119.15(12)	C18	C17	C16	120.32(14)
C3	C2	C1	126.43(13)	C4	C5	C6	120.20(13)
N11	C12	C15	114.87(12)	C20	C19	C18	120.22(16)
N11	C12	C13	124.61(12)	C17	C18	C19	119.69(14)

C15	C12	C13	120.48(11)	C5	C4	C3	121.82(14)
N10	C1	C2	110.71(10)	C29	C30	C31	120.08(15)
N10	C1	C14	106.06(10)	C29	C28	C27	120.38(15)
N9	C1	N10	110.76(10)	C30	C29	C28	120.20(15)
N9	C1	C2	105.53(10)				

Table S6. Hydrogen bonds for **3aa**.

D	H	A	d(D-H)/Å	d(H-A)/Å	d(D-A)/Å	D-H-A/°
N9	H9	O24[1]	0.824(18)	2.146(18)	2.9577(15)	168.5(17)

[1]1-X,1-Y,1-Z

Table S7. Hydrogen Atom Coordinates (Å × 10^4) and Isotropic Displacement Parameters ($Å^2 \times 10^3$) for **3aa**.

Atom	x	y	z	U(eq)
H14	1342.12	1896.76	7193.99	37
H6	-451.79	8343.57	5320.2	42
H27	1726.85	3414.21	9097.62	45
H13A	3815.53	303.34	6993.32	39
H13B	4866.8	1845.15	6413.18	39
H31	3975.34	8072.92	8424.24	41
H16	7046.07	-18.54	6952.18	46
H3	-1078.81	4043.53	7575.49	44
H20	5768.46	1795.49	8882.94	48
H17	9489.81	-1333.12	7406.87	53
H5	-3220.25	7707.59	6098.58	52
H23A	527.99	2614.46	5986.91	60
H23B	1644.67	942.73	6036.99	60
H23C	2516.41	2694.61	5578.57	60
H19	8211.01	465.63	9334.81	58
H18	10097.89	-1079.53	8586.75	55
H4	-3499.53	5612.47	7214.7	54

H30	4073.15	8082.32	9701.94	55
H28	1739.36	3497.41	10375.5	58
H29	2923.17	5813.51	10673.82	59
H9	4570(20)	4680(20)	5934(10)	46(5)

Table S8. The crystal date and structure refinement for **4aa**

Identification code	4aa
Empirical formula	$C_{17}H_{15}NO_4S$
Formula weight	329.36
Temperature/K	169.97(10)
Crystal system	monoclinic
Space group	Cc
a/Å	14.3259(2)
b/Å	12.33000(10)
c/Å	10.74620(10)
α/°	90
β/°	125.6130(10)
γ/°	90
Volume/Å^3	1543.17(3)
Z	4
ρ_{calc}g/cm^3	1.418
μ/mm^{-1}	2.047
F(000)	688.0
Crystal size/mm^3	0.25 × 0.18 × 0.15
Radiation	Cu Kα (λ = 1.54178)
2θ range for data collection/°	10.448 to 150.88
Index ranges	$-17 \leq h \leq 15$, $-15 \leq k \leq 15$, $-13 \leq l \leq 13$
Reflections collected	9397
Independent reflections	2351 [R_{int} = 0.0203, R_{sigma} = 0.0158]
Data/restraints/parameters	2351/2/213

Goodness-of-fit on F2	1.065
Final R indexes [I>=2σ (I)]	R1 = 0.0253, wR2 = 0.0651
Final R indexes [all data]	R1 = 0.0253, wR2 = 0.0652
Largest diff. peak/hole / e Å-3	0.19/-0.33

Table S9. Fractional atomic coordinates (×10^4) and equivalent isotropic displacement parameters (Å^2×10^3) for **4aa**. U_{eq} is defined as 1/3 of of the trace of the orthogonalised U_{IJ} tensor.

Atom	X	y	z	U(eq)
S1	8174.8(4)	5836.5(3)	7118.3(4)	23.69(13)
O1	8862.6(14)	6382.0(12)	6740.9(19)	33.3(4)
O2	8419.8(15)	6026.1(14)	8596.2(18)	36.1(4)
O3	5910.0(15)	4165.3(12)	2829.9(17)	30.0(4)
O4	4983.7(13)	4535.7(11)	4843.8(16)	26.5(3)
N1	6830.9(15)	6133.8(13)	5853.8(19)	21.5(3)
C1	6271.9(19)	5787.4(15)	4239(2)	21.1(4)
C2	6489.5(18)	4583.8(16)	4094(2)	22.6(4)
C3	7424(2)	3961.0(17)	5443(2)	24.6(4)
C4	7514(2)	2853.9(17)	5274(3)	32.8(5)
C5	8376(3)	2247.4(18)	6485(3)	39.9(6)
C6	9167(2)	2730.0(19)	7891(3)	39.7(5)
C7	9104(2)	3823.9(19)	8095(3)	32.2(5)
C8	8233.2(19)	4433.8(17)	6864(2)	23.7(4)
C9	6708(2)	6479.4(18)	3484(3)	29.4(4)
C10	4979.2(18)	5973.4(15)	3378(2)	22.8(4)
C11	4413.0(18)	5236.6(15)	3884(2)	21.7(4)
C12	3166.4(18)	5353.3(15)	3170(2)	22.9(4)
C13	2496(2)	6139.5(17)	2059(2)	28.7(4)
C14	1333(2)	6198.6(19)	1396(3)	33.0(5)
C15	824(2)	5495(2)	1835(3)	31.7(5)

C16	1492(2)	4713(2)	2957(3)	38.5(5)
C17	2647(2)	4642.2(19)	3609(3)	32.6(5)

Table S10. Anisotropic Displacement Parameters ($Å^2 \times 10^3$) for **4aa**. The Anisotropic displacement factor exponent takes the form: $-2\pi^2[h^2a^{*2}U_{11}+2hka^*b^*U_{12}+\cdots]$

Atom	U11	U22	U33	U23	U13	U12
S1	16.4(2)	26.2(2)	24.1(2)	-4.53(18)	9.35(18)	0.33(18)
O1	22.1(8)	30.5(7)	45.0(9)	-3.2(6)	18.3(7)	-4.6(6)
O2	26.4(9)	47.9(9)	23.4(8)	-8.4(6)	8.5(7)	7.5(7)
O3	32.4(9)	30.4(8)	25.1(8)	-6.2(5)	15.5(7)	-3.9(6)
O4	22.3(8)	26.2(6)	24.2(7)	4.2(6)	9.8(6)	1.0(6)
N1	18.0(9)	24.5(7)	21.5(8)	-3.8(6)	11.2(7)	-0.4(7)
C1	19.2(10)	22.3(9)	19.2(10)	-0.7(6)	9.7(8)	-0.9(7)
C2	23.6(10)	24.3(9)	23.7(10)	-3.5(7)	16.0(9)	-3.6(8)
C3	28.0(11)	23.7(9)	27.6(10)	-0.3(7)	19.3(9)	0.4(8)
C4	43.5(13)	24.3(10)	37.6(11)	0.3(8)	27.6(11)	0.7(9)
C5	53.2(16)	24.1(10)	48.8(14)	7.3(10)	33.3(13)	8.6(11)
C6	41.2(14)	34.3(12)	43.2(13)	15.3(10)	24.5(11)	14.1(10)
C7	26.6(12)	36.6(11)	29.6(11)	5.9(9)	14.3(9)	5.0(10)
C8	21.4(10)	25.7(8)	26.9(11)	3.2(7)	15.7(8)	4.2(8)
C9	32.5(12)	29.3(10)	30.5(10)	0.1(8)	20.7(9)	-4.7(9)
C10	18.8(11)	22.9(9)	22.7(10)	2.1(7)	9.9(9)	0.2(7)
C11	20.4(10)	21.3(8)	18.8(8)	-2.1(7)	8.8(7)	-1.7(7)
C12	19.8(10)	25.0(9)	20.5(9)	-2.0(7)	9.8(8)	-1.8(7)
C13	25.5(11)	26.7(10)	31.8(11)	4.3(8)	15.4(10)	1.2(8)
C14	23.6(11)	35.7(11)	32.8(11)	5.9(9)	12.4(9)	5.7(9)
C15	19.8(11)	42.2(11)	31.5(11)	-1.6(9)	14.0(9)	1.5(9)
C16	27.3(12)	49.1(13)	39.7(12)	9.9(10)	19.9(11)	-2.2(11)
C17	24.6(11)	38.2(12)	30.5(11)	9.4(9)	13.5(9)	1.2(9)

Table S11. Bond lengths for **4aa**.

Atom	Atom	Length/Å	Atom	Atom	Length/Å
S1	O1	1.4328(17)	C4	C5	1.381(3)
S1	O2	1.4321(17)	C5	C6	1.388(4)
S1	N1	1.6250(19)	C6	C7	1.378(3)
S1	C8	1.761(2)	C7	C8	1.396(3)
O3	C2	1.219(2)	C10	C11	1.515(3)
O4	C11	1.223(2)	C11	C12	1.484(3)
N1	C1	1.489(3)	C12	C13	1.397(3)
C1	C2	1.543(3)	C12	C17	1.398(3)
C1	C9	1.538(3)	C13	C14	1.381(3)
C1	C10	1.531(3)	C14	C15	1.382(3)
C2	C3	1.491(3)	C15	C16	1.398(3)
C3	C4	1.393(3)	C16	C17	1.373(3)
C3	C8	1.398(3)			

Table S12. Bond angles for **4aa**.

Atom	Atom	Atom	Angle/°	Atom	Atom	Atom	Angle/°
O1	S1	N1	109.42(9)	C5	C4	C3	120.5(2)
O1	S1	C8	108.32(9)	C4	C5	C6	120.6(2)
O2	S1	O1	118.19(10)	C7	C6	C5	120.3(2)
O2	S1	N1	107.25(9)	C6	C7	C8	118.8(2)
O2	S1	C8	109.25(10)	C3	C8	S1	119.68(15)
N1	S1	C8	103.42(9)	C7	C8	S1	118.61(17)
C1	N1	S1	118.76(13)	C7	C8	C3	121.7(2)
N1	C1	C2	112.98(16)	C11	C10	C1	113.94(16)
N1	C1	C9	110.34(16)	O4	C11	C10	119.45(18)
N1	C1	C10	107.56(16)	O4	C11	C12	121.13(17)
C9	C1	C2	108.20(16)	C12	C11	C10	119.39(17)
C10	C1	C2	109.12(16)	C13	C12	C11	122.04(18)

C10	C1	C9	108.55(17)	C13	C12	C17	119.2(2)
O3	C2	C1	118.23(18)	C17	C12	C11	118.75(18)
O3	C2	C3	120.57(18)	C14	C13	C12	120.0(2)
C3	C2	C1	121.12(17)	C13	C14	C15	120.6(2)
C4	C3	C2	118.46(19)	C14	C15	C16	119.6(2)
C4	C3	C8	118.1(2)	C17	C16	C15	120.1(2)
C8	C3	C2	123.47(18)	C16	C17	C12	120.5(2)

Table S13. Torsion angles for **4aa**.

A	B	C	D	Angle/˚	A	B	C	D	Angle/˚
S1	N1	C1	C2	48.8(2)	C2	C3	C8	C7	-179.25(19)
S1	N1	C1	C9	-72.42(19)	C3	C4	C5	C6	0.0(4)
S1	N1	C1	C10	169.34(12)	C4	C3	C8	S1	178.51(16)
O1	S1	N1	C1	63.91(16)	C4	C3	C8	C7	-0.7(3)
O1	S1	C8	C3	-90.06(18)	C4	C5	C6	C7	-0.2(4)
O1	S1	C8	C7	89.22(18)	C5	C6	C7	C8	-0.1(4)
O2	S1	N1	C1	-166.75(14)	C6	C7	C8	S1	-178.66(19)
O2	S1	C8	C3	139.95(17)	C6	C7	C8	C3	0.6(3)
O2	S1	C8	C7	-40.8(2)	C8	S1	N1	C1	-51.34(16)
O3	C2	C3	C4	-9.0(3)	C8	C3	C4	C5	0.4(3)
O3	C2	C3	C8	169.56(19)	C9	C1	C2	O3	-70.6(2)
O4	C11	C12	C13	-179.21(18)	C9	C1	C2	C3	106.2(2)
O4	C11	C12	C17	-0.2(3)	C9	C1	C10	C11	173.01(16)
N1	S1	C8	C3	25.99(18)	C10	C1	C2	O3	47.3(2)
N1	S1	C8	C7	-154.74(17)	C10	C1	C2	C3	-135.87(18)

N1	C1	C2	O3	166.93(18)	C10	C11	C12	C13	-1.3(3)
N1	C1	C2	C3	-16.3(3)	C10	C11	C12	C17	177.73(18)
N1	C1	C10	C11	-67.6(2)	C11	C12	C13	C14	178.27(19)
C1	C2	C3	C4	174.33(19)	C11	C12	C17	C16	-179.0(2)
C1	C2	C3	C8	-7.2(3)	C12	C13	C14	C15	0.8(3)
C1	C10	C11	O4	-3.7(3)	C13	C12	C17	C16	0.1(3)
C1	C10	C11	C12	178.37(16)	C13	C14	C15	C16	-0.3(4)
C2	C1	C10	C11	55.3(2)	C14	C15	C16	C17	-0.4(4)
C2	C3	C4	C5	179.0(2)	C15	C16	C17	C12	0.5(4)
C2	C3	C8	S1	0.0(3)	C17	C12	C13	C14	-0.7(3)

Table S14. Hydrogen Atom Coordinates (Å×10^4) and Isotropic Displacement Parameters (Å^2×10^3) for **4aa**.

Atom	x	y	z	U(eq)
H1	6500(30)	5970(20)	6230(30)	25(7)
H4	6988.56	2520.51	4338.32	39
H5	8427.35	1508.94	6358.19	48
H6	9742.99	2312.93	8699.59	48
H7	9631.14	4150.11	9035.33	39
H9A	6586.96	7233.16	3571.62	44
H9B	6295.84	6288.24	2421.97	44
H9C	7512.52	6348.71	3986.62	44
H10A	4850.3	6721.14	3521.42	27
H10B	4613.84	5864.38	2292.99	27
H13	2833.27	6622.84	1765.19	34
H14	888.06	6716.97	647.12	40
H15	40.55	5540.58	1387.71	38
H16	1154.89	4240.1	3261.34	46
H17	3087.5	4117.16	4348.64	39

The ^{1}H NMR, ^{13}C NMR, ^{19}F NMR and HRMS spectra of compound 3 and 4

1) ((3*R**,4'*S**)-4'-Methyl-1,1-dioxido-6'-phenyl-4',5'-dihydro-2*H*,2'*H*-spiro[benzo[*d*]isothiazole-3,3'-pyridazin]-2'-yl)(phenyl)methanone (**3aa**).

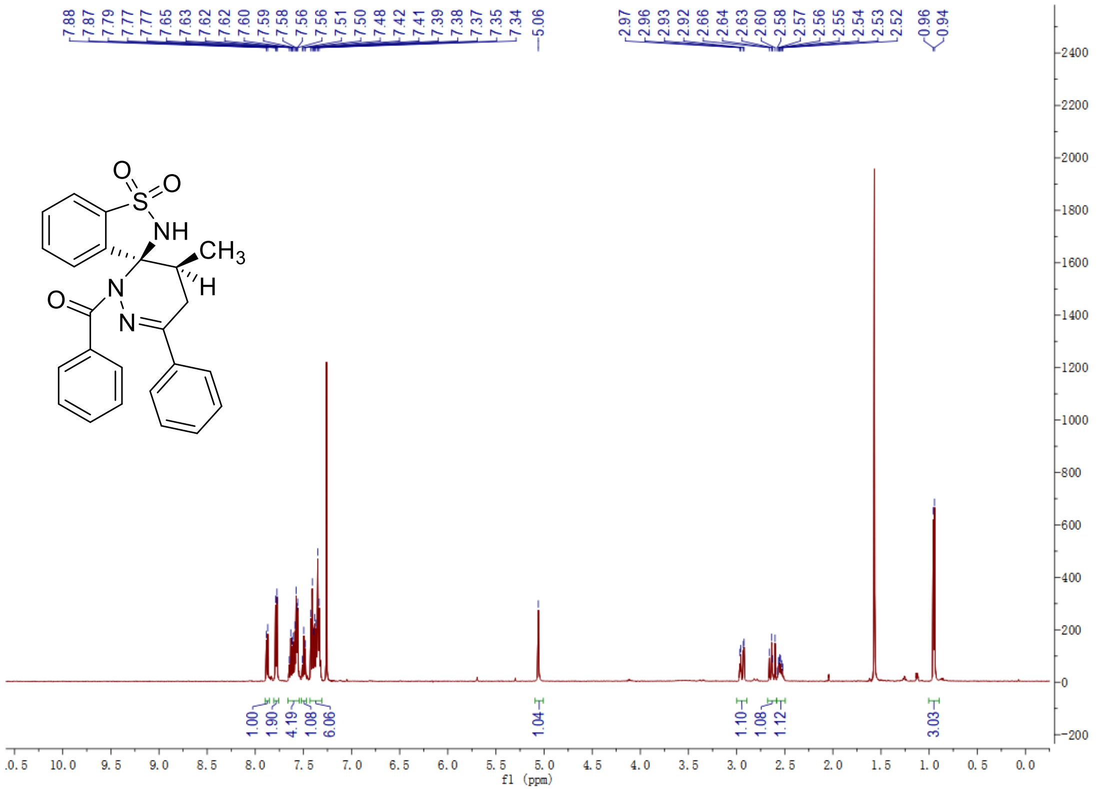

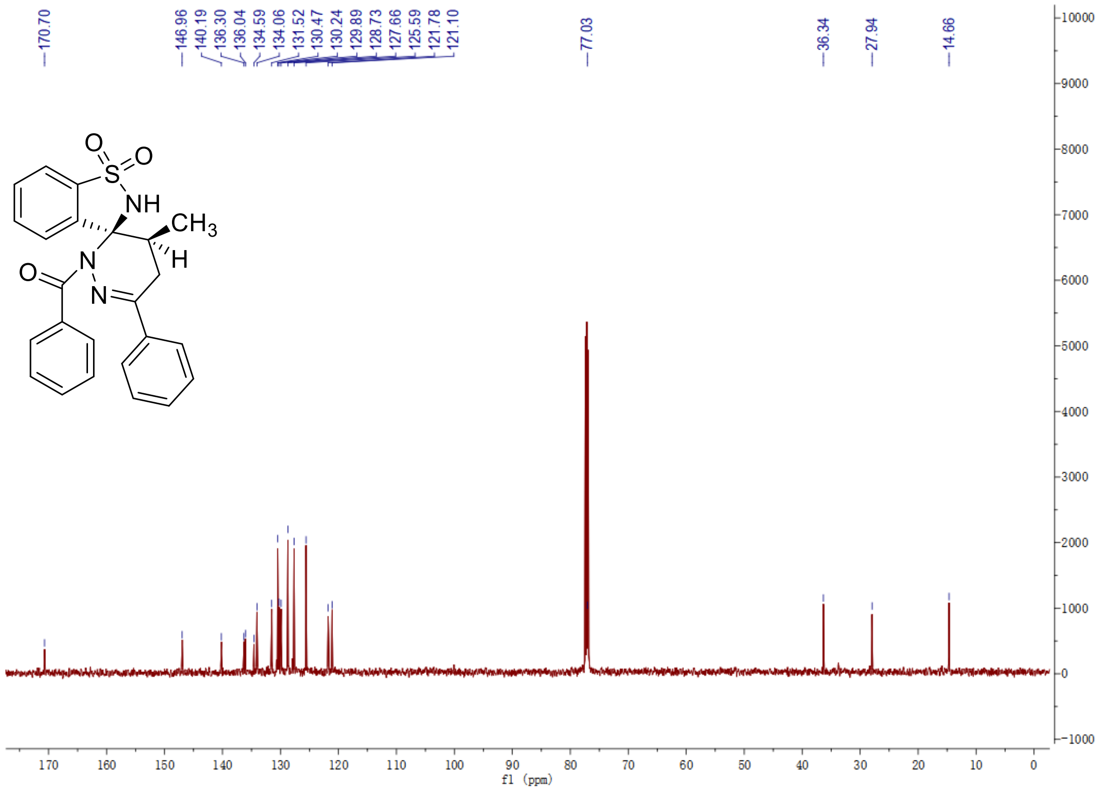

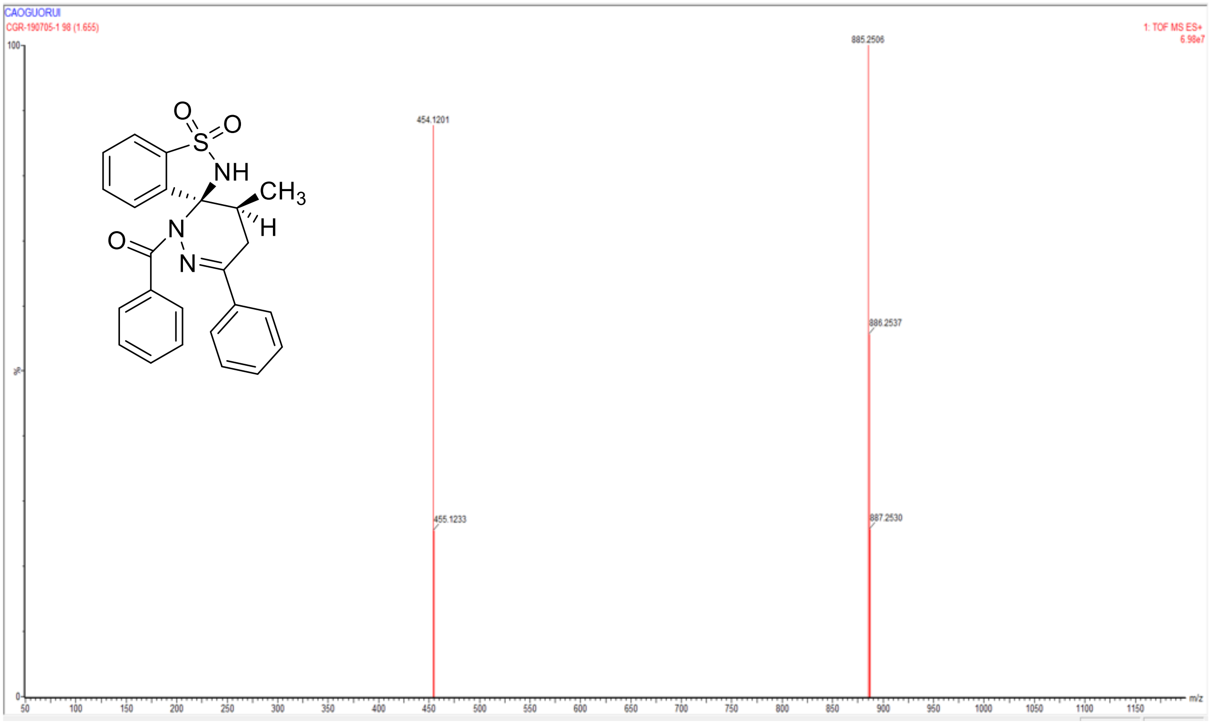

2) ((3*R**,4'*S**)-4'-Methyl-1,1-dioxido-6'-(*p*-tolyl)-4',5'-dihydro-2*H*,2'*H*-spiro[benzo[*d*]isothiazole-3,3'-pyridazin]-2'-yl)(phenyl)methanone (**3ab**)

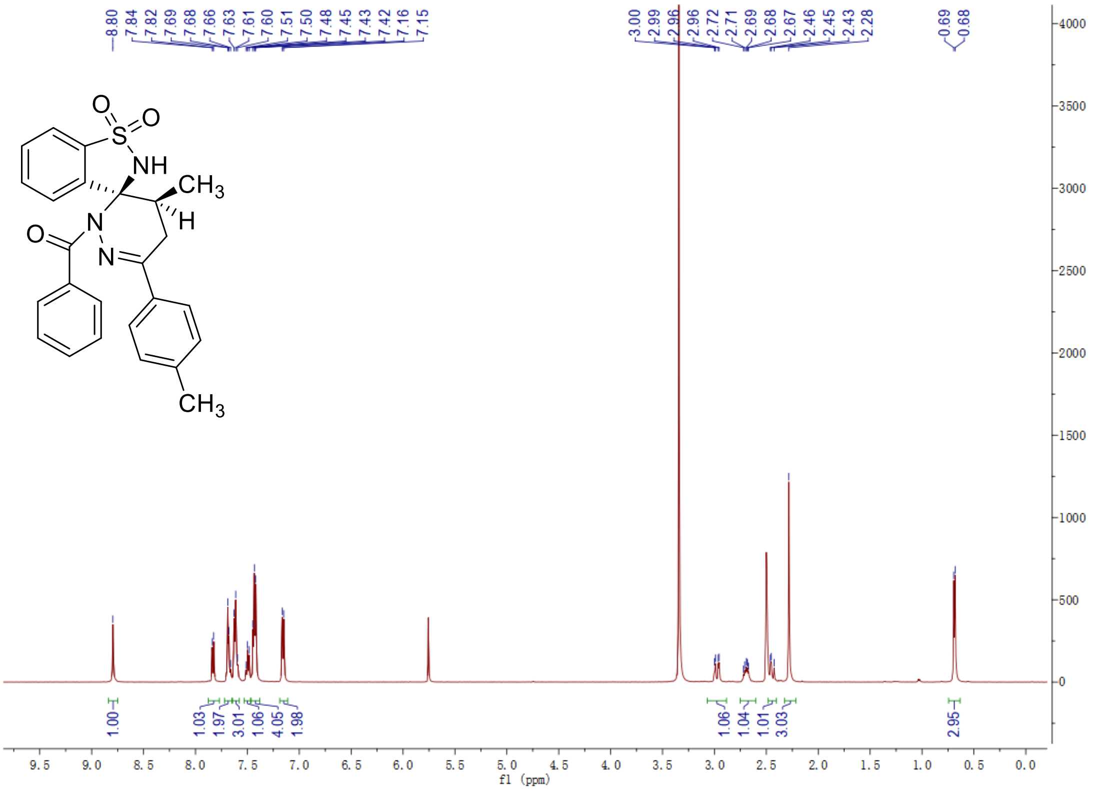

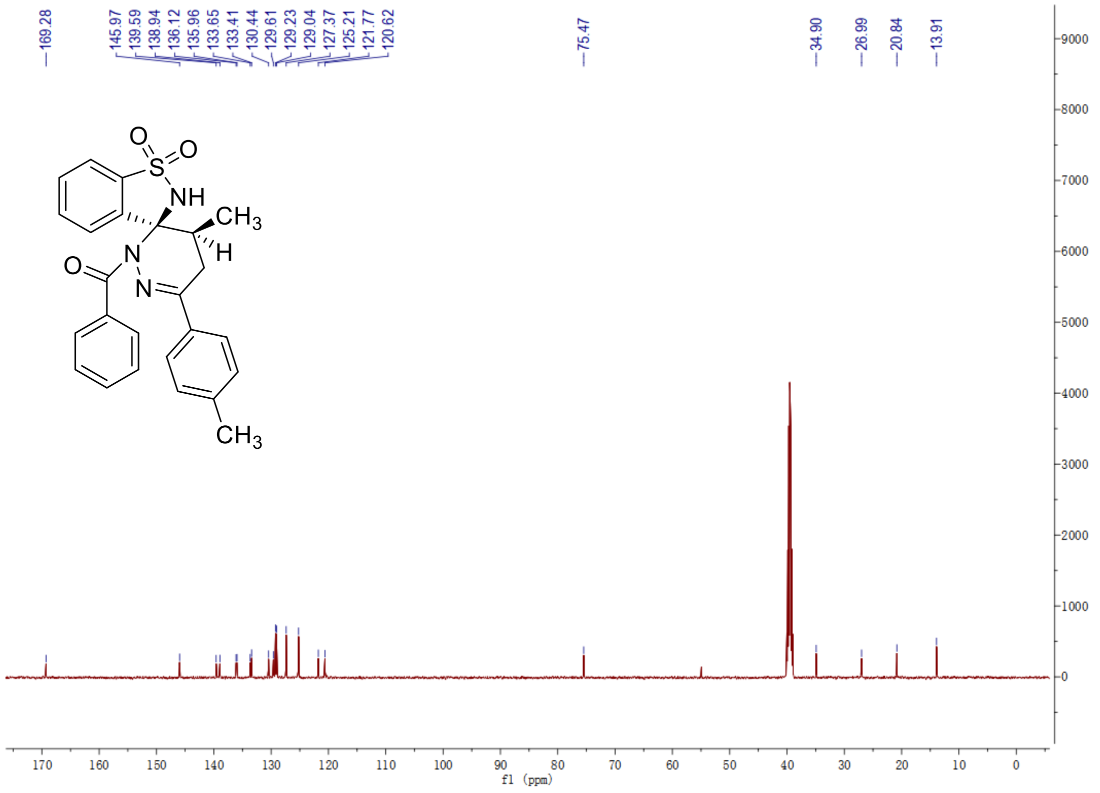

169.28
145.97
139.59
138.94
136.12
135.96
133.65
133.41
130.44
129.61
129.23
129.04
127.37
125.21
121.77
120.62
75.47
34.90
26.99
20.84
13.91
f1 (ppm)

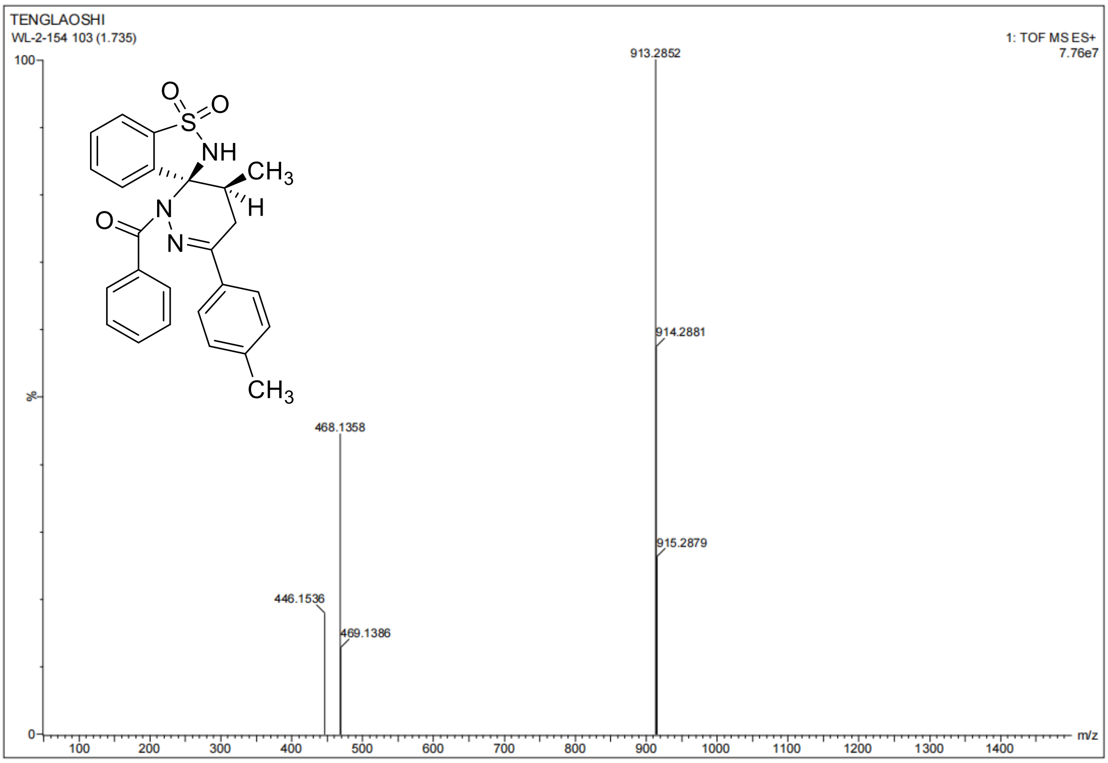

TENGLAOSHI
WL-2-154 103 (1.735)
1: TOF MS ES+
7.76e7
913.2852
914.2881
915.2879
468.1358
446.1536
469.1386
m/z
%

3) ((3*R**,4'*S**)-6'-(4-Methoxyphenyl)-4'-methyl-1,1-dioxido-4',5'-dihydro-2*H*,2'*H*-spiro[benzo[*d*]isothiazole-3,3'-pyridazin]-2'-yl)(phenyl)methanone (**3ac**)

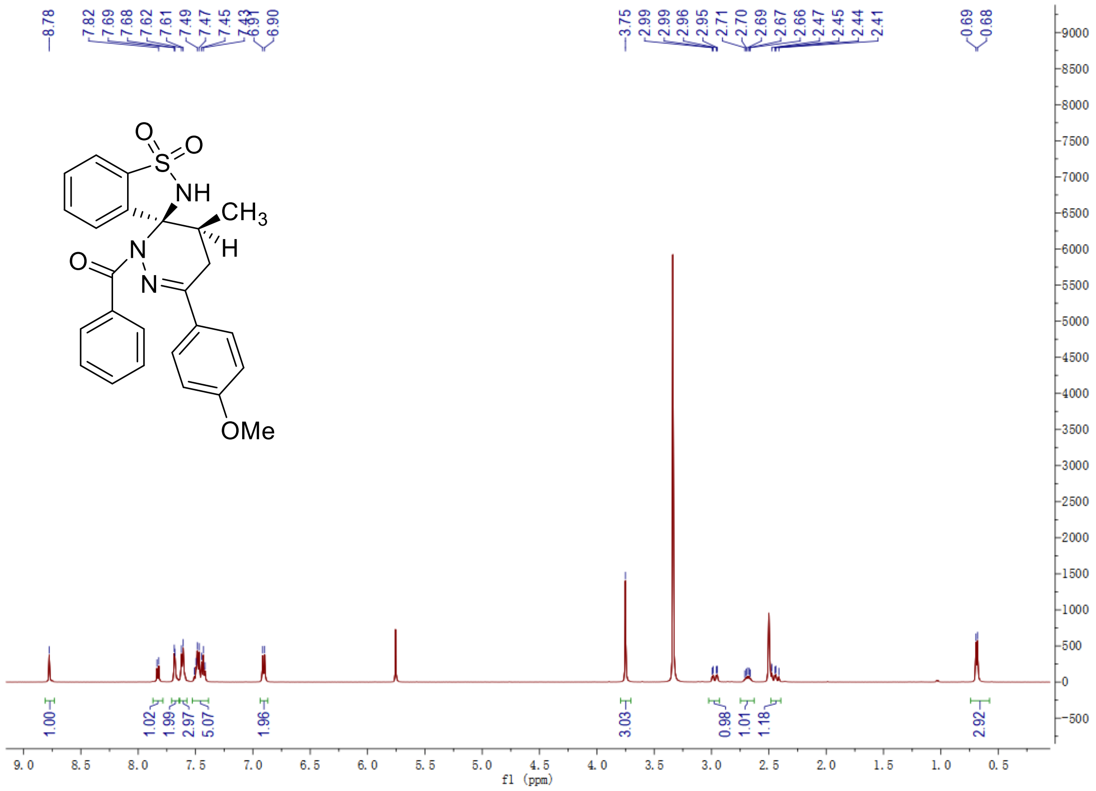

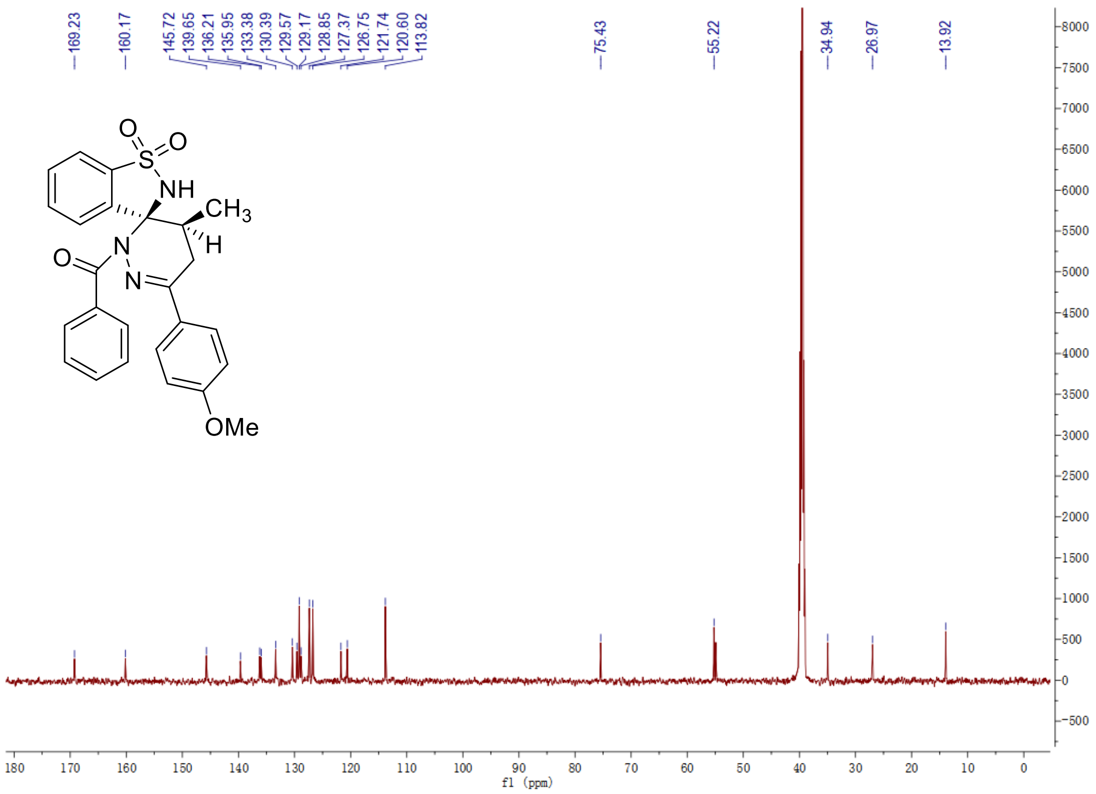

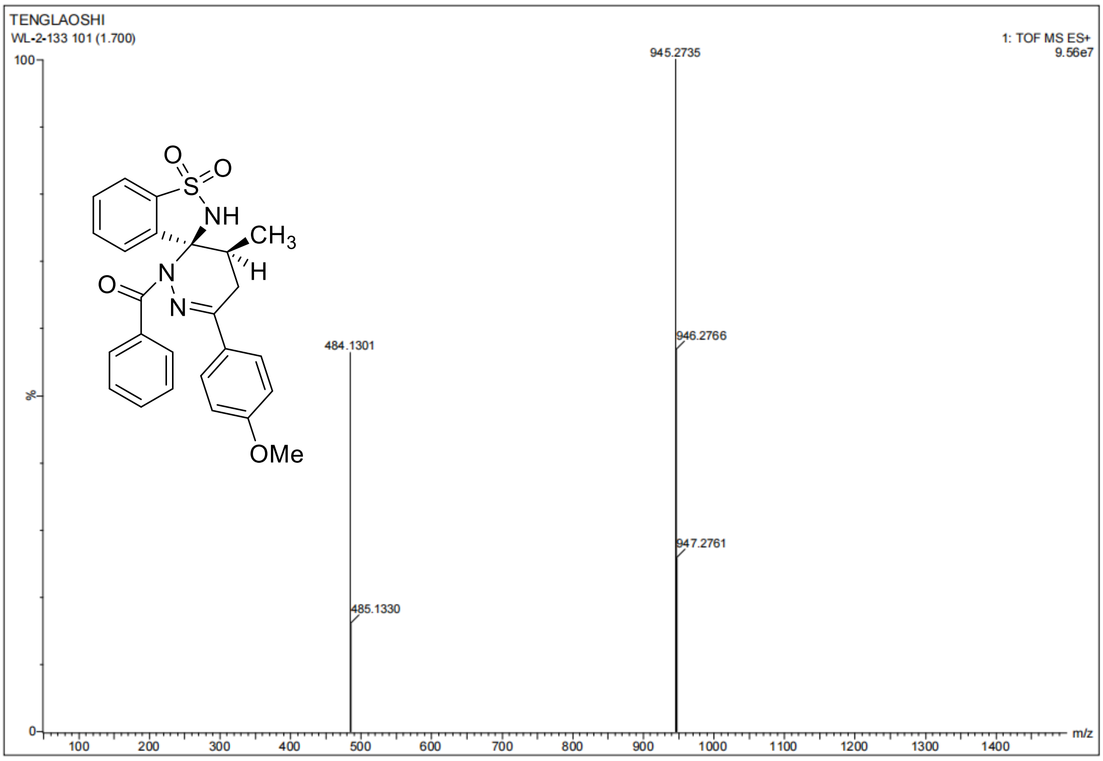

4) ((3*R**,4'*S**)-6'-(4-Fluorophenyl)-4'-methyl-1,1-dioxido-4',5'-dihydro-2*H*,2'*H*-spiro[benzo[*d*]isothiazole-3,3'-pyridazin]-2'-yl)(phenyl)methanone (**3ad**)

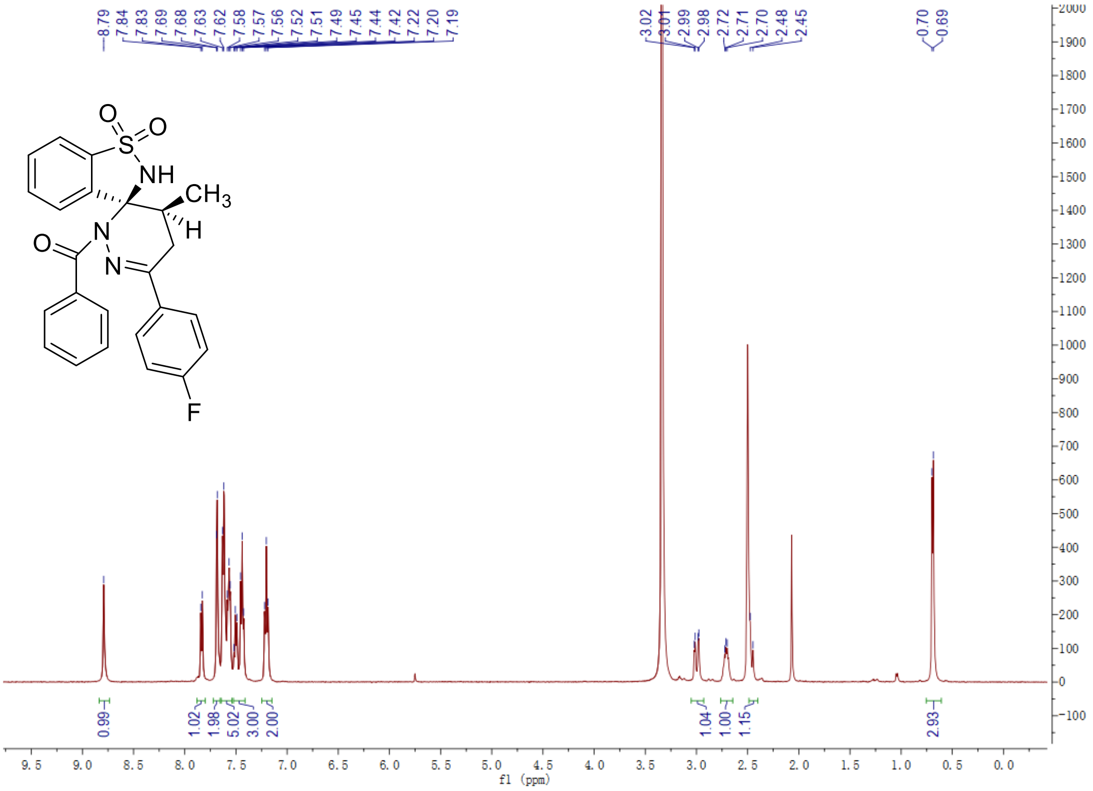

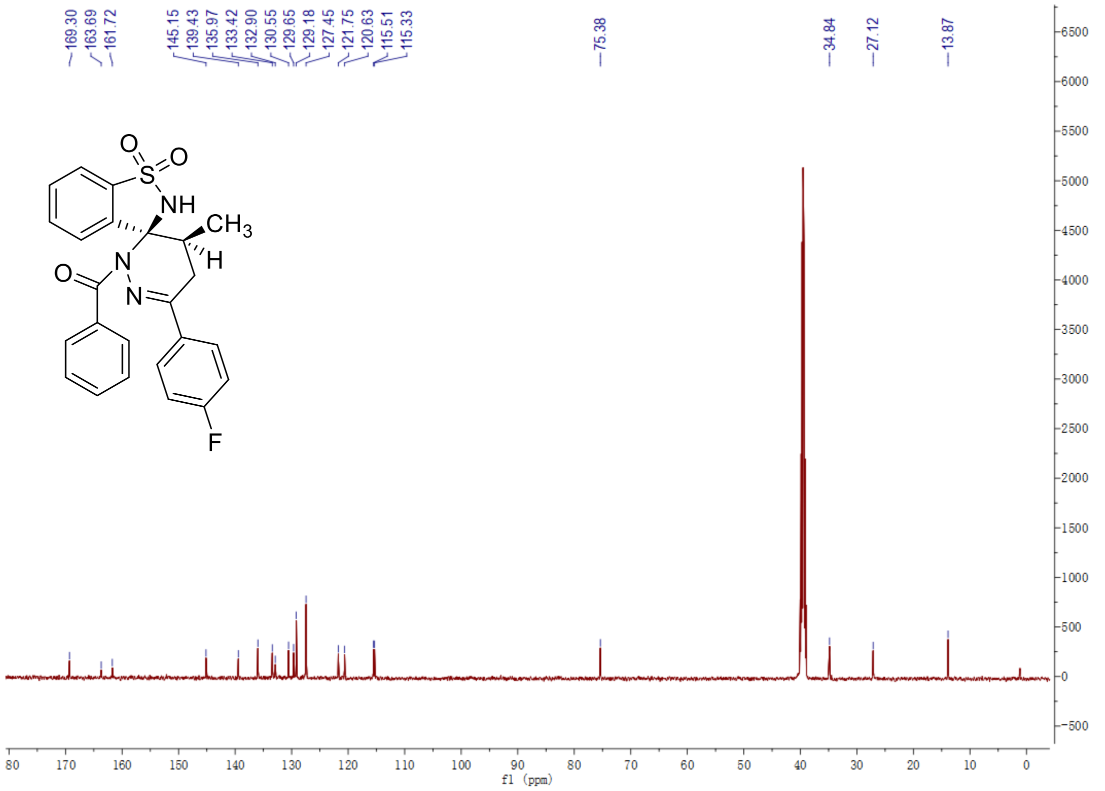
169.30
163.69
161.72
145.15
139.43
135.97
133.42
132.90
130.55
129.65
129.18
127.45
121.75
120.63
115.51
115.33
75.38
34.84
27.12
13.87
O
S
O
NH
CH3
H
N
O
N
F
f1 (ppm)

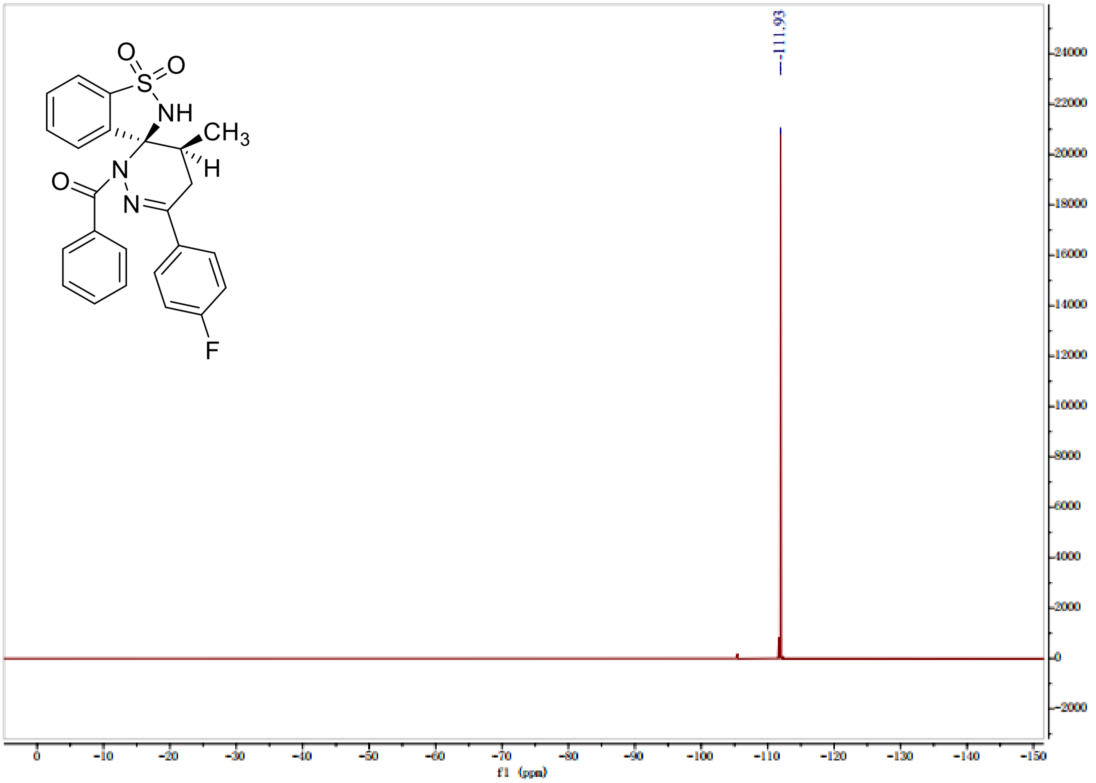
-111.93
O
S
O
NH
CH3
H
N
O
N
F
f1 (ppm)

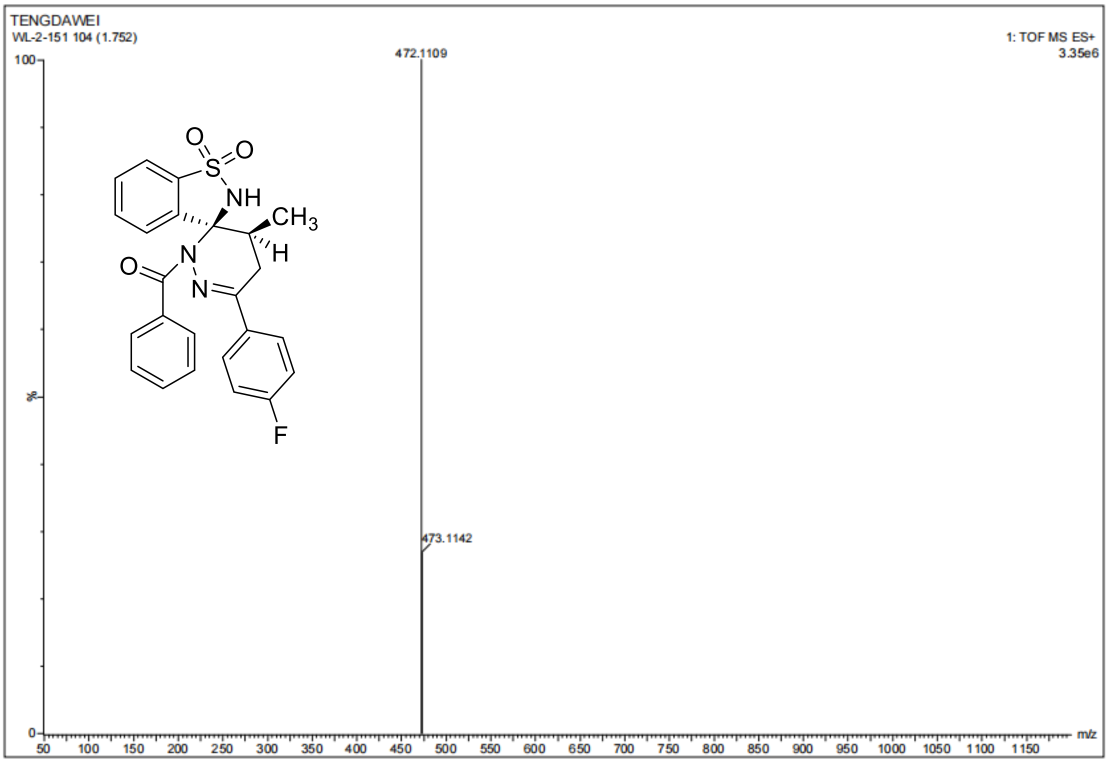

5) ((3*R**,4'*S**)-6'-(4-Chlorophenyl)-4'-methyl-1,1-dioxido-4',5'-dihydro-2*H*,2'*H*-spiro[benzo[*d*]isothiazole-3,3'-pyridazin]-2'-yl)(phenyl)methanone (**3ae**)

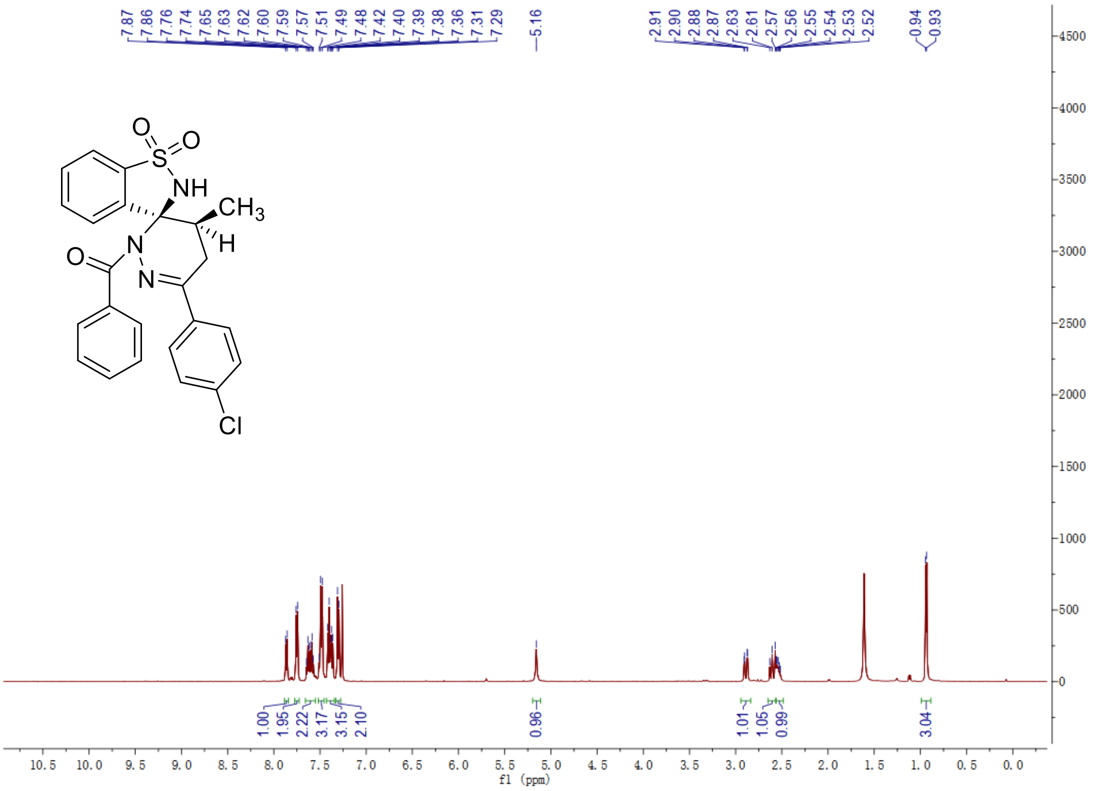

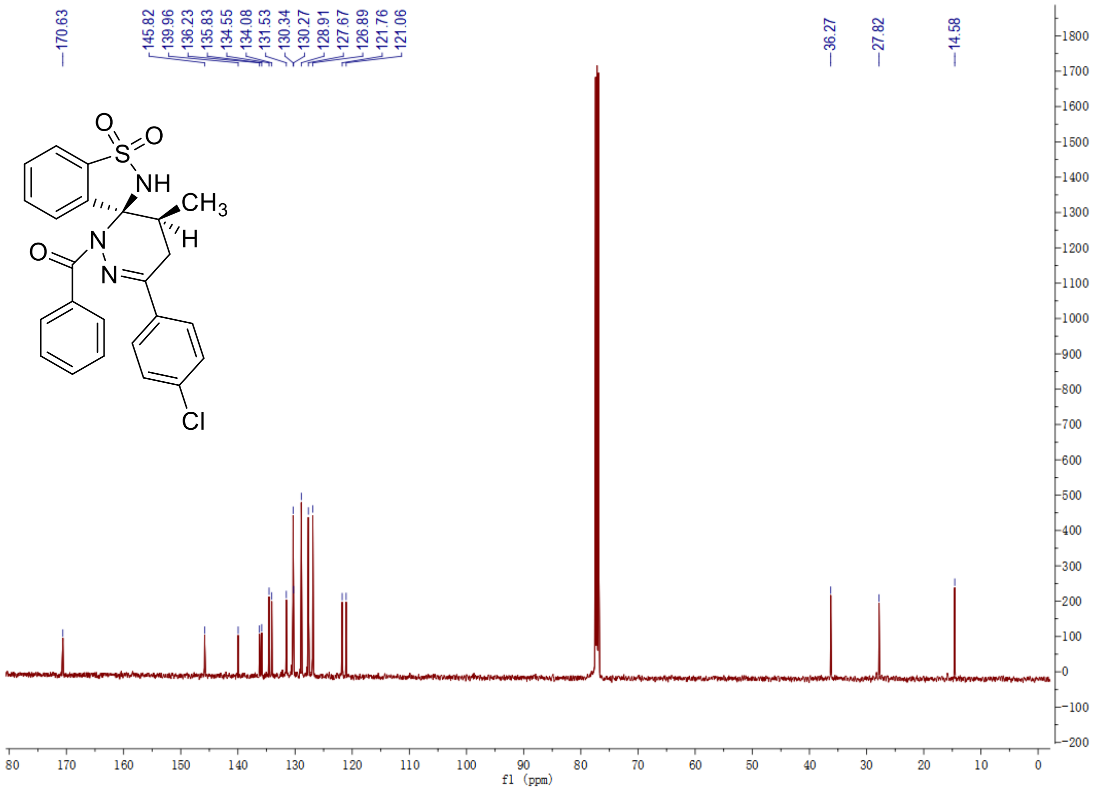
170.63
145.82
139.96
136.23
135.83
134.55
134.08
131.53
130.34
130.27
128.91
127.67
126.89
121.76
121.06
36.27
27.82
14.58
NH
CH3
H
N
N
O
Cl
f1 (ppm)

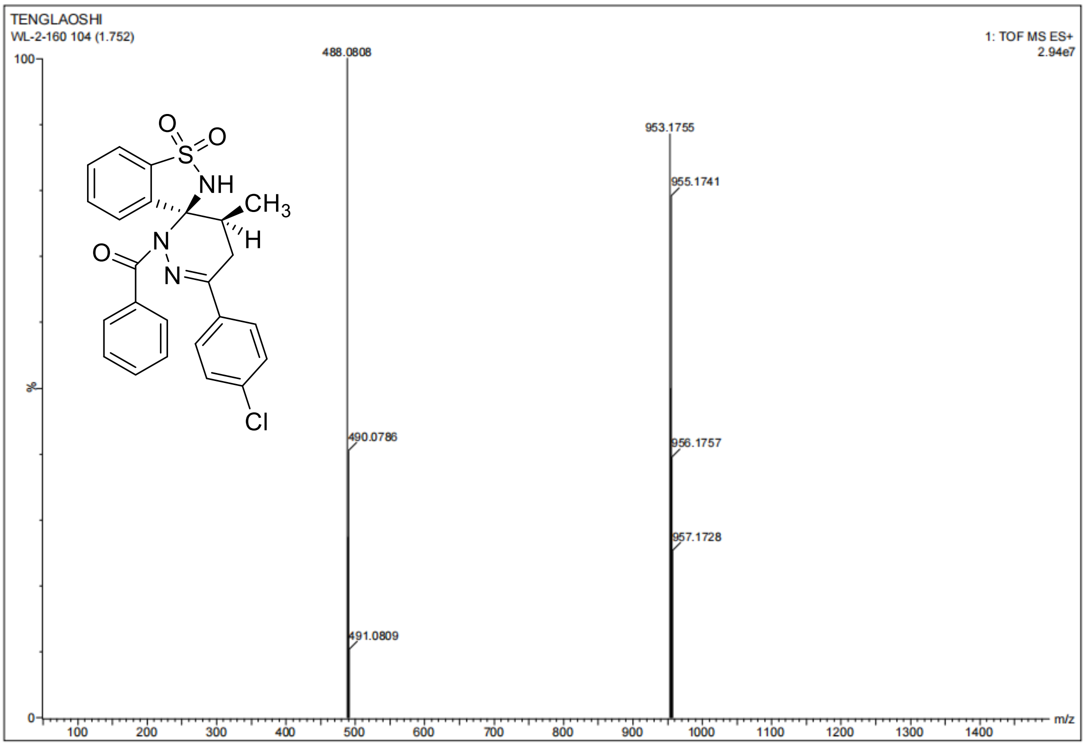
TENGLAOSHI
WL-2-160 104 (1.752)
1: TOF MS ES+
2.94e7
488.0808
490.0786
491.0809
953.1755
955.1741
956.1757
957.1728
%
m/z
NH
CH3
H
N
N
O
Cl

6) ((3*R**,4'*S**)-6'-(4-Bromophenyl)-4'-methyl-1,1-dioxido-4',5'-dihydro-2*H*,2'*H*-spiro[benzo[*d*]isothiazole-3,3'-pyridazin]-2'-yl)(phenyl)methanone (**3af**)

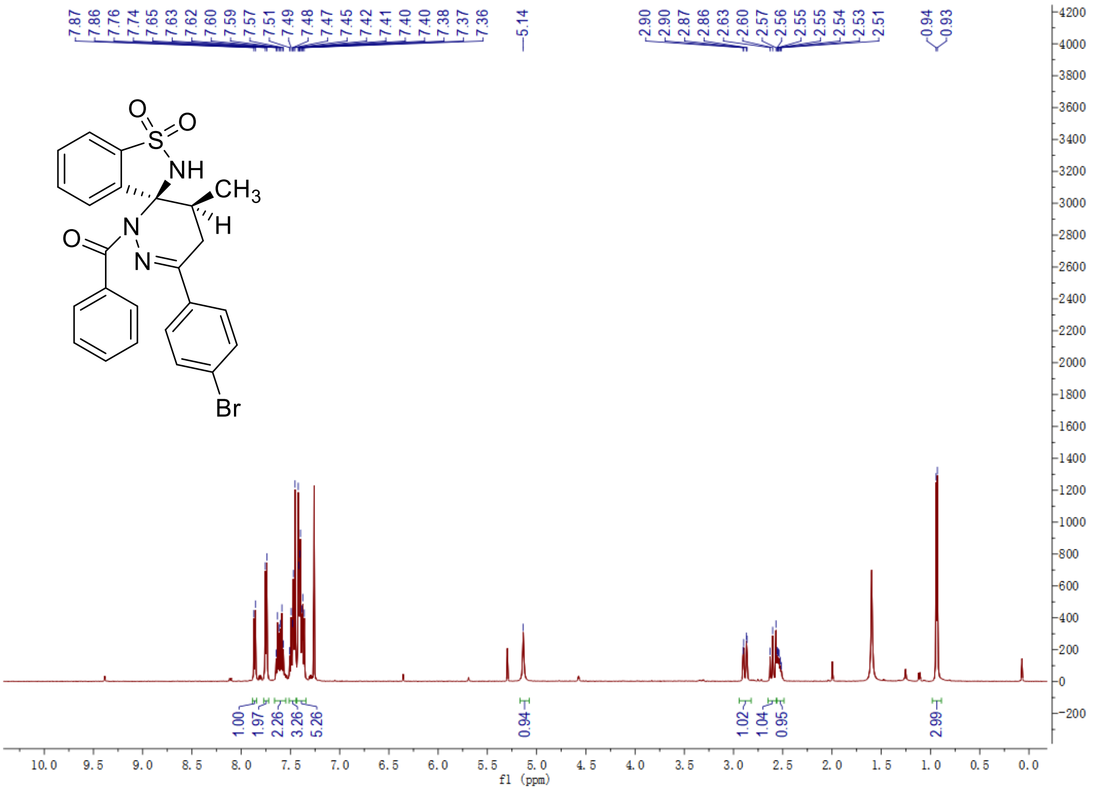

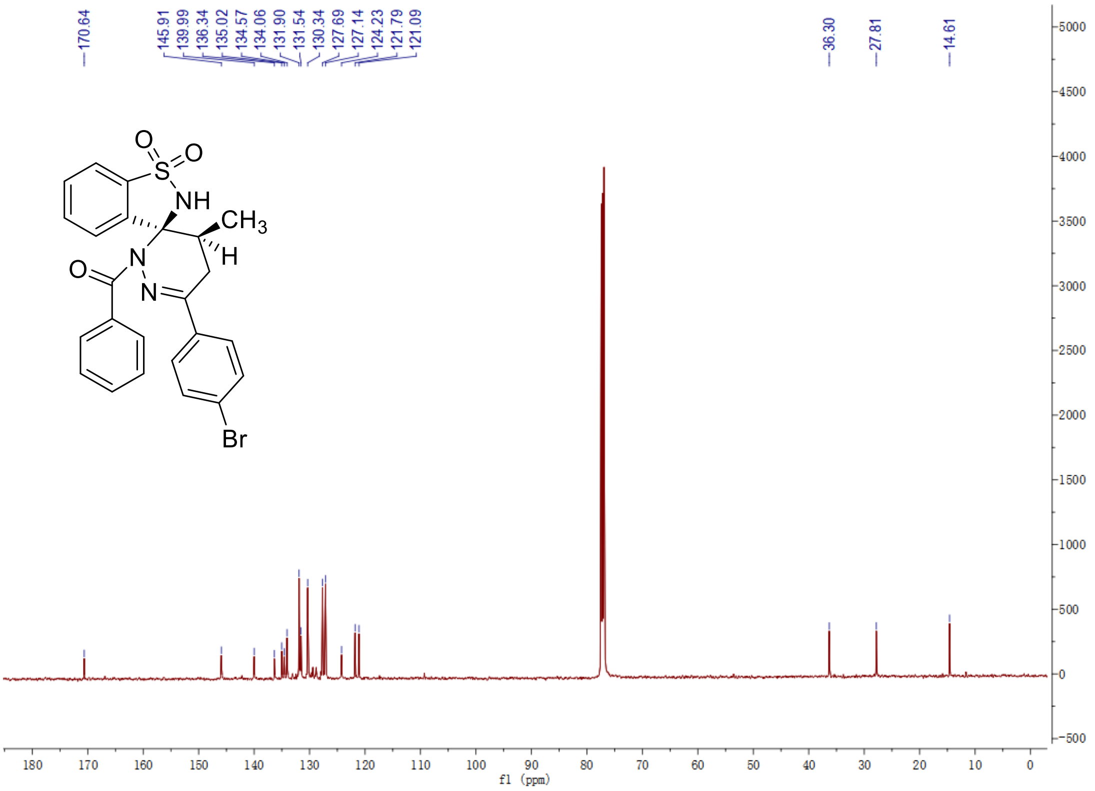

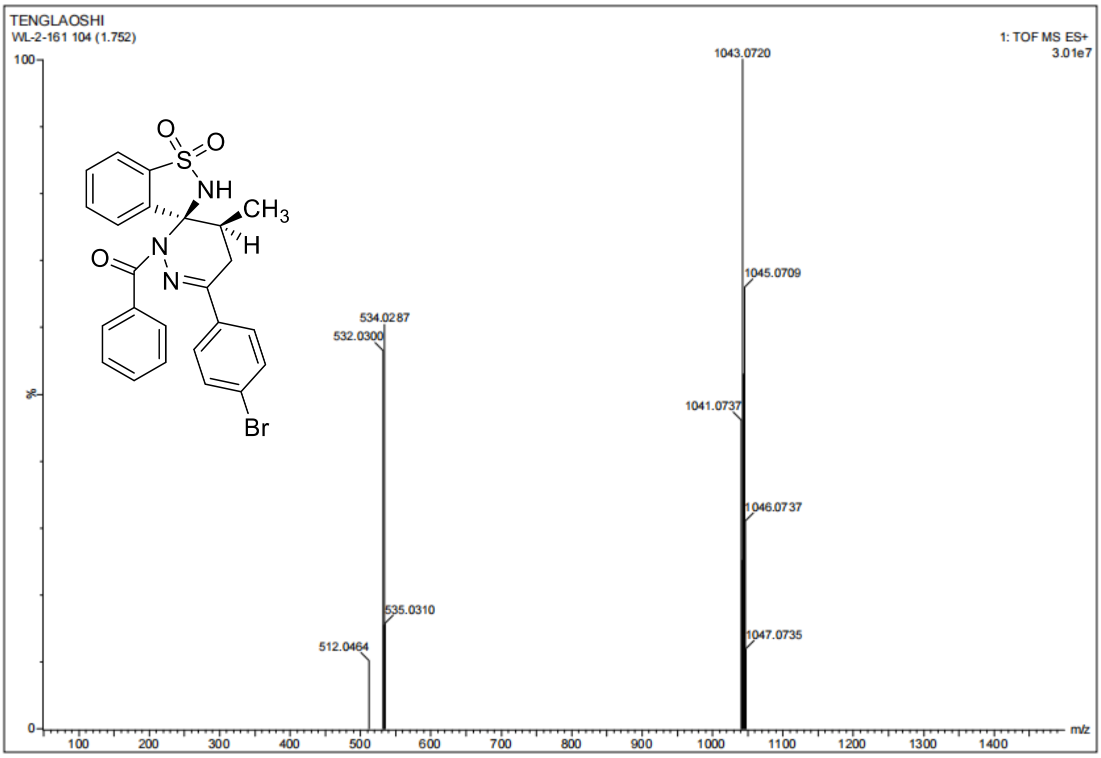

7) ((3*R**,4'*S**)-4'-Methyl-6'-(4-nitrophenyl)-1,1-dioxido-4',5'-dihydro-2*H*,2'*H*-spiro[benzo[*d*]isothiazole-3,3'-pyridazin]-2'-yl)(phenyl)methanone (**3ag**)

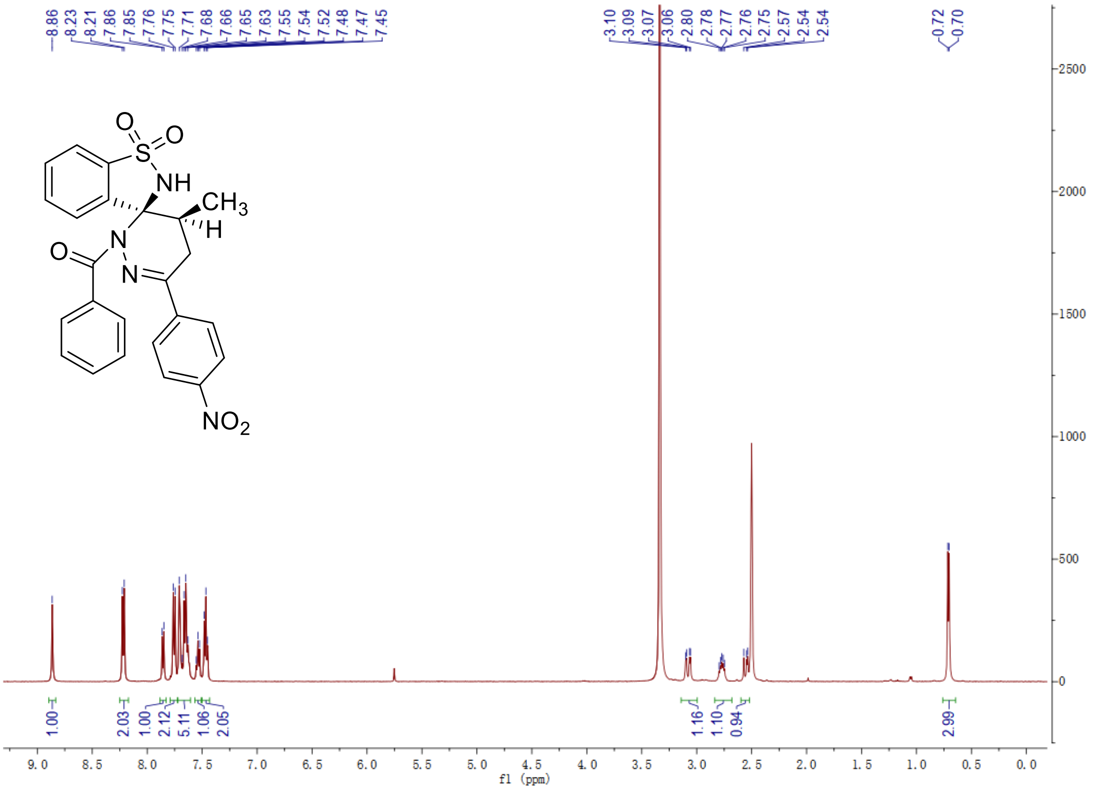

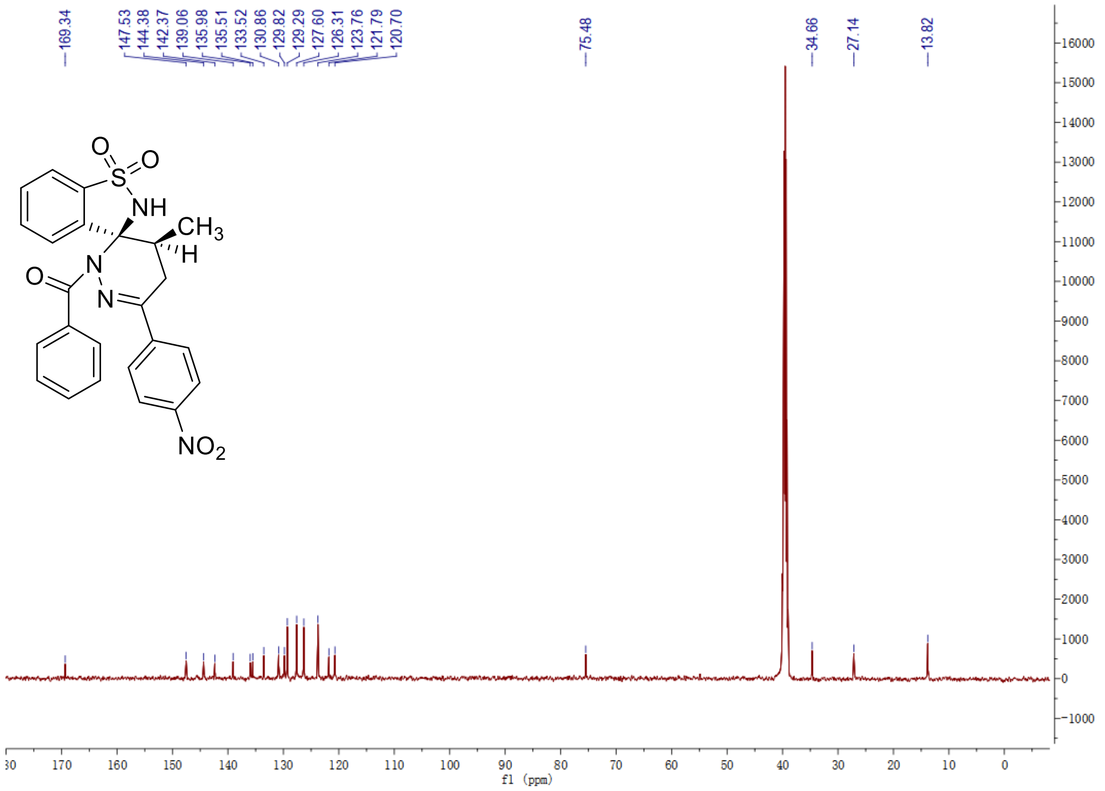
169.34
147.53
144.38
142.37
139.06
135.98
135.51
133.52
130.86
129.82
129.29
127.60
126.31
123.76
121.79
120.70
75.48
34.66
27.14
13.82
f1 (ppm)

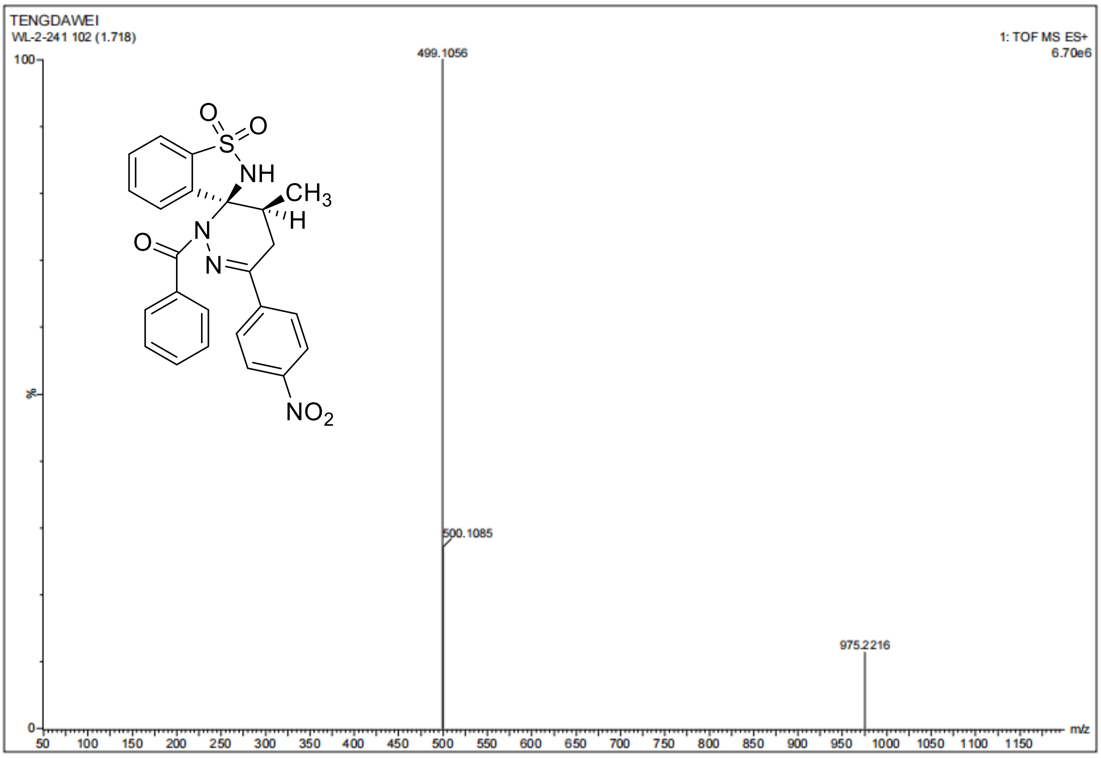
TENGDAWEI
WL-2-241 102 (1.718)
1: TOF MS ES+
6.70e6
499.1056
500.1085
975.2216
m/z

8) ((3*R**,4'*S**)-4'-Methyl-1,1-dioxido-6'-phenyl-4',5'-dihydro-2*H*,2'*H*-spiro[benzo[*d*]isothiazole-3,3'-pyridazin]-2'-yl)(*p*-tolyl)methanone (**3ah**)

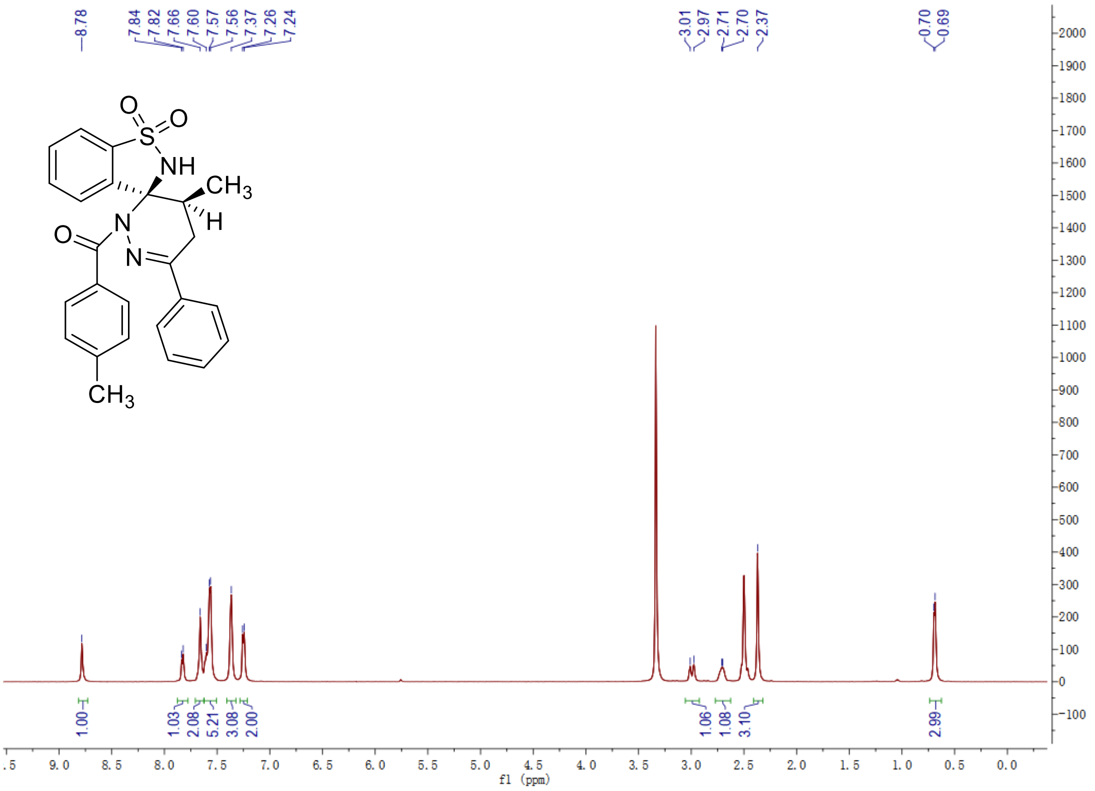

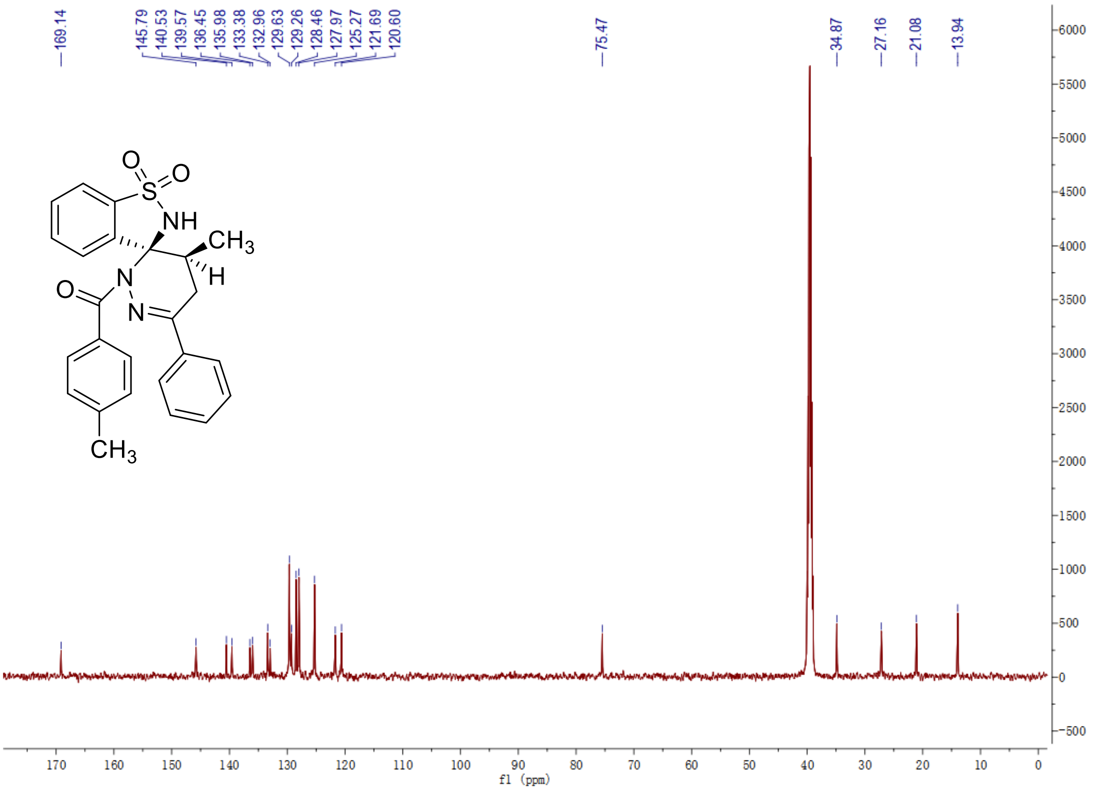

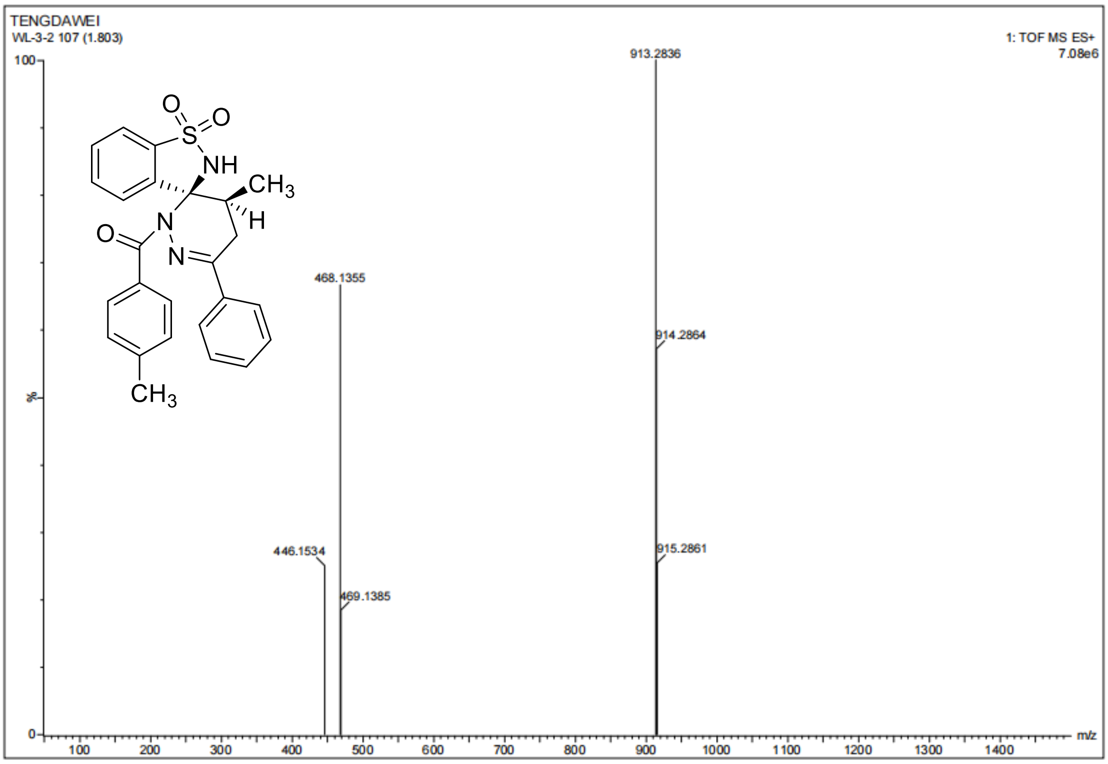

9) (4-Methoxyphenyl)((3*R**,4'*S**)-4'-methyl-1,1-dioxido-6'-phenyl-4',5'-dihydro-2*H*,2'*H*-spiro[benzo[*d*]isothiazole-3,3'-pyridazin]-2'-yl)methanone (**3ai**)

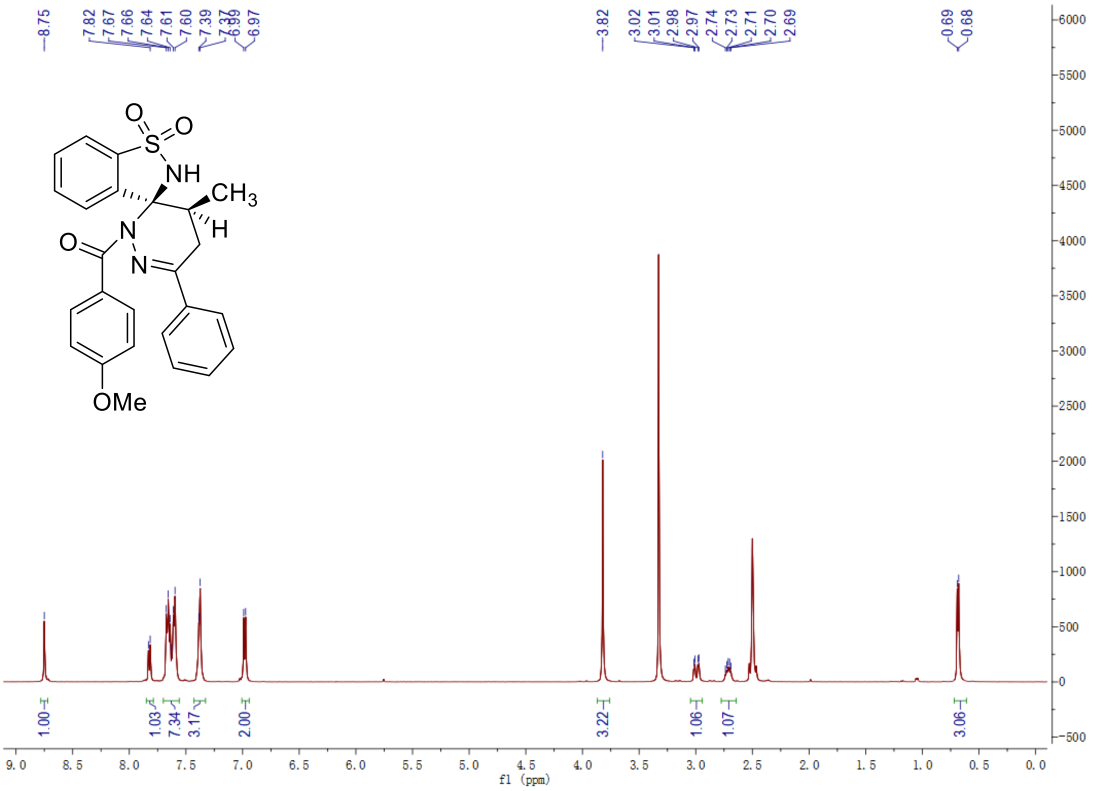

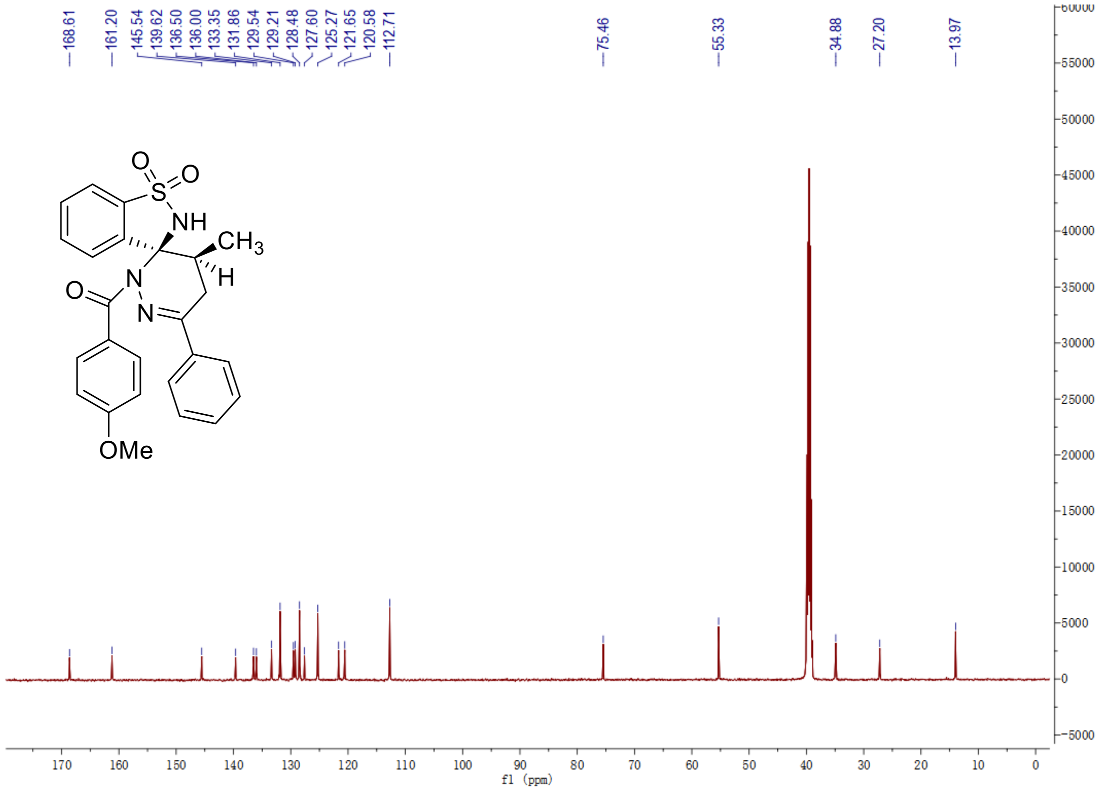
168.61
161.20
145.54
139.62
136.50
136.00
133.35
131.86
129.54
129.21
128.48
127.60
125.27
121.65
120.58
112.71
75.46
55.33
34.88
27.20
13.97
NH
CH3
H
O
N
N
OMe
f1 (ppm)

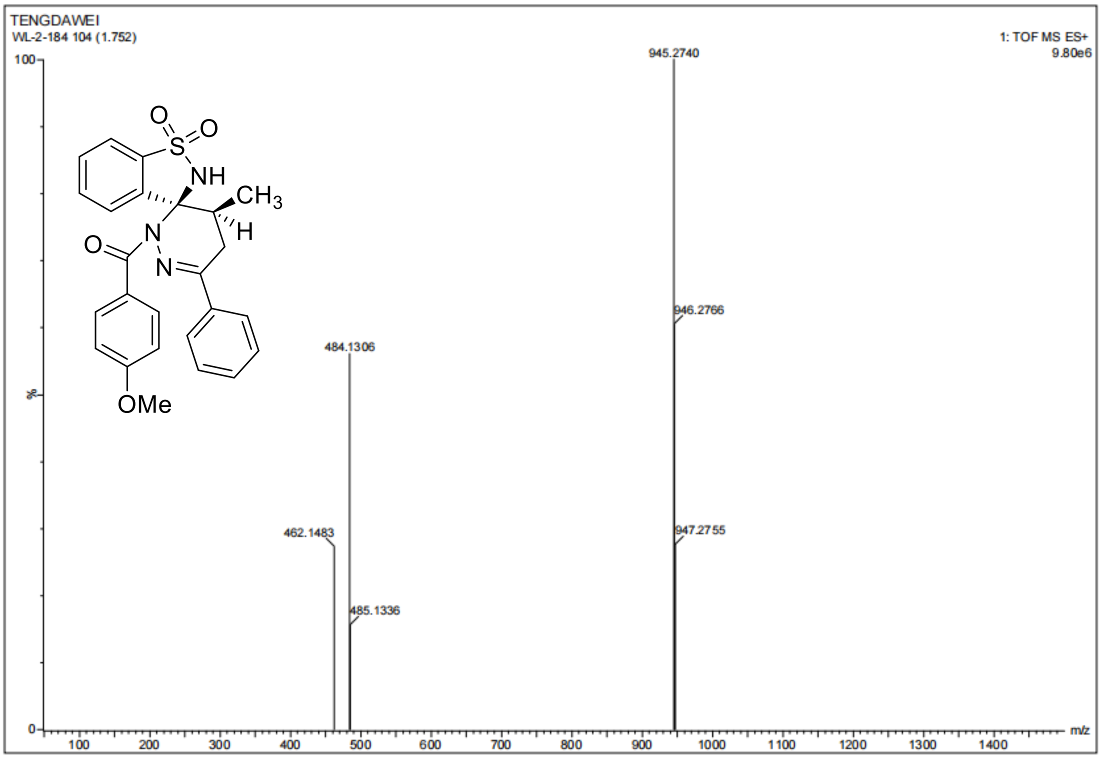
TENGDAWEI
WL-2-184 104 (1.752)
1: TOF MS ES+
9.80e6
945.2740
946.2766
484.1306
462.1483
947.2755
485.1336
%
m/z
OMe

10) (4-Fluorophenyl)((3*R**,4'*S**)-4'-methyl-1,1-dioxido-6'-phenyl-4',5'-dihydro-2*H*,2'*H*-spiro[benzo[*d*]isothiazole-3,3'-pyridazin]-2'-yl)methanone (**3aj**)

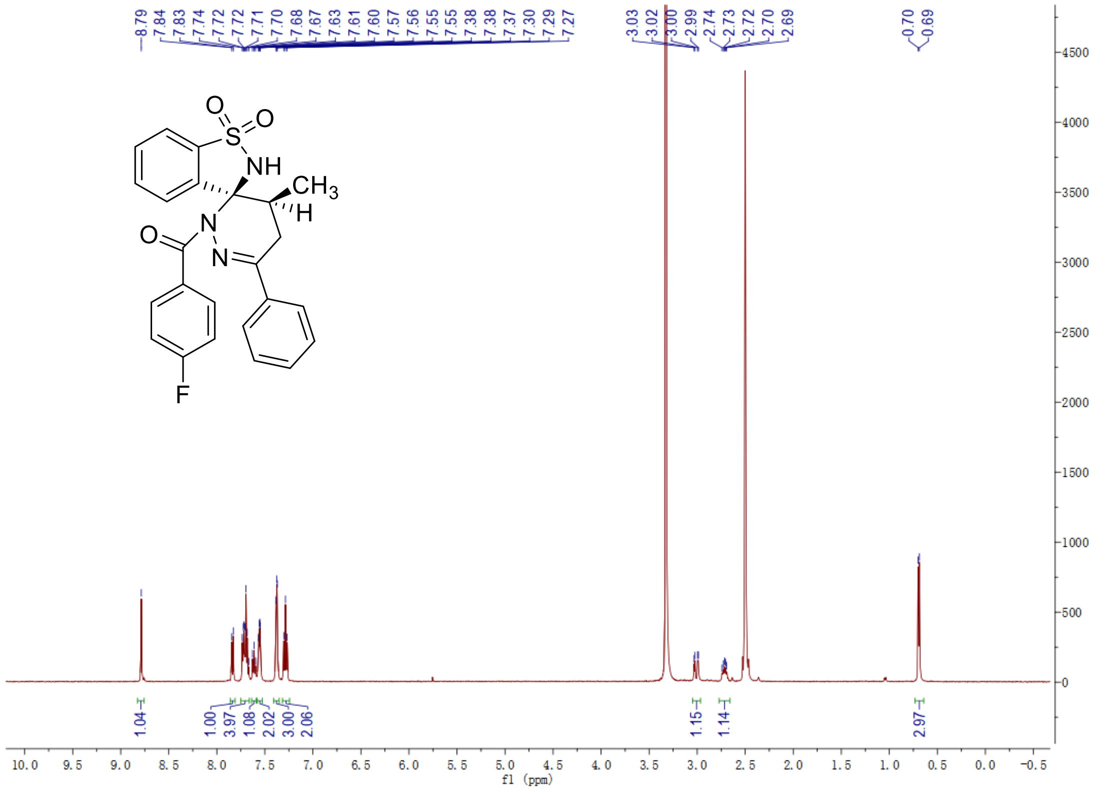

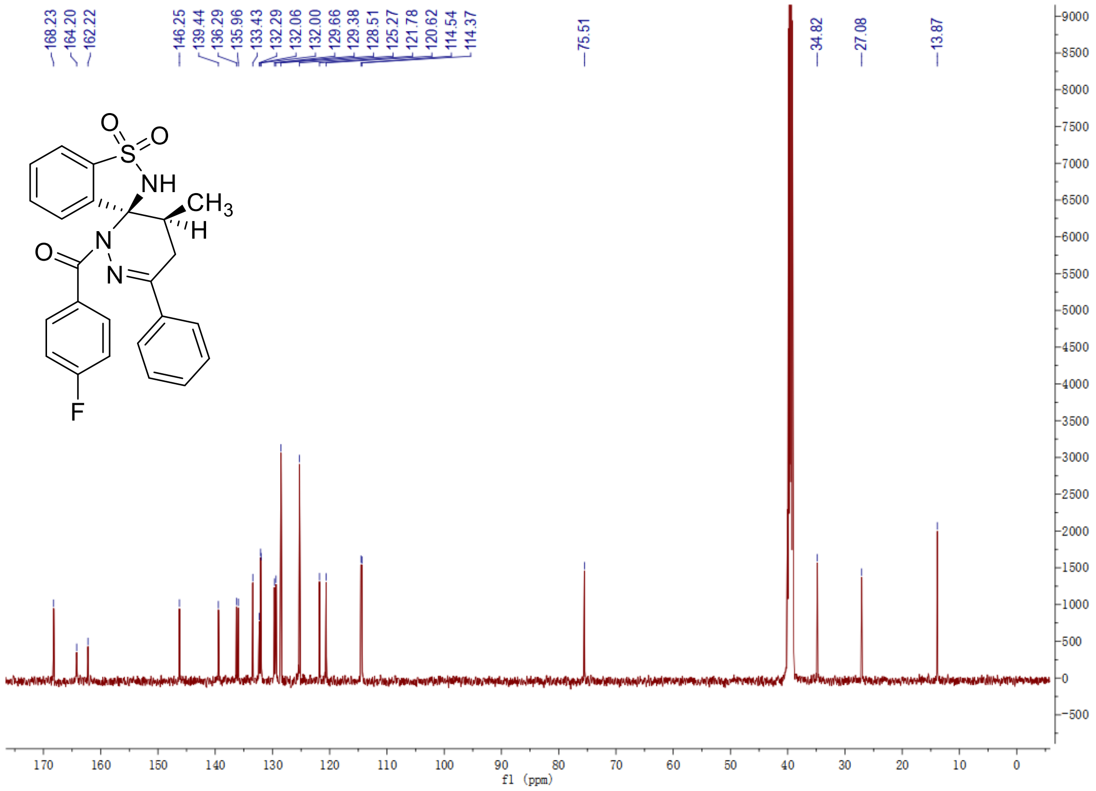

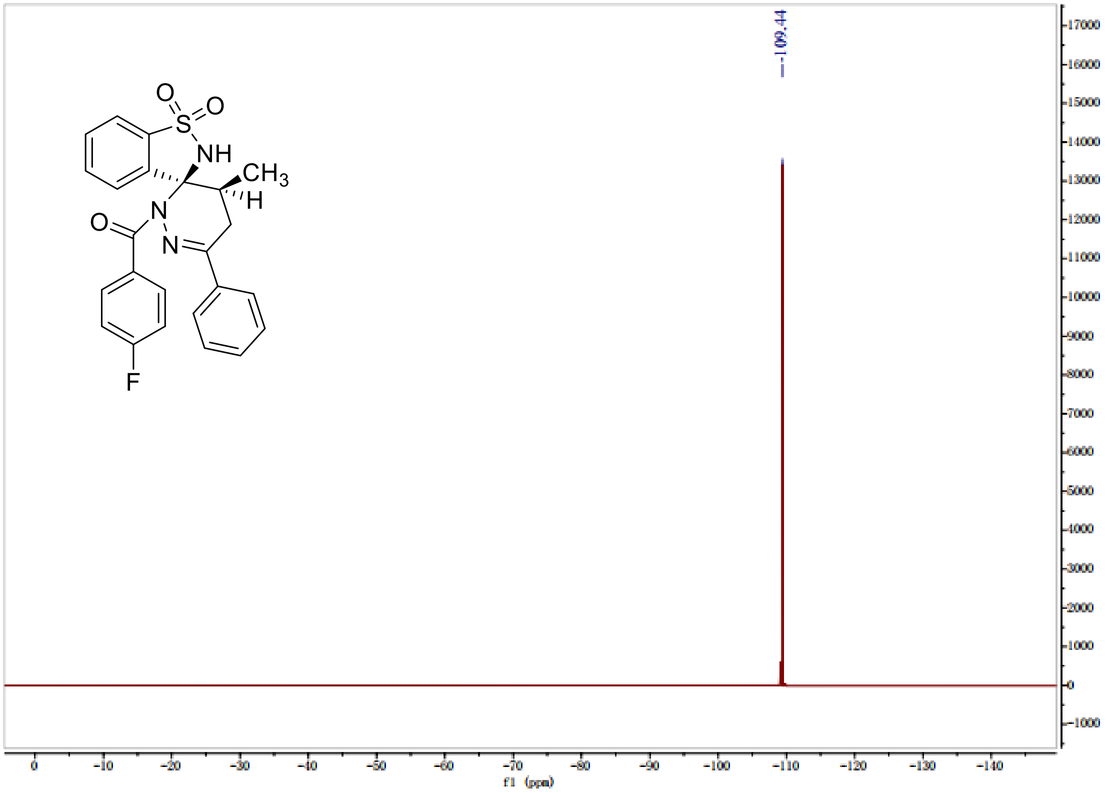

-109.44
f1 (ppm)

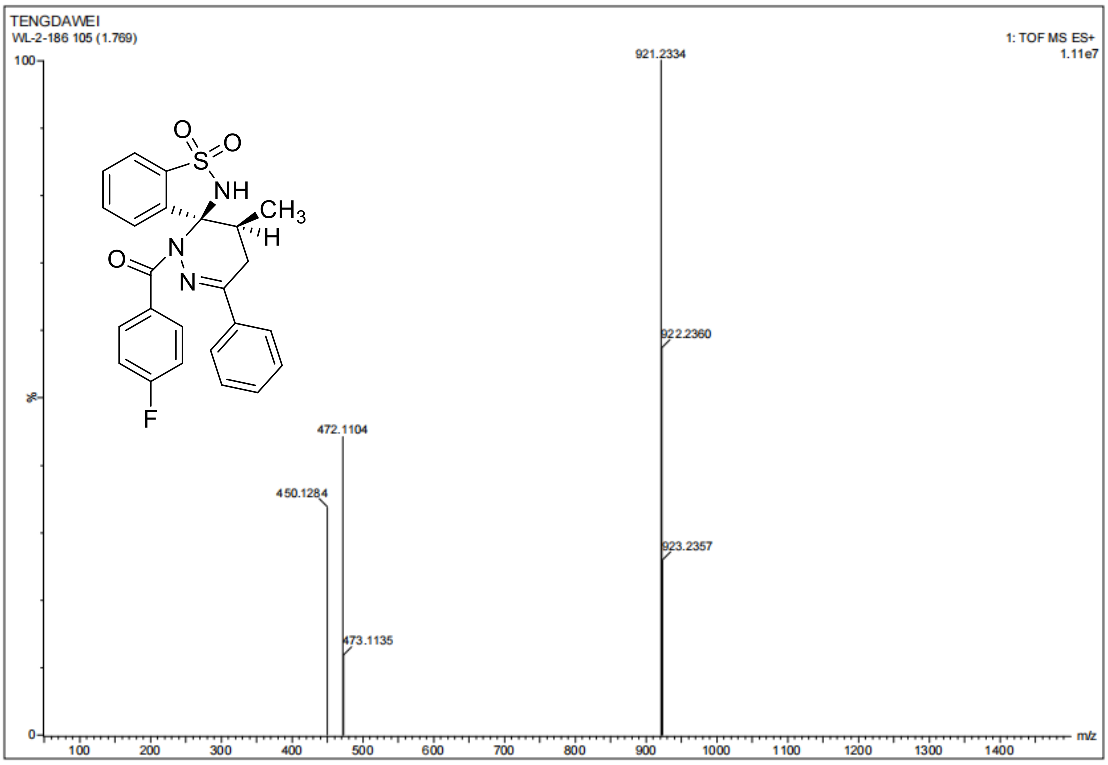

TENGDAWEI
WL-2-186 105 (1.769)
1: TOF MS ES+
1.11e7
921.2334
922.2360
923.2357
472.1104
450.1284
473.1135
%
m/z

11) (4-Chlorophenyl)((3*R**,4'*S**)-4'-methyl-1,1-dioxido-6'-phenyl-4',5'-dihydro-2*H*,2'*H*-spiro[benzo[*d*]isothiazole-3,3'-pyridazin]-2'-yl)methanone (**3ak**)

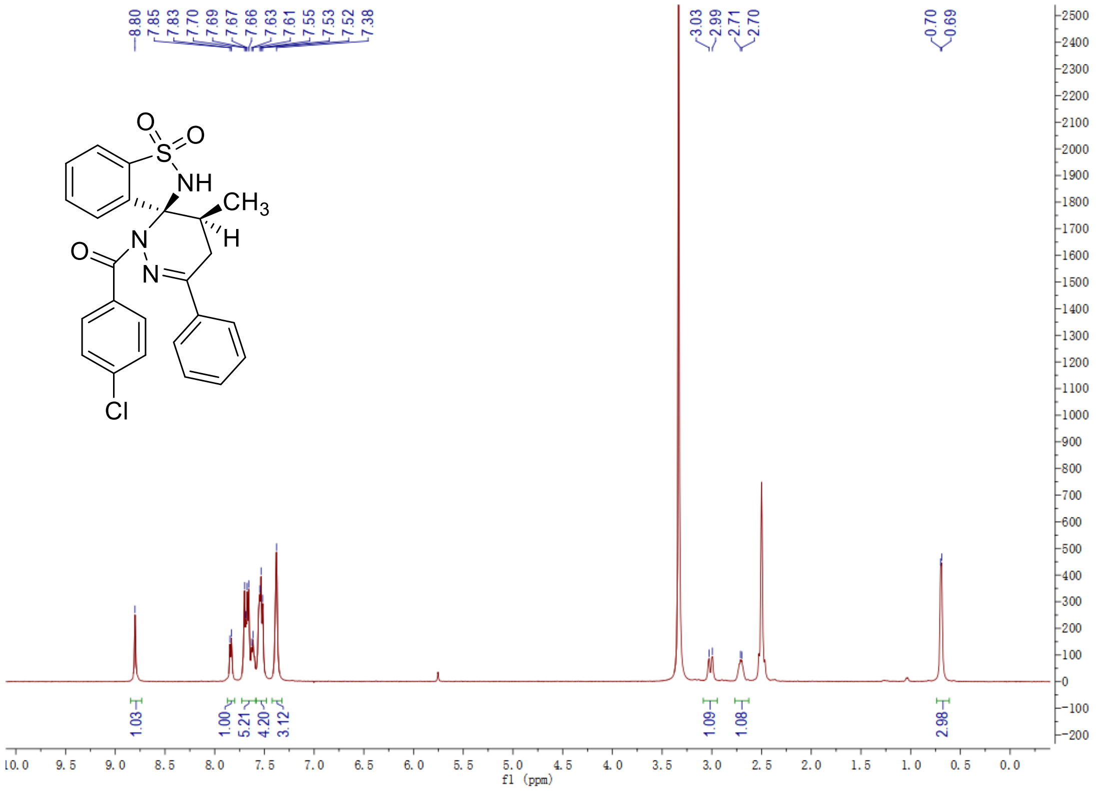

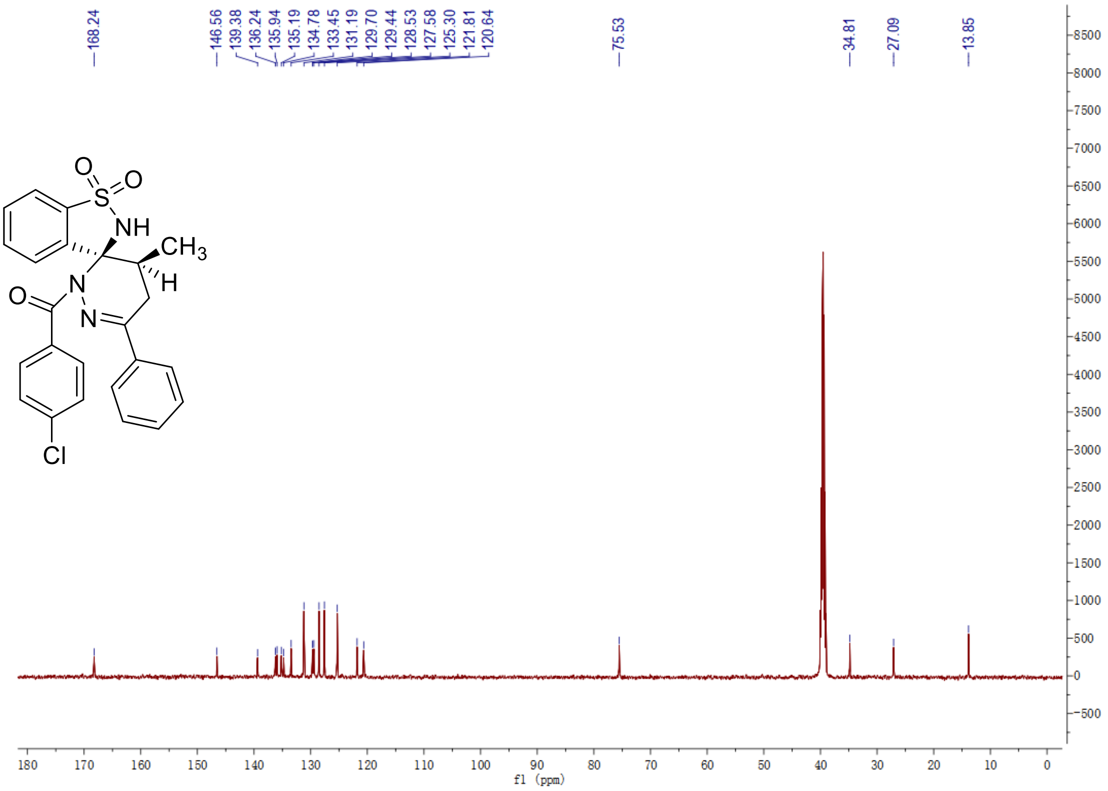

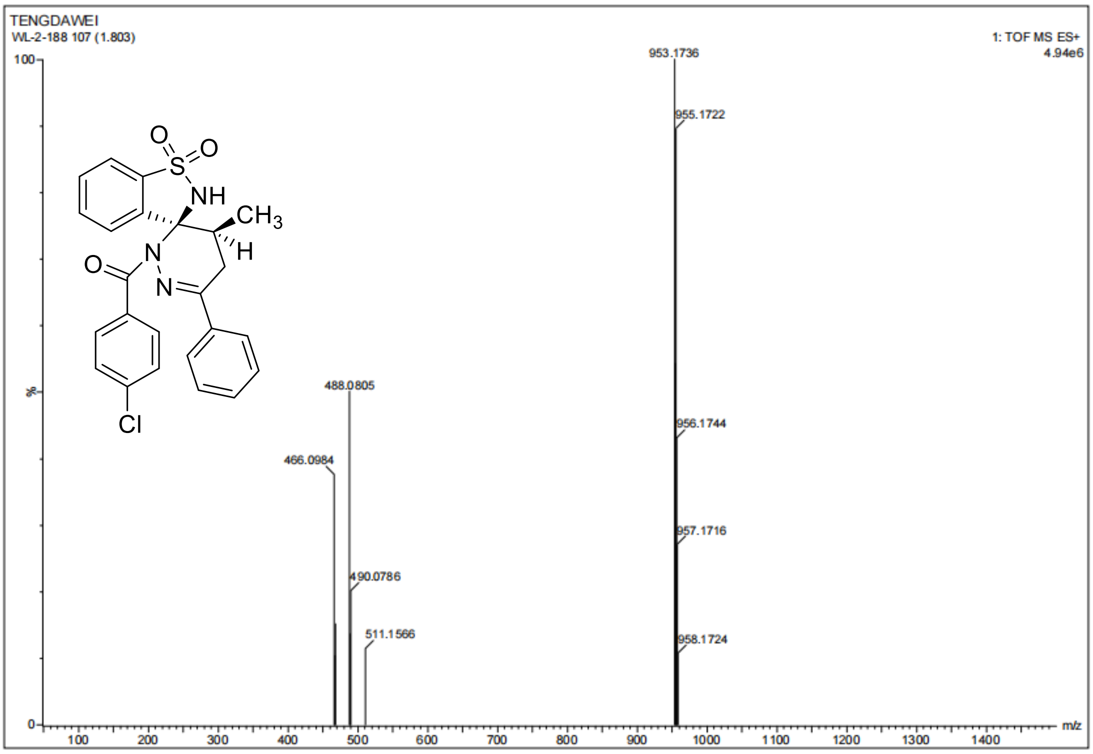

12) (4-Bromophenyl)((3*R**,4'*S**)-4'-methyl-1,1-dioxido-6'-phenyl-4',5'-dihydro-2*H*,2'*H*-spiro[benzo[*d*]isothiazole-3,3'-pyridazin]-2'-yl)methanone (**3al**)

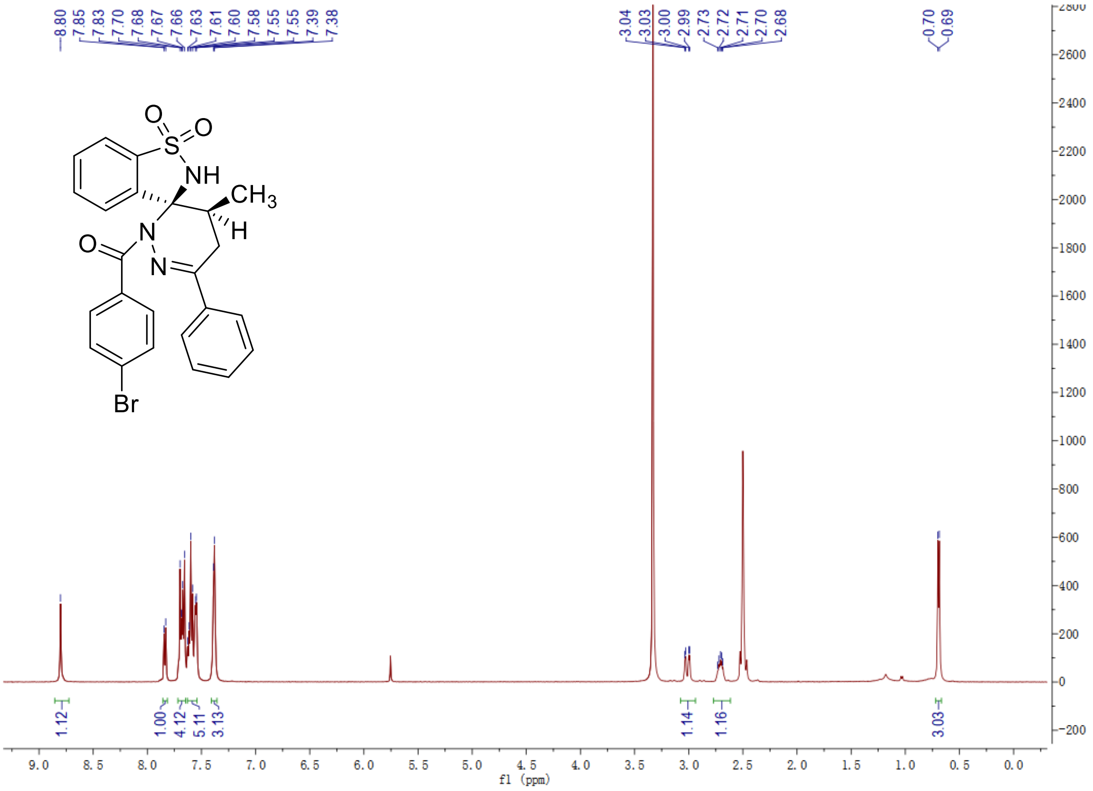

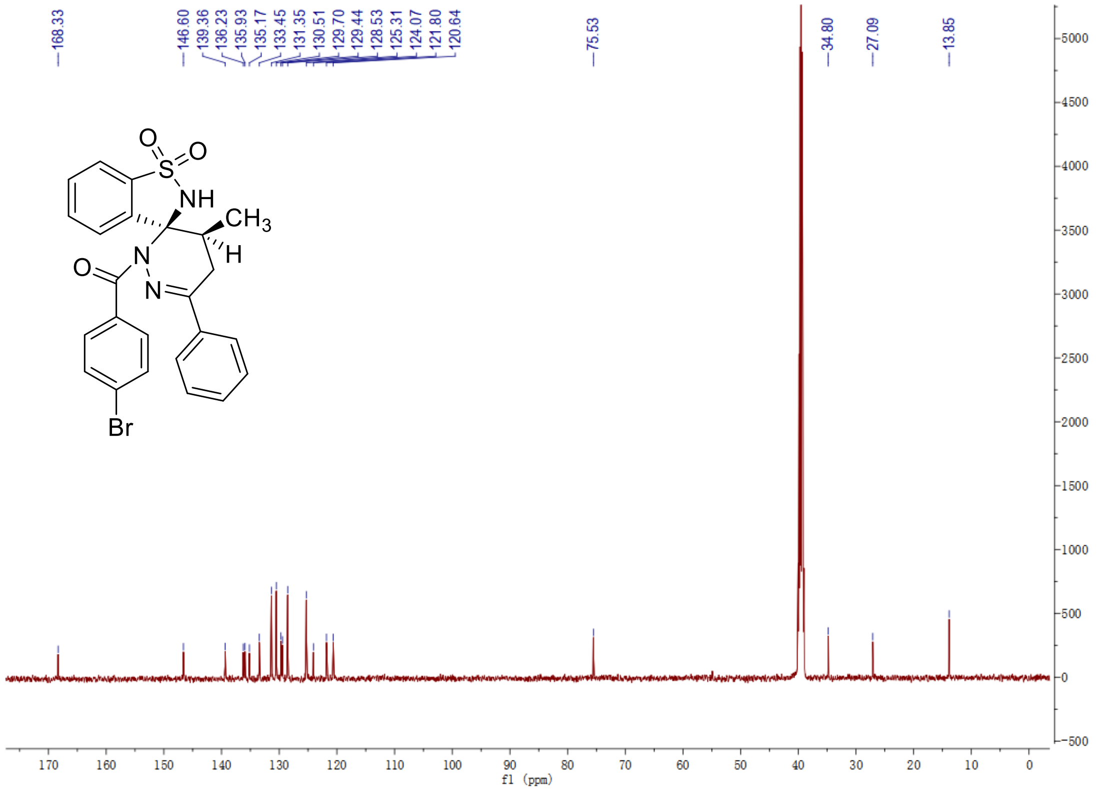
168.33
146.60
139.36
136.23
135.93
135.17
133.45
131.35
130.51
129.70
129.44
128.53
125.31
124.07
121.80
120.64
75.53
34.80
27.09
13.85
O
O
S
NH
CH3
H
N
N
O
Br
f1 (ppm)

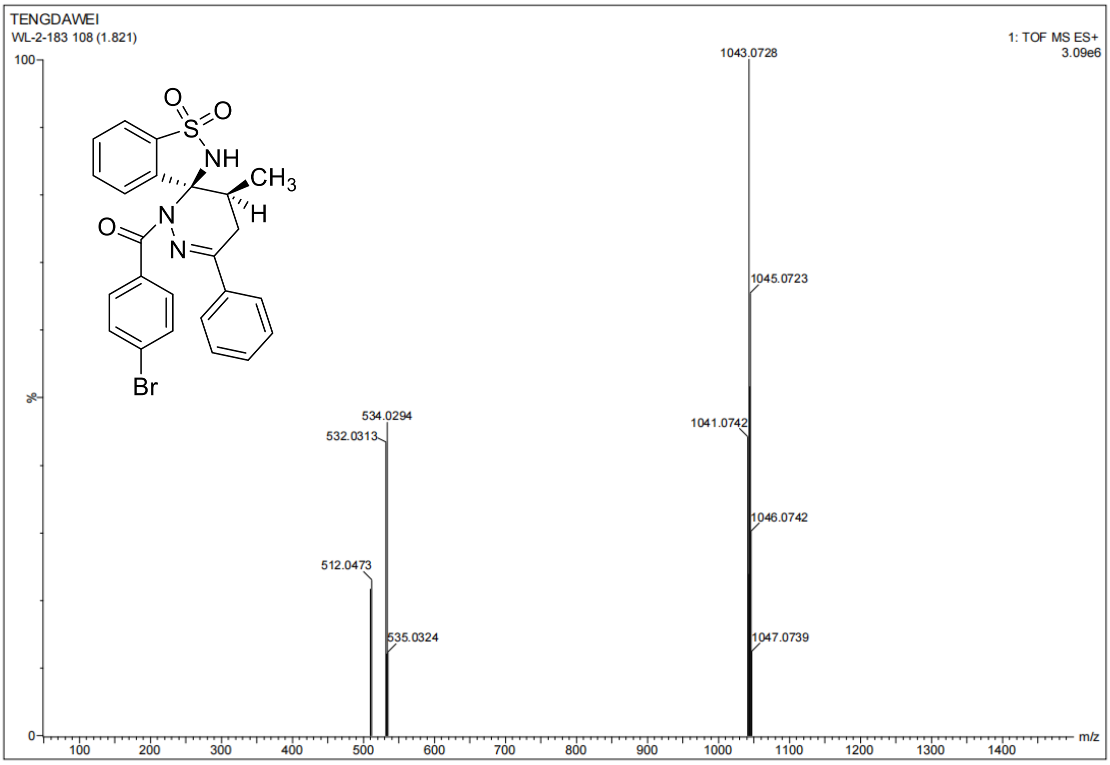
TENGDAWEI
WL-2-183 108 (1.821)
1: TOF MS ES+
3.09e6
1043.0728
1045.0723
1041.0742
1046.0742
1047.0739
534.0294
532.0313
512.0473
535.0324
%
m/z

13) ((3*R**,4'*S**)-6'-(3-Chloro-4-methoxyphenyl)-4'-methyl-1,1-dioxido-4',5'-dihydro-2*H*,2'*H*-spiro[benzo[*d*]isothiazole-3,3'-pyridazin]-2'-yl)(phenyl)methanone (**3am**)

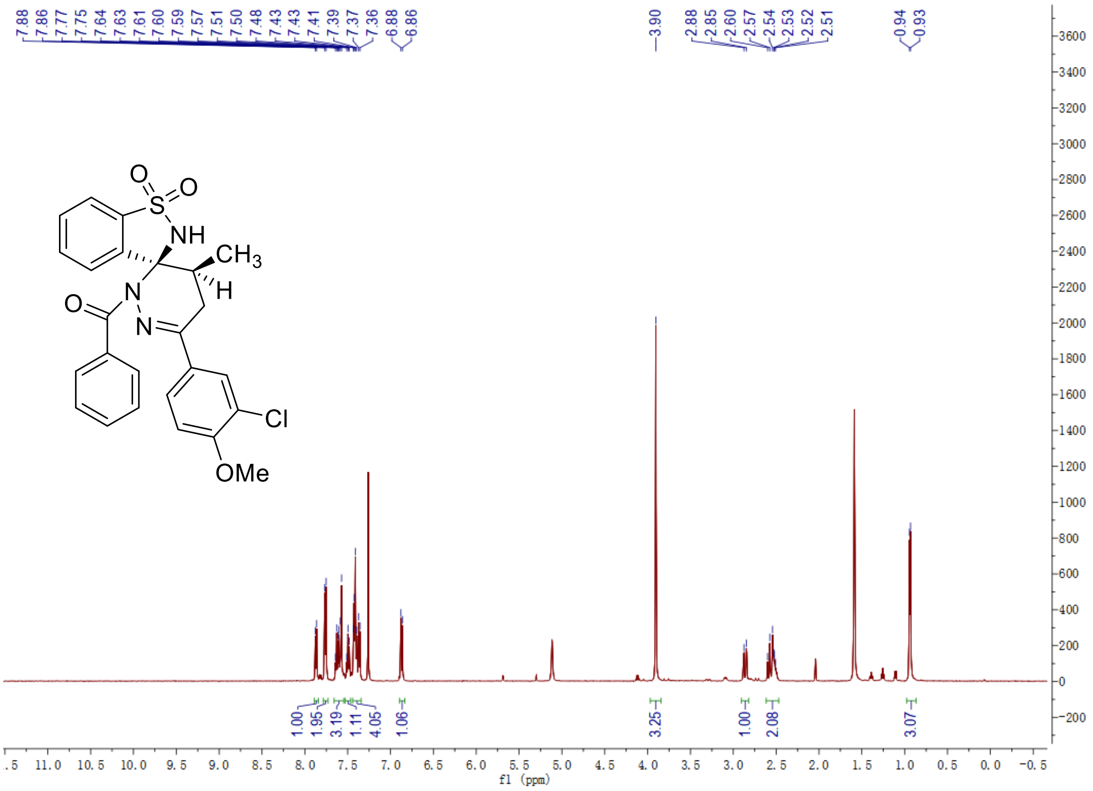

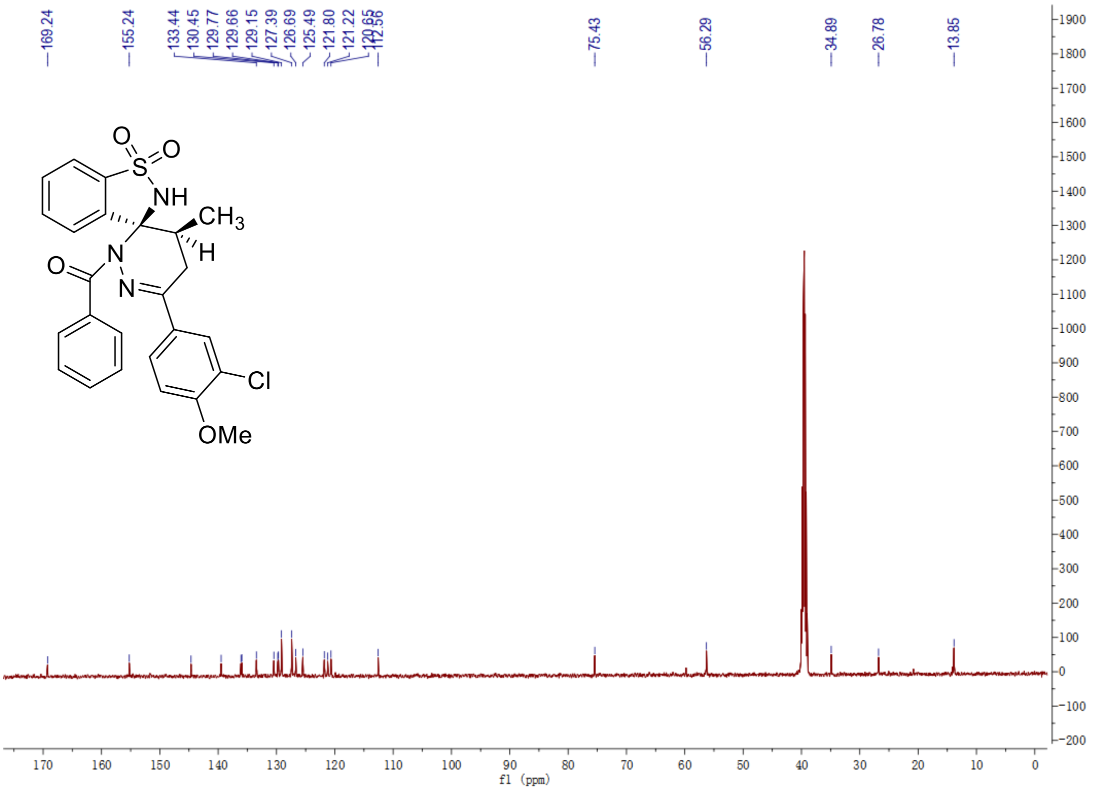

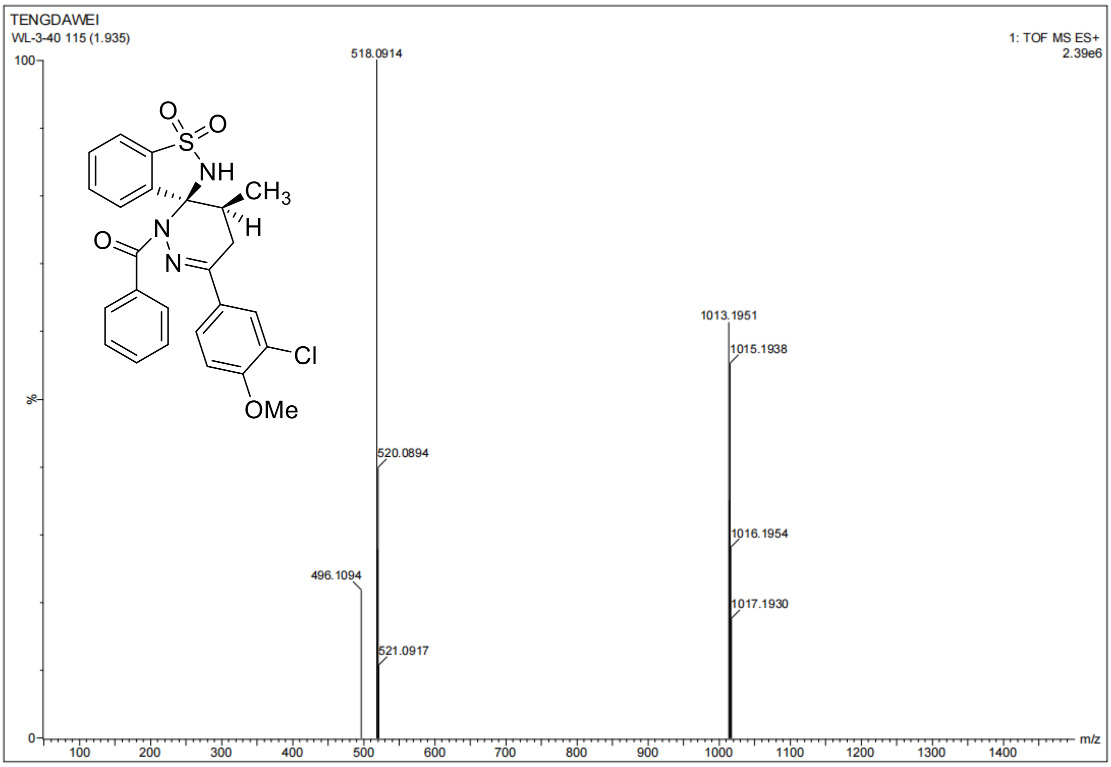

14) (*R**)-(4',4'-Dimethyl-1,1-dioxido-6'-phenyl-4',5'-dihydro-2*H*,2'*H*-spiro[benzo[*d*]isothiazole-3,3'-pyridazin]-2'-yl)(phenyl)methanone (**3ba**)

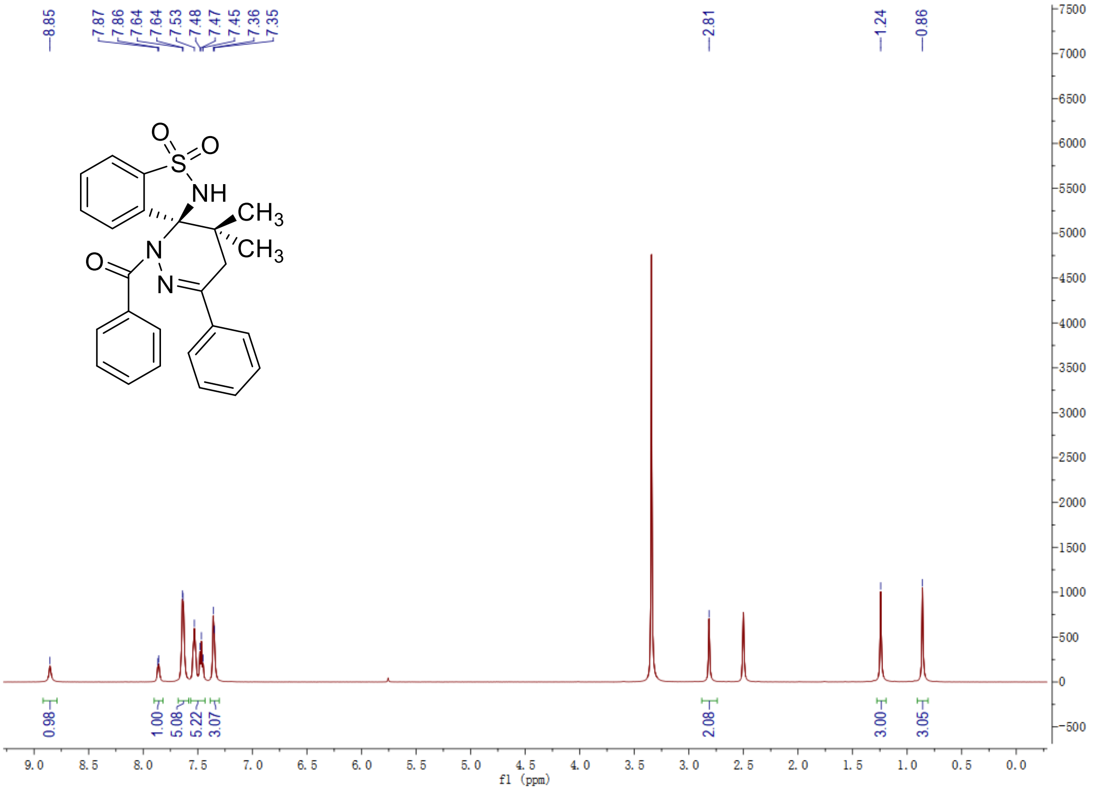

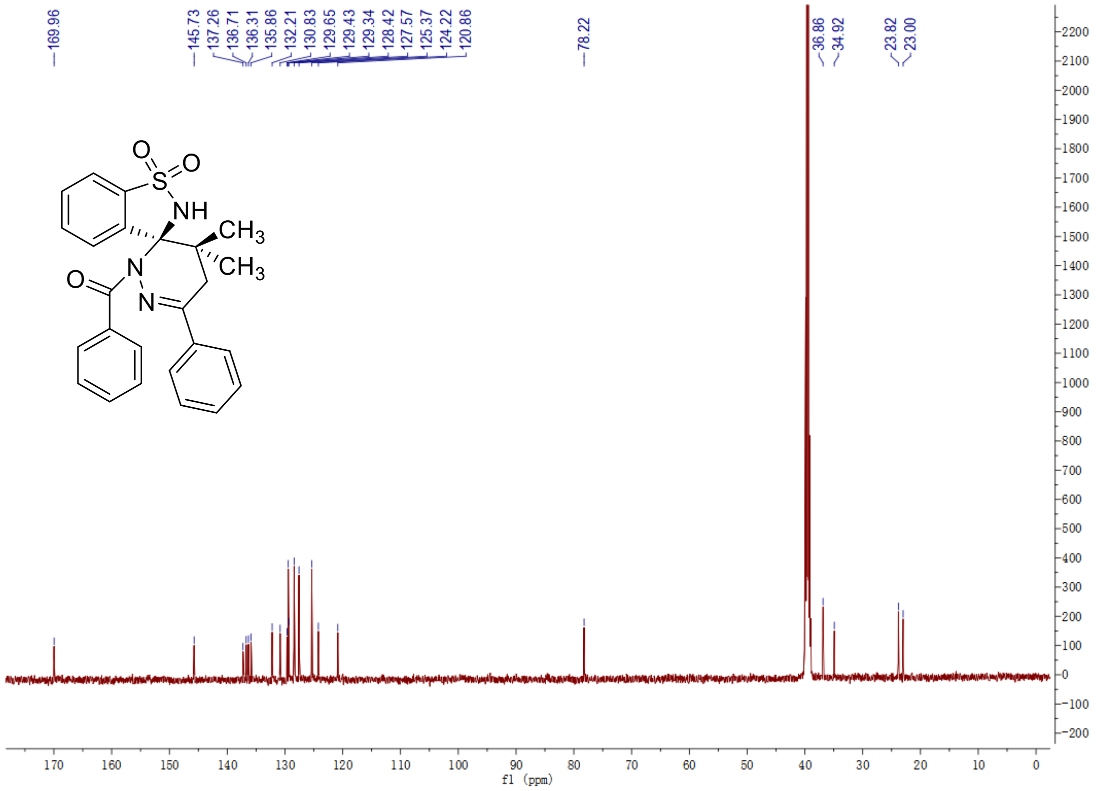
169.96
145.73
137.26
136.71
136.31
135.86
132.21
130.83
129.65
129.43
129.34
128.42
127.57
125.37
124.22
120.86
78.22
36.86
34.92
23.82
23.00
f1 (ppm)

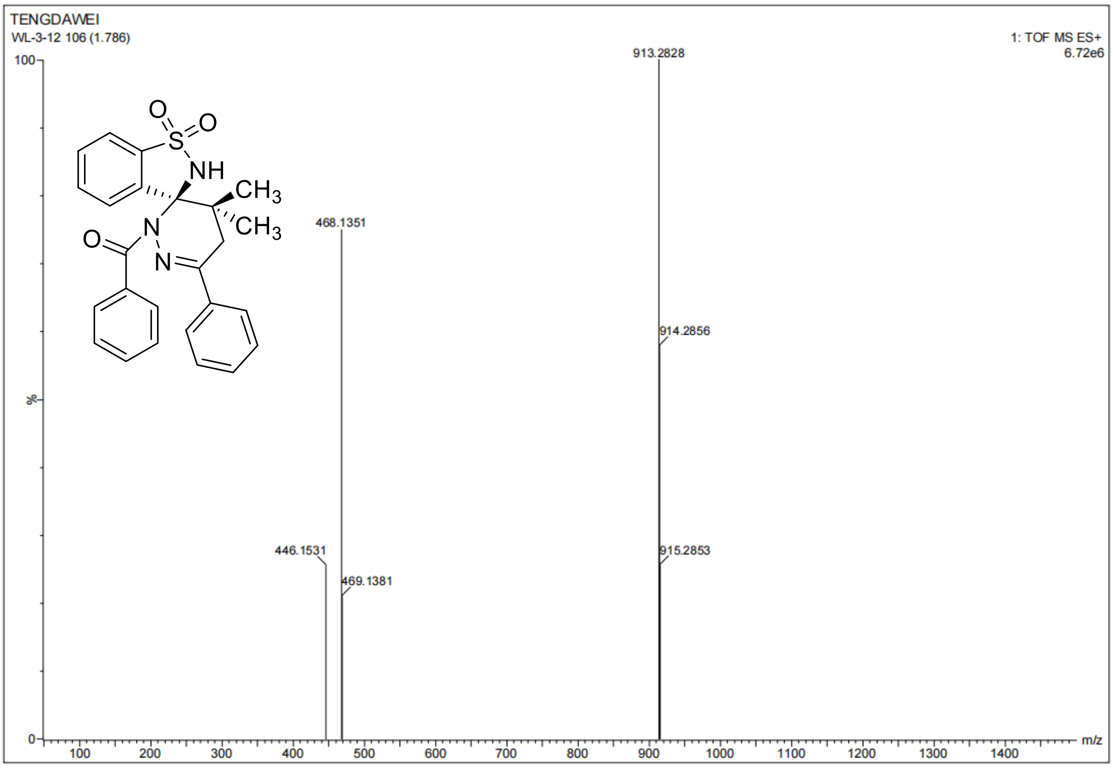
TENGDAWEI
WL-3-12 106 (1.786)
1: TOF MS ES+
6.72e6
913.2828
468.1351
914.2856
446.1531
915.2853
469.1381
%
m/z

15) ((3*R**,4'*S**)-1,1-Dioxido-6'-phenyl-4'-propyl-4',5'-dihydro-2*H*,2'*H*-spiro[benzo[*d*]isothiazole-3,3'-pyridazin]-2'-yl)(phenyl)methanone (**3ca**)

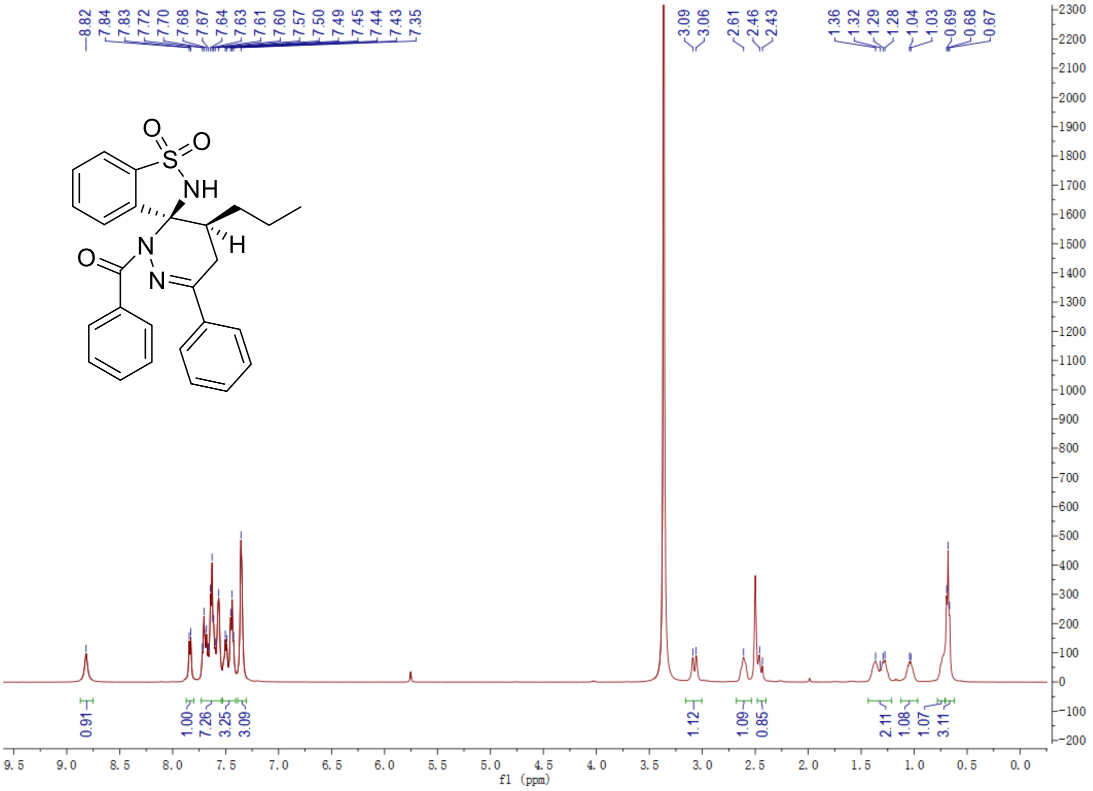

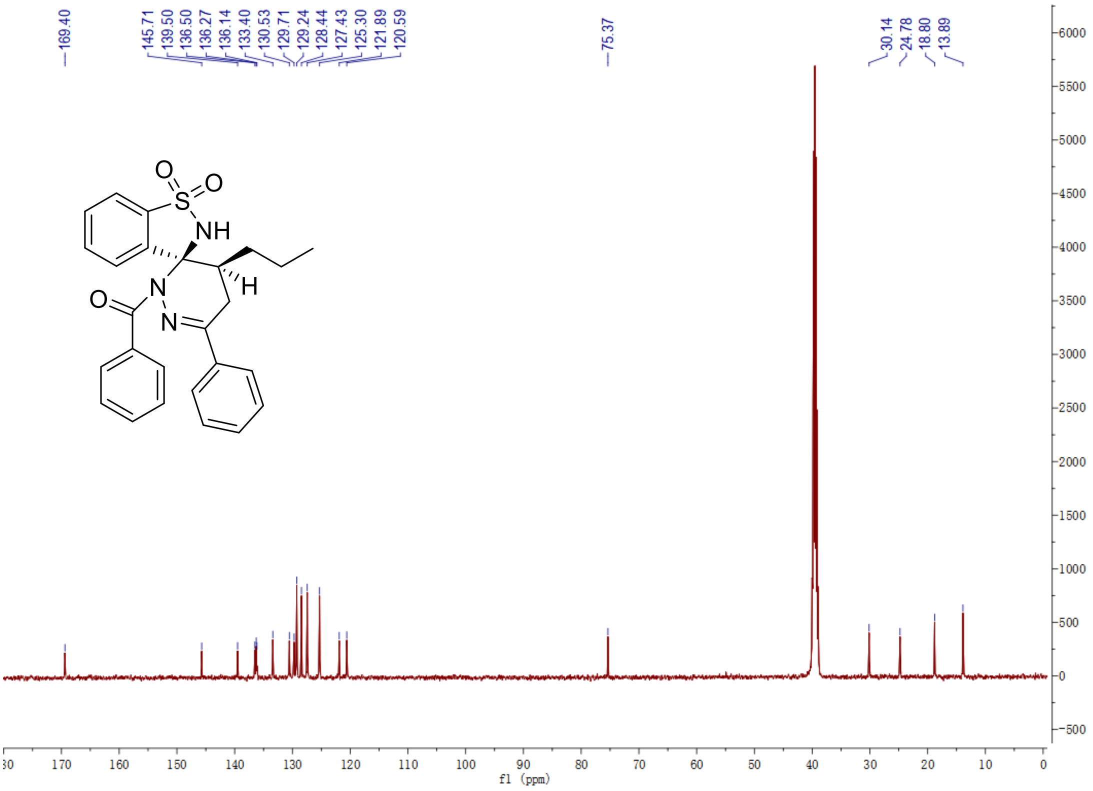

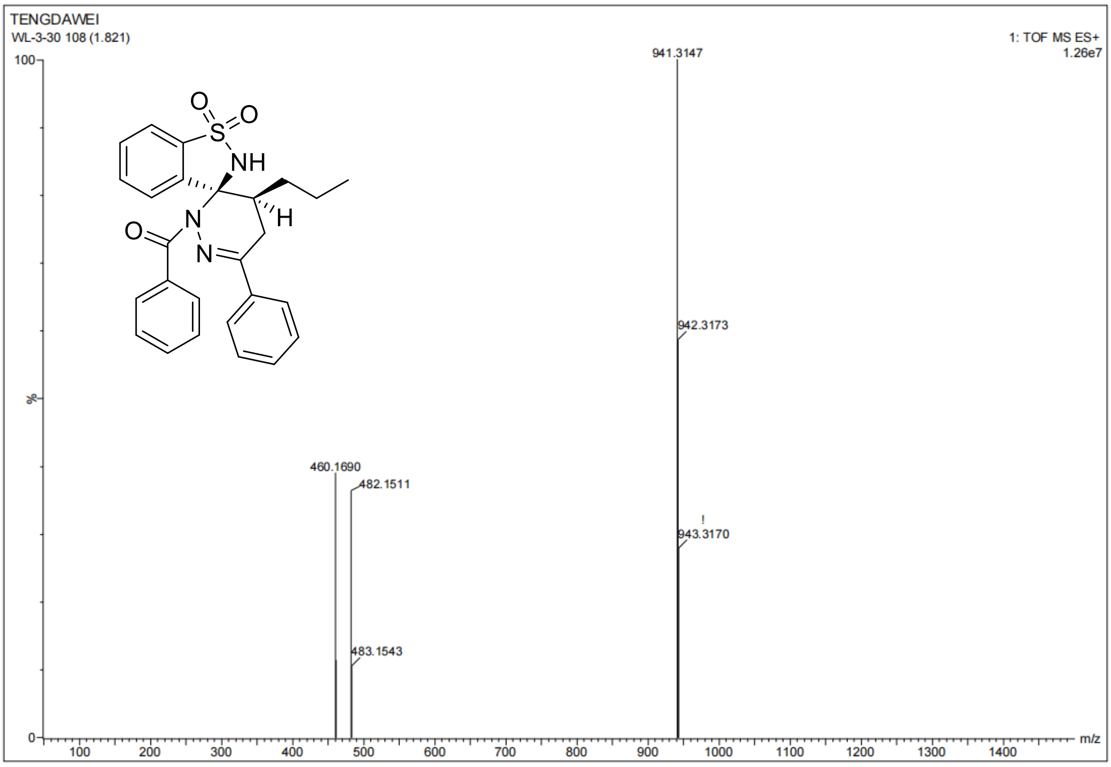

16) (*R**)-3-Methyl-3-(2-oxo-2-phenylethyl)-2,3-dihydro-4*H*-benzo[*e*][1,2]thiazin-4-one 1,1-dioxide (**4aa**)

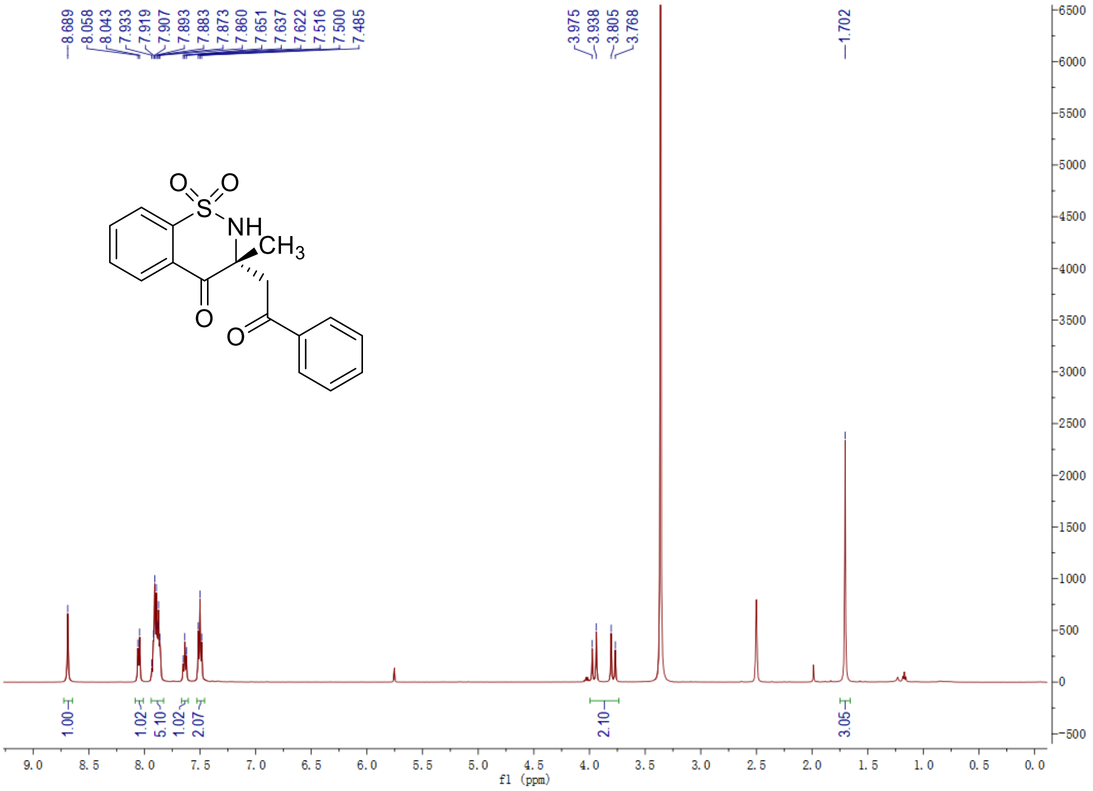

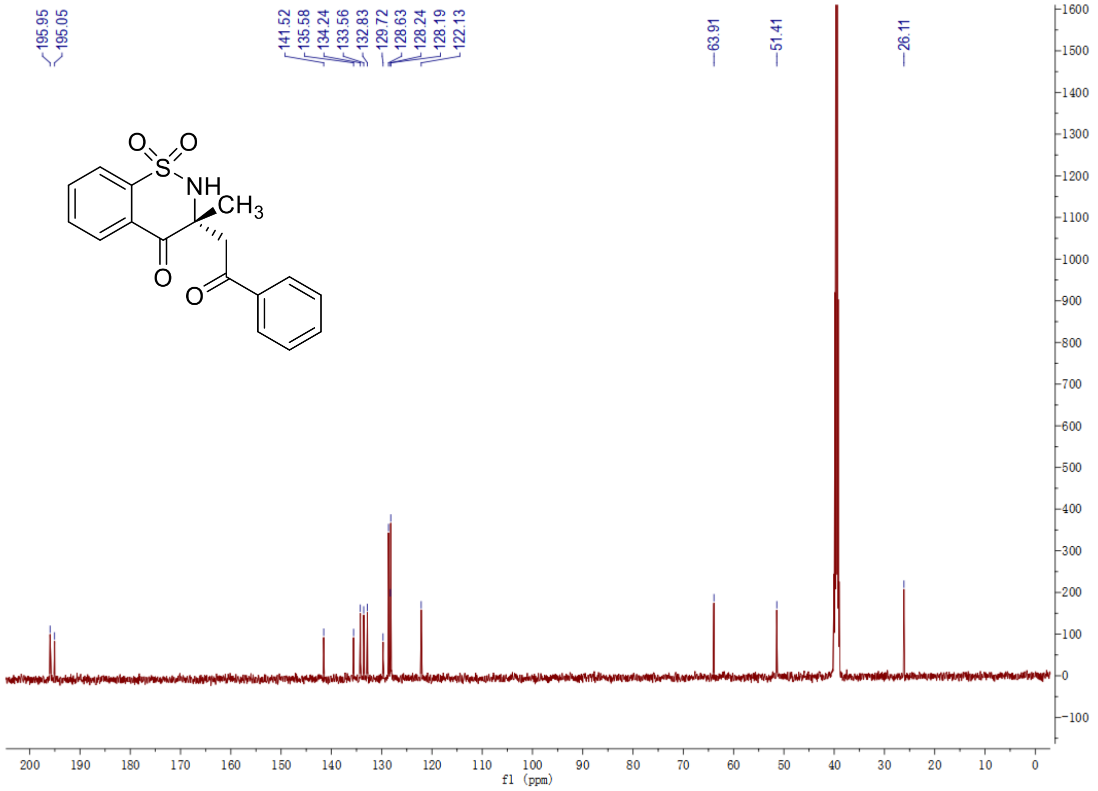
195.95
195.05
141.52
135.58
134.24
133.56
132.83
129.72
128.63
128.24
128.19
122.13
63.91
51.41
26.11
O
S
O
NH
CH3
O
O
f1 (ppm)

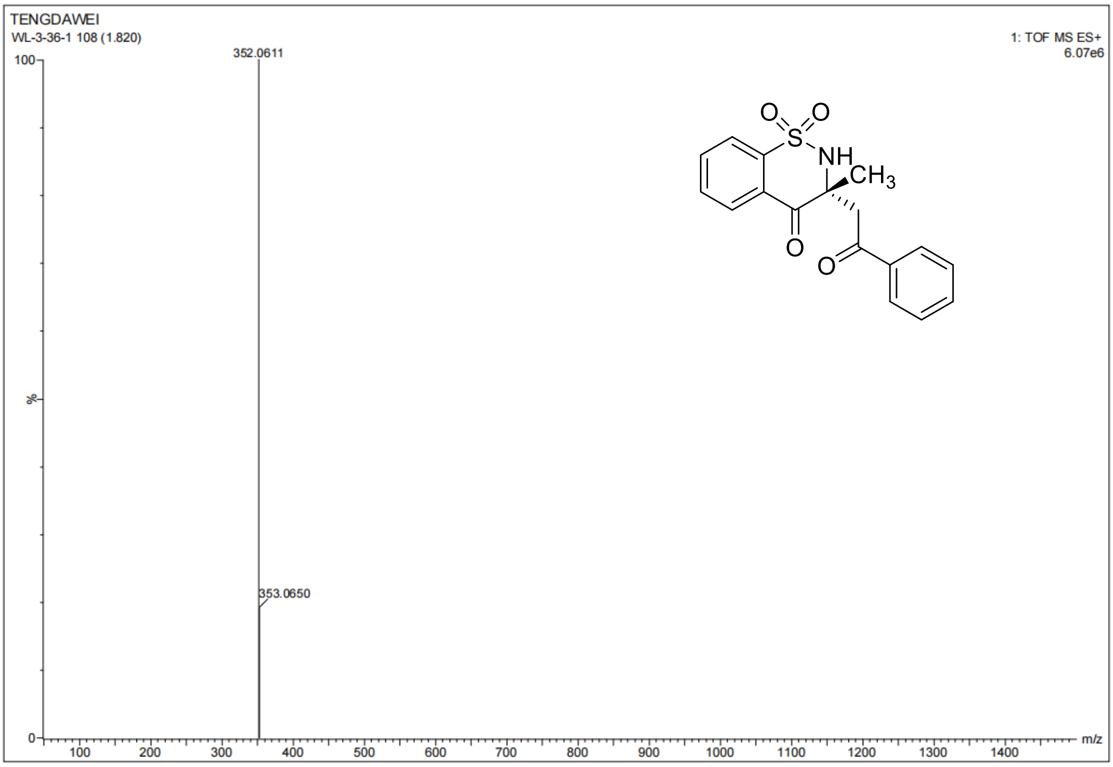
TENGDAWEI
WL-3-36-1 108 (1.820)
1: TOF MS ES+
6.07e6
352.0611
353.0650
%
m/z
O
S
O
NH
CH3
O
O

^{1}H NMR chromatograms of compound 3 and its diastereoisomer

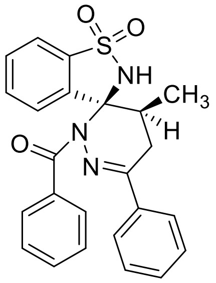

3aa
dr = 15.6:1

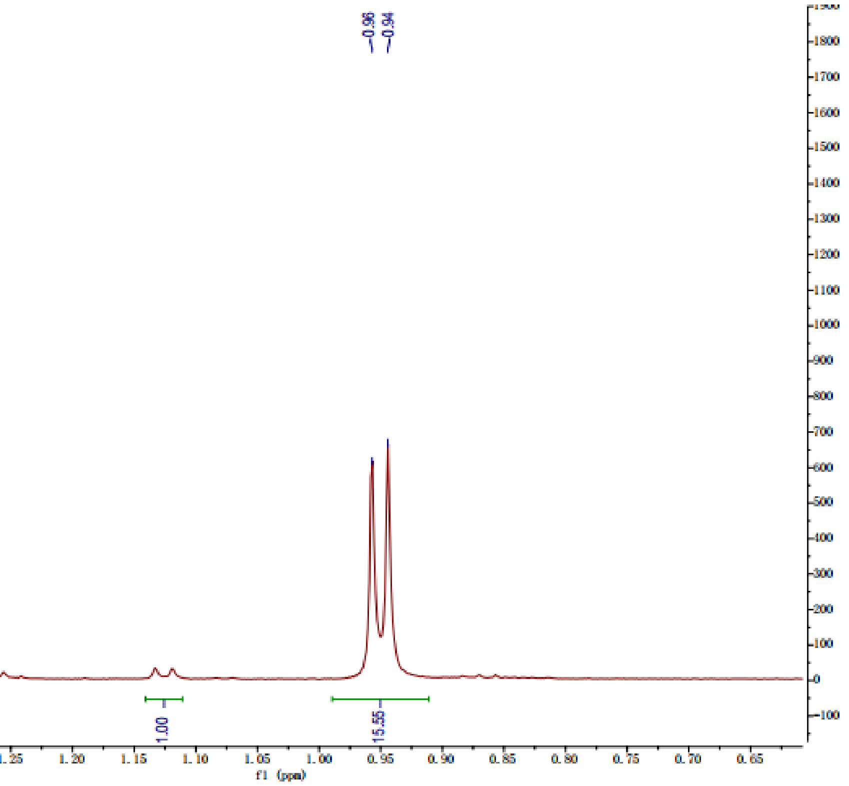

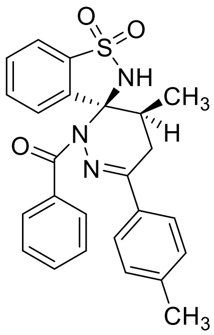

3ab
dr = 35.3:1

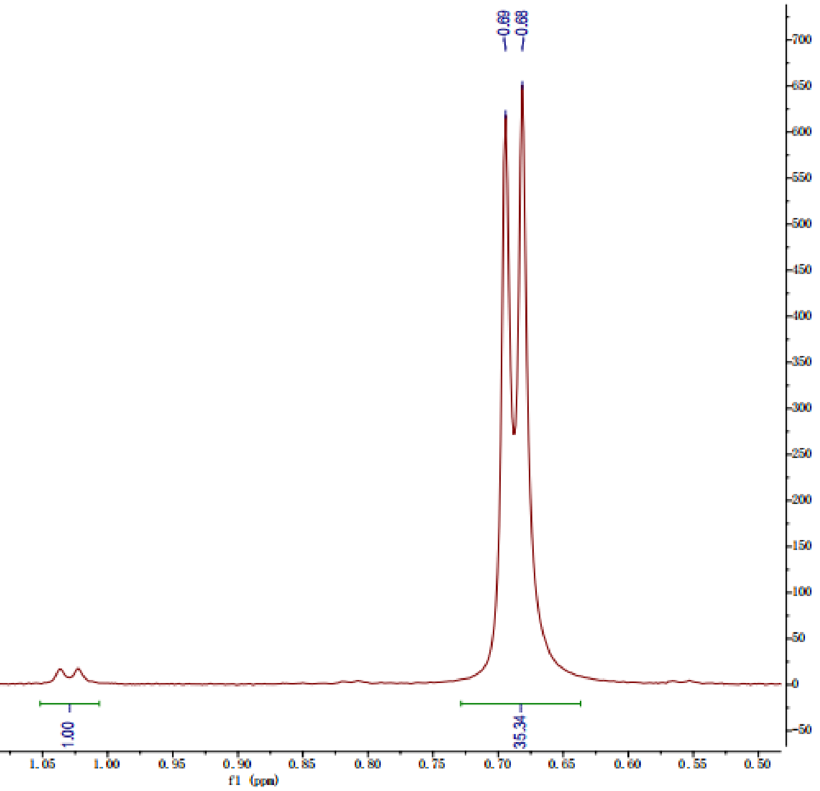

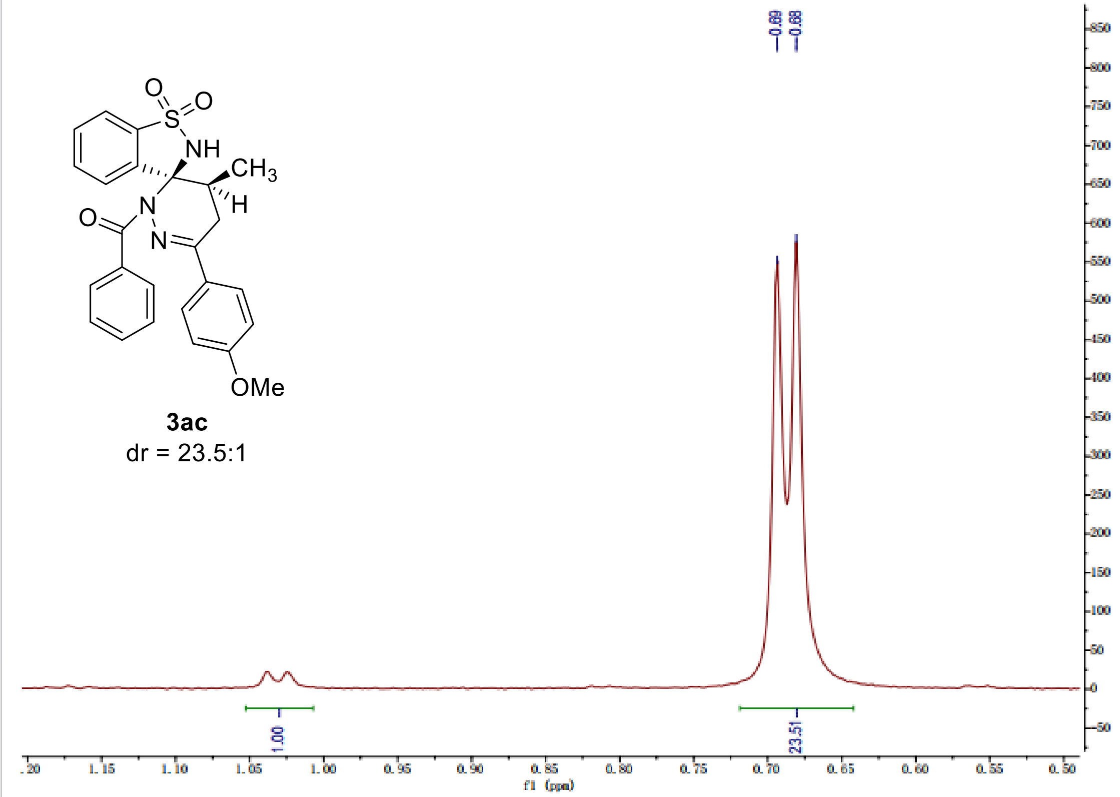

3ac
dr = 23.5:1

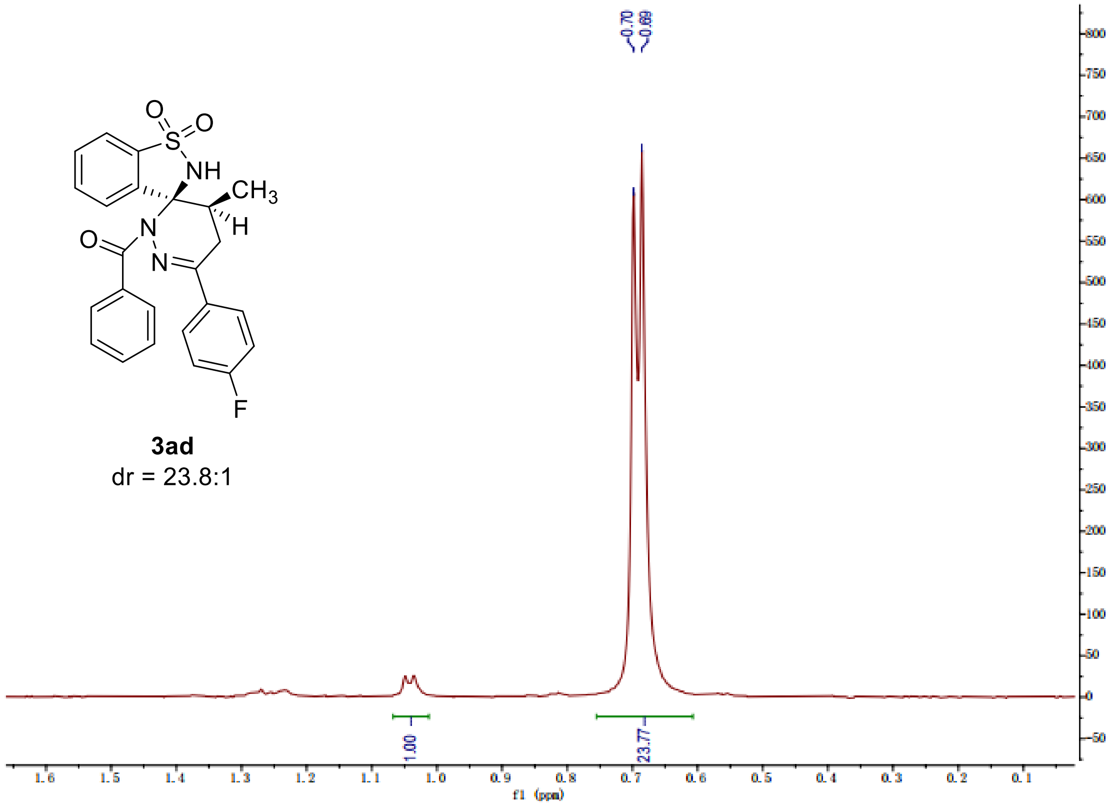

3ad
dr = 23.8:1

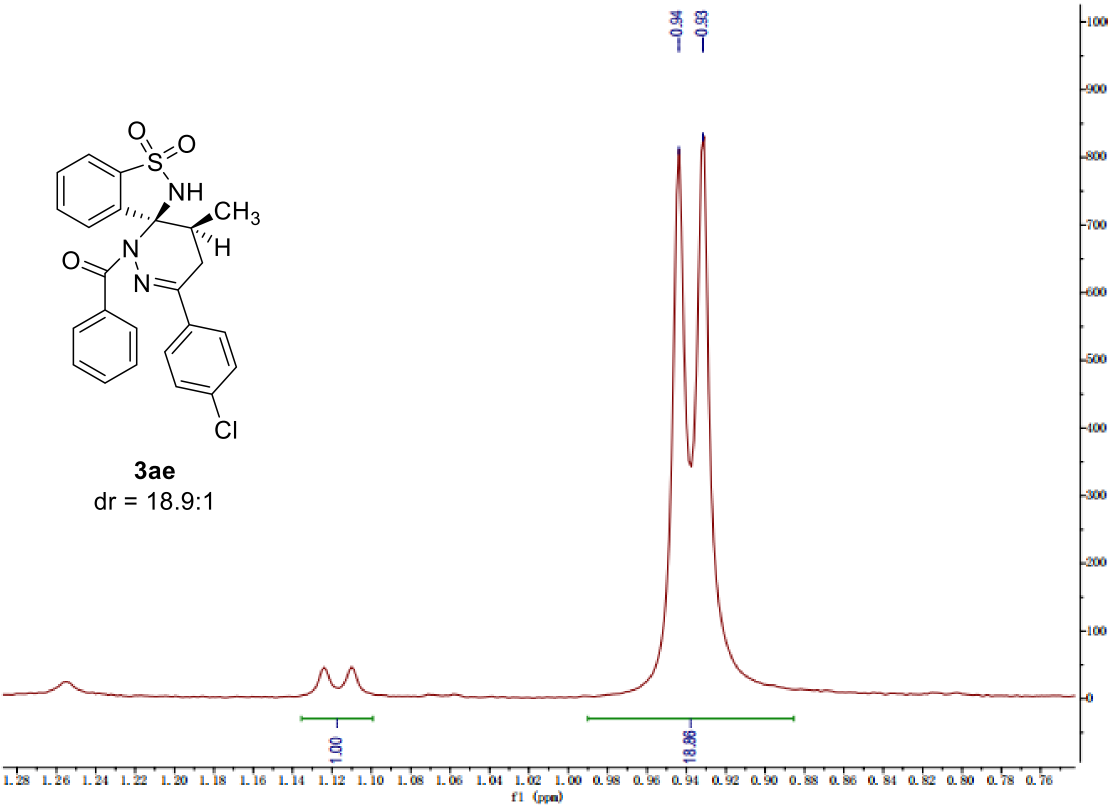
3ae
dr = 18.9:1
0.94
0.93
1.00
18.86
f1 (ppm)

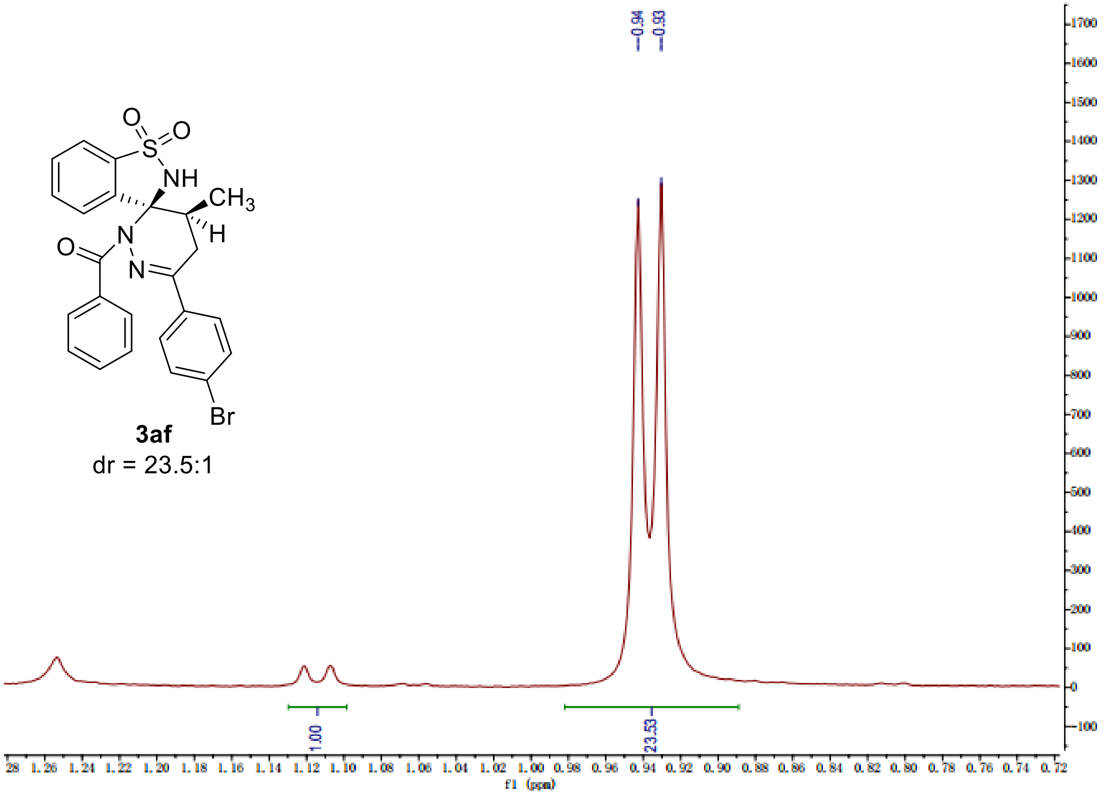
3af
dr = 23.5:1
0.94
0.93
1.00
23.53
f1 (ppm)

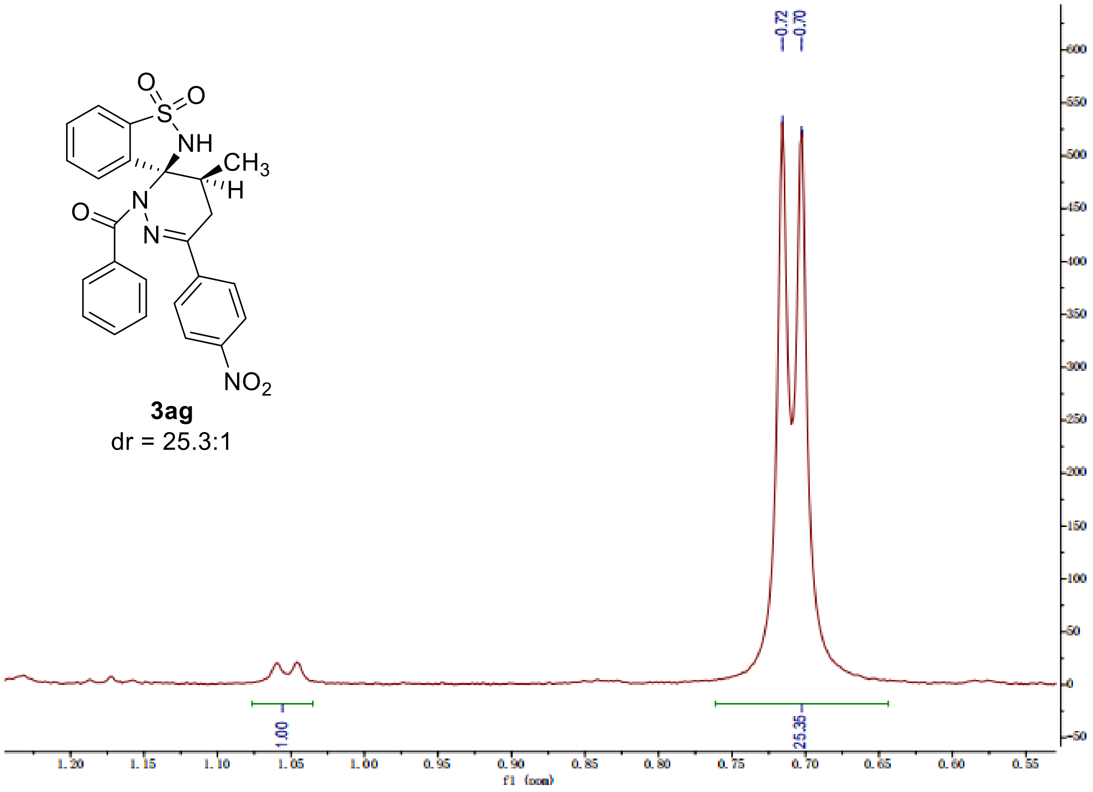

3ag
dr = 25.3:1

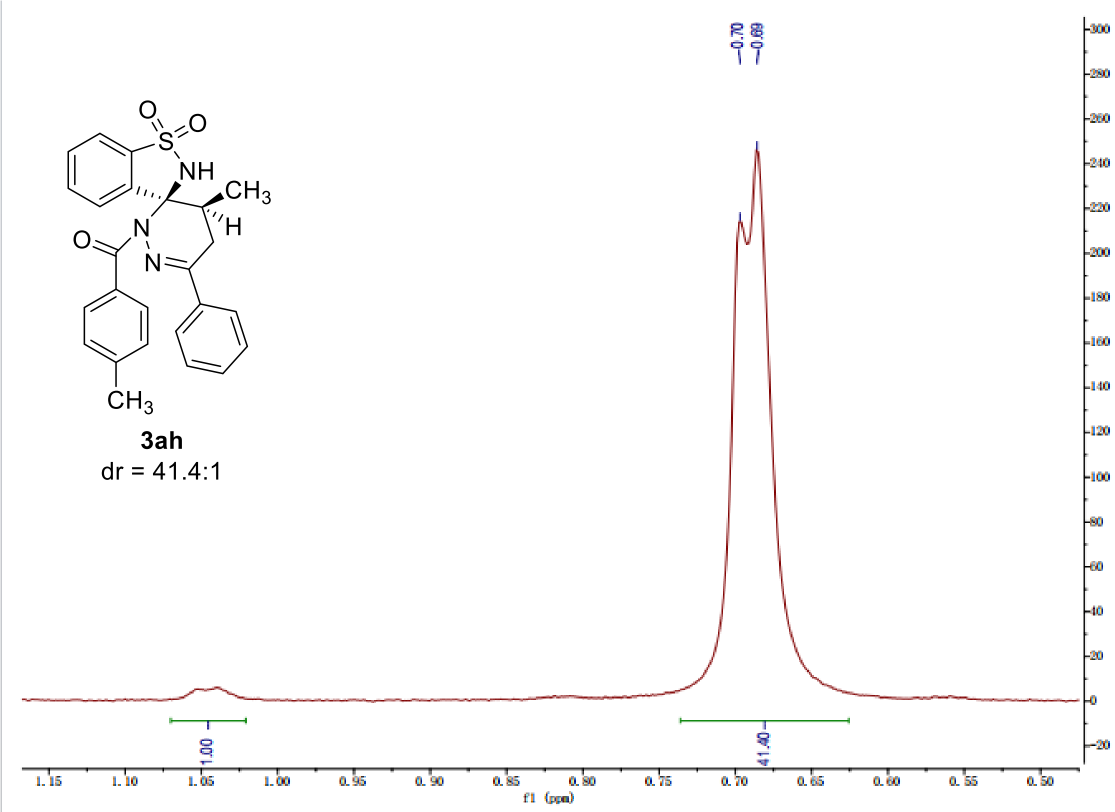

3ah
dr = 41.4:1

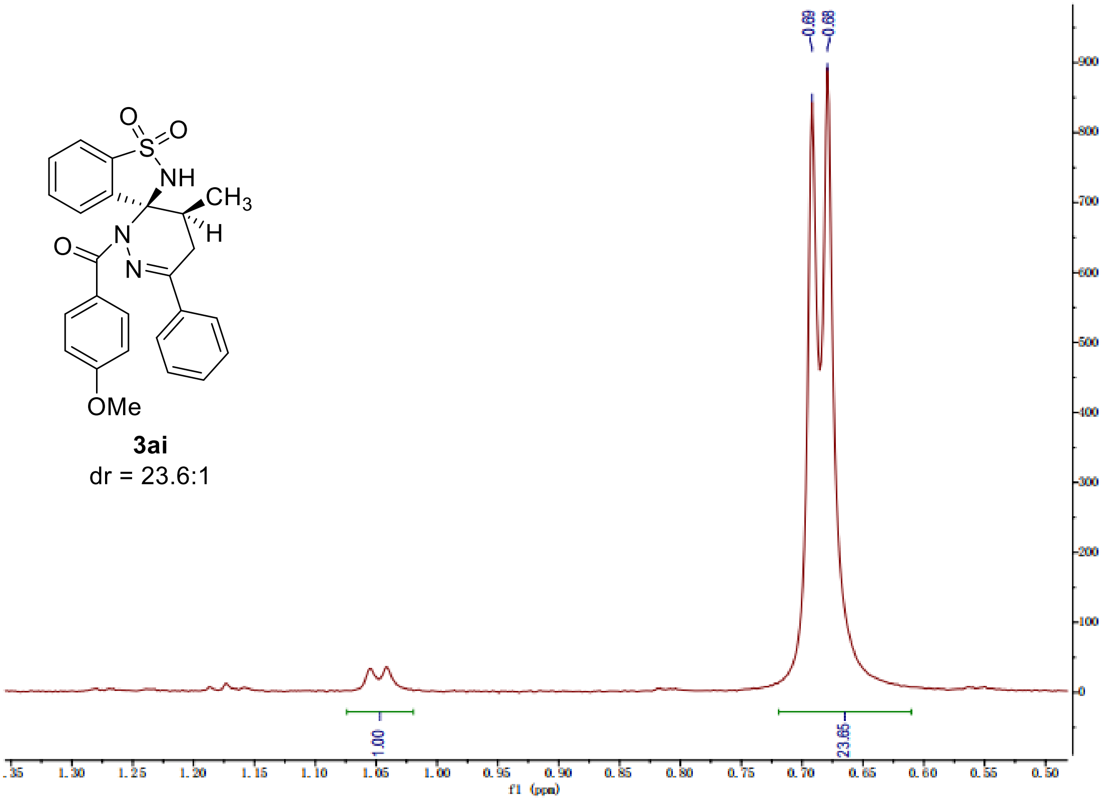

3ai
dr = 23.6:1

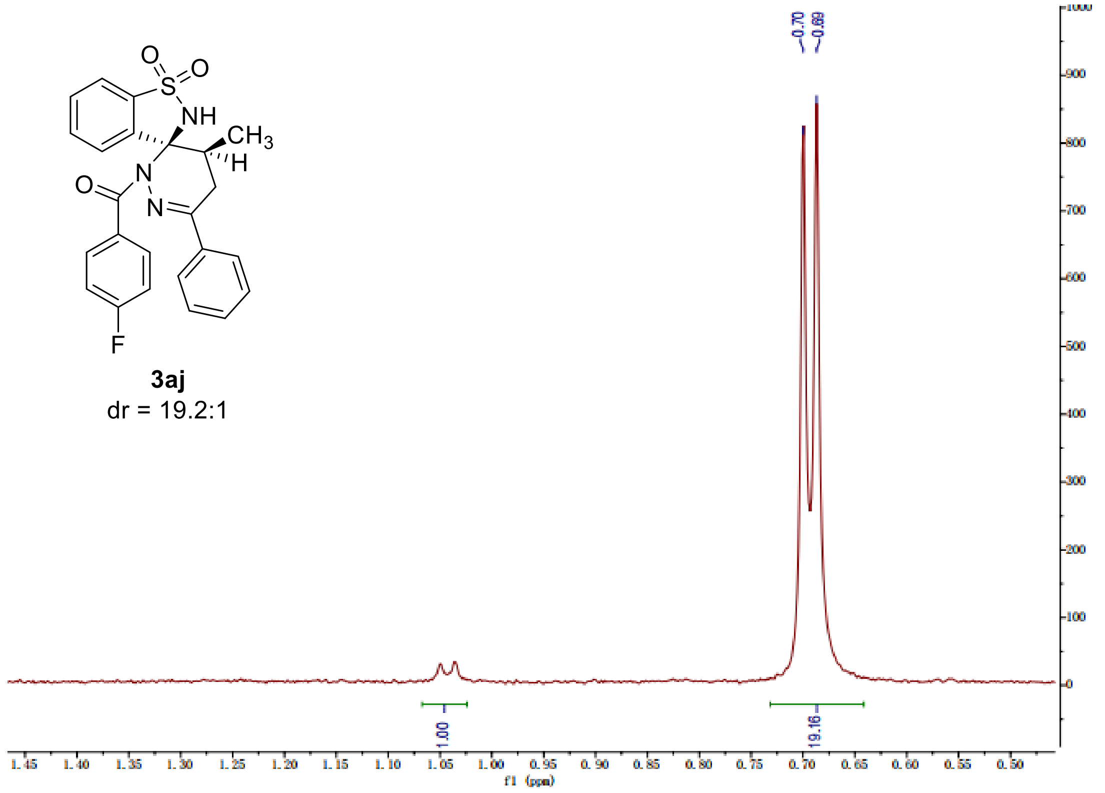

3aj
dr = 19.2:1

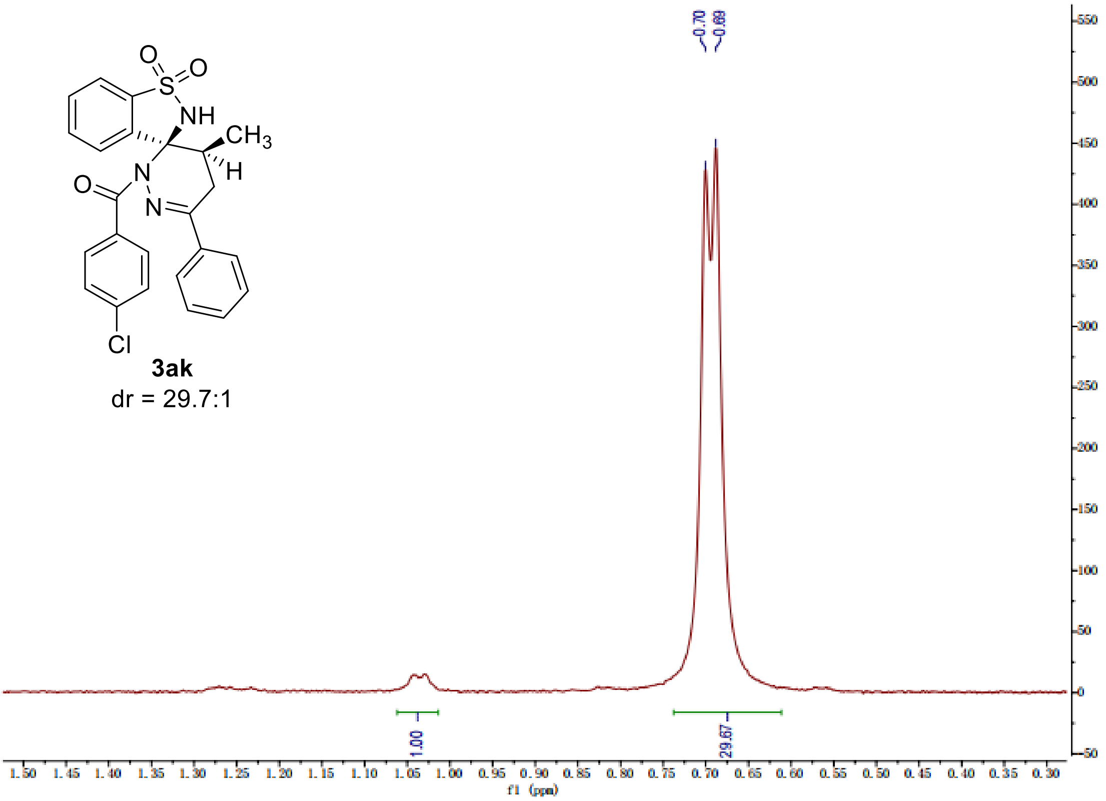
3ak
dr = 29.7:1
0.70
0.69
1.00
29.67
f1 (ppm)

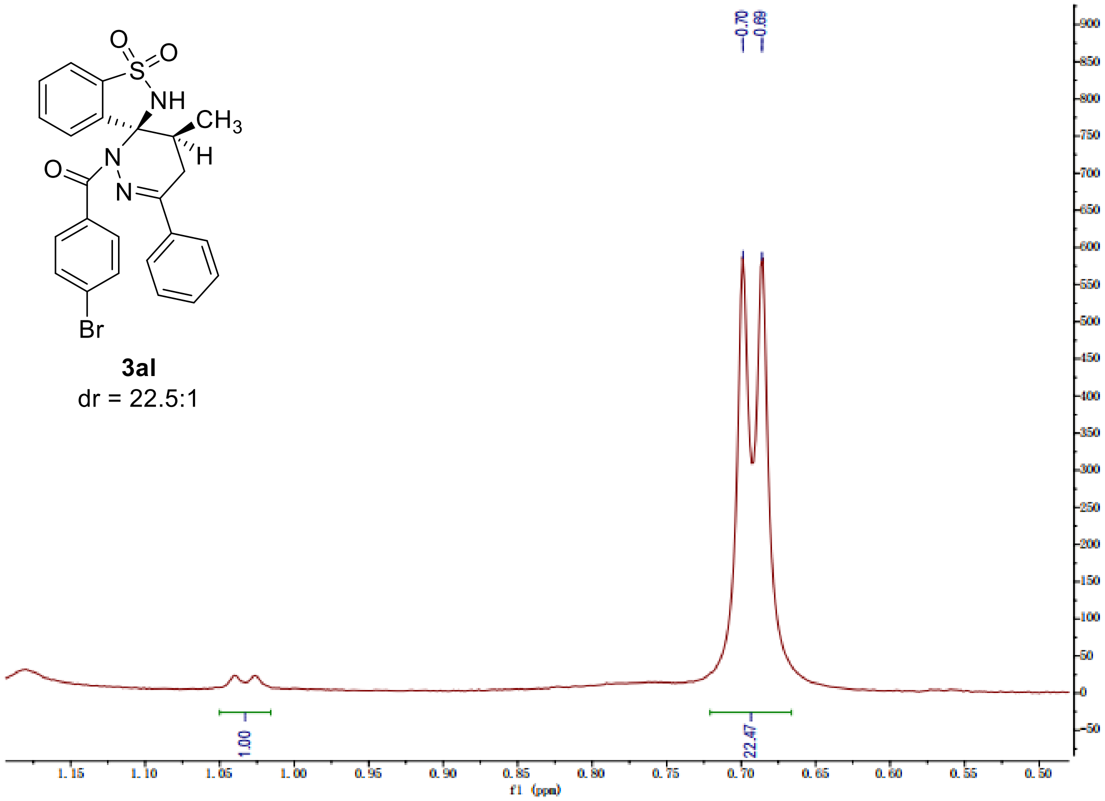
3al
dr = 22.5:1
0.70
0.69
1.00
22.47
f1 (ppm)

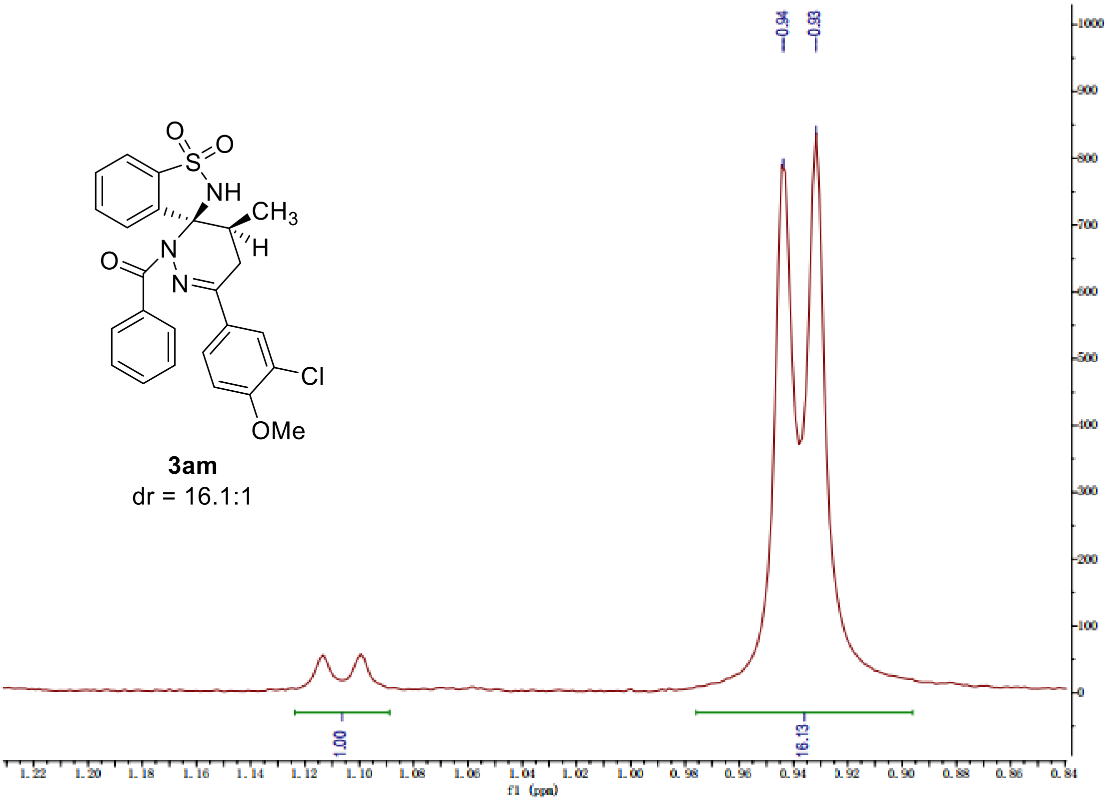

3am
dr = 16.1:1
